# Fine Properties of Geodesics and Geodesic $\lambda$-Convexity for the Hellinger–Kantorovich Distance

**DOI:** 10.1007/s00205-023-01941-1

**Published:** 2023-11-29

**Authors:** Matthias Liero, Alexander Mielke, Giuseppe Savaré

**Affiliations:** 1 Weierstraß-Institut für Angewandte Analysis und Stochastik, Berlin, Germany; 2 Humboldt Universität zu Berlin, Berlin, Germany; 3 Department of Decision Sciences and BIDSA, Bocconi University, Via Roentgen 1, 20136 Milan, Italy

## Abstract

We study the fine regularity properties of optimal potentials for the dual formulation of the Hellinger–Kantorovich problem ($\textsf{H}\!\!\textsf{K}$), providing sufficient conditions for the solvability of the primal Monge formulation. We also establish new regularity properties for the solution of the Hamilton–Jacobi equation arising in the dual dynamic formulation of $\textsf{H}\!\!\textsf{K}$, which are sufficiently strong to construct a characteristic transport-growth flow driving the geodesic interpolation between two arbitrary positive measures. These results are applied to study relevant geometric properties of $\textsf{H}\!\!\textsf{K}$ geodesics and to derive the convex behaviour of their Lebesgue density along the transport flow. Finally, exact conditions for functionals defined on the space of measures are derived that guarantee the geodesic $\lambda$-convexity with respect to the Hellinger–Kantorovich distance. Examples of geodesically convex functionals are provided.

## Introduction

In [26, 27] the Hellinger–Kantorovich distance (in [10, 11, 21] it is also called Wasserstein–Fisher–Rao distance or Kantorovich–Fisher–Rao distance in [17]) was introduced to describe the interaction between optimal transport and optimal creation and destruction of mass in a convex domain of $\mathbb {R}^d$. Here we further investigate the structure of (minimal) geodesics, and we fully analyze the question of geodesic $\lambda$-convexity of integral functionals with respect to this distance.

The Hellinger–Kantorovich distance can be considered as a combination, more precisely the inf-convolution, of the Hellinger–Kakutani distance on the set of all measures (cf. e.g.  [33]) and the $\textrm{L}^2$ Kantorovich–Wasserstein distance, which is well-known from the theory of optimal transport, see e.g. [2, 34]. Throughout this text, we denote by $\mathcal {M}({\mathbb {R}^{d}})$ all nonnegative and finite Borel measures endowed with the weak topology induced by the canonical duality with the continuous functions $\textrm{C}_0({\mathbb {R}^{d}})$ decaying at infinity. While the $\textrm{L}^2$ Kantorovich–Wasserstein distance $\textsf{W}(\mu _0,\mu _1)$ of measures $\mu _0$, $\mu _1 \in \mathcal {M}({\mathbb {R}^{d}})$ requires $\mu _0$ and $\mu _1$ to have the same mass to be finite, the Hellinger–Kakutani distance, which is defined via

$$\begin{aligned} \textsf{H}(\mu _0,\mu _1)^2 = \int _{\mathbb {R}^{d}}\big (\sqrt{\theta _0} - \sqrt{\theta _1}\big )^2 \;\!\textrm{d}(\mu _0{+}\mu _1), \text { where } \theta _j = \frac{\textrm{d}\mu _j}{\textrm{d}(\mu _0{+}\mu _1)}, \end{aligned}$$

has the upper bound $\textsf{H}(\mu _0,\mu _1)\leqq \mu _0({\mathbb {R}^{d}})+ \mu _1({\mathbb {R}^{d}})$, with equality if $\mu _0$ and $\mu _1$ are mutually singular.

As a generalization of the dynamical formulation of the Kantorovich–Wasserstein distance (see [6]), the Hellinger–Kantorovich distance $\textsf{H}\!\!\textsf{K}_{\alpha ,\beta }$ can be defined in a dynamic way via

1.1 $$\begin{aligned} \textsf{H}\!\!\textsf{K}_{\alpha ,\beta }(\mu _0,\mu _1)^2 = \inf \bigg \{&\int _{t=0}^1 \int _{\mathbb {R}^{d}}\big (\alpha |\Upsilon (t,x)|^2 {+}\beta \xi (t,x)^2\big ) \;\!\textrm{d}\mu _t(x) \;\!\textrm{d}t \nonumber \\&\bigg | \ \mu \in \textrm{C}\big ([0,1];\mathcal {M}({\mathbb {R}^{d}})\big ), \ \mu _{t=0}=\mu _0,\ \mu _{t=1}=\mu _1, \text { (gCE) holds} \bigg \}, \end{aligned}$$

where $\Upsilon :(0,1)\times {\mathbb {R}^{d}}\rightarrow \mathbb {R}^d$ and $\xi :(0,1)\times {\mathbb {R}^{d}}\rightarrow \mathbb {R}$ are Borel maps characterizing the generalized continuity equation

$$\begin{aligned} \text {(gCE) } \qquad \frac{\partial }{\partial t} \mu + \alpha \,\mathop {\textrm{div}}\big ( \mu \Upsilon \big ) =\beta \, \xi \mu , \end{aligned}$$

formulated in a distributional sense. The parameters $\alpha >0$ and $\beta >0$ allow us to control the relative strength of the Kantorovich–Wasserstein part and the Hellinger–Kakutani part, i.e. $\textsf{H}\!\!\textsf{K}_{\alpha ,\beta }$ is the inf-convolution of $\textsf{H}\!\!\textsf{K}_{\alpha ,0}= \frac{1}{\sqrt{\alpha }} \textsf{W}$ and $\textsf{H}\!\!\textsf{K}_{0,\beta }= \frac{1}{\sqrt{\beta }}\textsf{H}$, see [27, Rem. 8.19]. Subsequently, we will restrict to the standard case $\alpha =1$ and $\beta =4$, since the general case can easily be obtained by scaling the underlying space ${\mathbb {R}^{d}}$. We will shortly write $\textsf{H}\!\!\textsf{K}$ instead of $\textsf{H}\!\!\textsf{K}_{1,4}$.

It is a remarkable fact, deeply investigated in [27], that the $\textsf{H}\!\!\textsf{K}$ distance has many interesting equivalent characterizations, which highlight its geometric and variational character. A first one arises from the dual dynamic counterpart of (1.1) in terms of subsolutions of a suitable Hamilton–Jacobi equation:

1.2 $$\begin{aligned} \frac{1}{2}\textsf{H}\!\!\textsf{K}^2(\mu _0,\mu _1)= & {} \sup \bigg \{\int _{\mathbb {R}^{d}}\xi (\tau ,\cdot )\;\!\textrm{d}\mu _1 -\int _{\mathbb {R}^{d}}\xi (0,\cdot )\;\!\textrm{d}\mu _0\,\Big |\, \xi \in \textrm{C}^\infty _\textrm{c}([0,1]\times \mathbb {R}^d), \nonumber \\{} & {} \qquad \quad \frac{\partial }{\partial t}\xi + \frac{1}{2} |\nabla \xi |^2+2 \xi ^2\leqq 0 \quad \text {in }[0,1]\times \mathbb {R}^d\bigg \}. \end{aligned}$$

By expressing solutions of (1.2) in terms of a new formula of Hopf–Lax type, one can write a static duality representation

1.3 $$\begin{aligned} \textsf{H}\!\!\textsf{K}^2(\mu _0,\mu _\tau )&= \sup \Big \{\int _{\mathbb {R}^{d}}(1-\textrm{e}^{-2\varphi _\tau }) \;\!\textrm{d}\mu _\tau -\int _{\mathbb {R}^{d}}(\textrm{e}^{2\varphi _0}-1) \;\!\textrm{d}\mu _0\,\Big | \,\nonumber \\&\hspace{5em}\varphi _0,\varphi _\tau \in \textrm{C}_b({\mathbb {R}^{d}}),~ \varphi _\tau (x_\tau ){-}\varphi _0(x_0)\leqq \textrm{L}_1(x_\tau {-}x_0)\Big \} \end{aligned}$$

associated with the convex cost function $\textrm{L}_1(z):=\frac{1}{2}\log (1+\tan ^2(|z|))$ which forces $|z|<\pi /2$. Notice that it is possible to write (1.3) in a symmetric form with respect to $\varphi _0,\varphi _1$ just by changing the sign of $\varphi _1$.

It is remarkable that (1.3) can be interpreted as the dual problem of the static Logarithmic Entropy Transport (LET) variational formulation of $\textsf{H}\!\!\textsf{K}$. By introducing the logarithmic entropy density $F:[0,\infty [\rightarrow [0,\infty [{}$ via

1.4 $$\begin{aligned} F(s):= s\log s - s + 1\quad \text {for}\, s>0\quad \text {and} \quad F(0):=1, \end{aligned}$$

we get

1.5 $$\begin{aligned} \textsf{H}\!\!\textsf{K}^2(\mu _0,\mu _\tau )= \min \Big \{ \int _{\mathbb {R}^{d}}F(\sigma _0)\;\!\textrm{d}\mu _0+ \int _{\mathbb {R}^{d}}F(\sigma _1)\;\!\textrm{d}\mu _1+ \iint _{{\mathbb {R}^{d}}\times {\mathbb {R}^{d}}}2\textrm{L}_1(x_0{-}x_1)\;\!\textrm{d}{\varvec{\eta }}\Big \}\nonumber \\ \end{aligned}$$

where the minimum is taken over all positive finite Borel measures ${\varvec{\eta }}$ in $\mathbb {R}^d\times \mathbb {R}^d$ whose marginals $(\pi _i)_\sharp {\varvec{\eta }}=\sigma _i\mu _i$ are absolutely continuous with respect to $\mu _i$.

The subdifferential

$$\begin{aligned} \textrm{D}\textrm{L}_1(z)= \partial \textrm{L}_1(z)= {\textbf {tan}}(z):= \tan \big (|z|)\,\frac{z}{|z|} \end{aligned}$$

and its inverse $w \mapsto \textbf{arctan}(w)$ will play an important role. We continue to use bold function names for vector-valued functions constructed from real-valued ones as follows:

1.6 $$\begin{aligned} \text {for a map}\, f:\mathbb {R}\rightarrow \mathbb {R}\,\text {with}\, f(0)=0 \,\text {we set}\, \varvec{f}: \mathbb {R}^d \rightarrow \mathbb {R}^d \,\text {via}\, \varvec{f}(x):=f(|x|) \frac{x}{|x|}. \nonumber \\ \end{aligned}$$

A fourth crucial formula, which we will extensively study in the present paper, is related to the primal Monge formulation of Optimal Transport, and clarifies the two main components of $\textsf{H}\!\!\textsf{K}$ arising from transport and growth or decay effects. Its main ingredient is the notion of transport-growth pair $(\varvec{T},q):\mathbb {R}^d\rightarrow \mathbb {R}^d\times [0,\infty )$ acting on measures $\mu \in \mathcal {M}(\mathbb {R}^d)$ as

1.7 $$\begin{aligned} (\varvec{T},q)_\star \mu:= & {} \varvec{T}_\sharp (q^2\cdot \mu ), \nonumber \\ \big ((\varvec{T},q)_\star \mu \big )(A):= & {} \int _{\varvec{T}^{-1}(A)}q^2\;\!\textrm{d}\mu \quad \text {for every Borel set }\,A\subset \mathbb {R}^d. \end{aligned}$$

The Monge formulation of $\textsf{H}\!\!\textsf{K}$ then looks for the optimal pair $(\varvec{T},q)$ among the ones transforming $\mu _0$ into $\mu _1$ by $(\varvec{T},q)_\star \mu _0=\mu _1$ which minimizes the conical cost

1.8 $$\begin{aligned} {\mathcal {C}}(\varvec{T},q;\mu _0):=\int _{{\mathbb {R}^{d}}} \Big (1+q^2(x)-2q(x) \cos _{\pi /2}\big (|\varvec{T}(x){-}x|\big )\Big )\;\!\textrm{d}\mu _0(x), \end{aligned}$$

where $\cos _{\pi /2}(r):=\cos \big ( \min \{r,{\pi /2}\}\big )$. As for the usual Monge formulation of optimal transport, the existence of an optimal transport-growth pair $(\varvec{T},q)$ minimizing (1.8) requires more restrictive properties on $\mu _0,\mu _1$ which we will carefully study. It is worth noticing that the integrand in (1.8) has a relevant geometric interpretation as the square distance $\textsf{d}^2_{\pi ,\mathfrak {C}}$, where $\textsf{d}_{\pi ,\mathfrak {C}}$ is the distance on the cone space $\mathfrak {C}$ over $\mathbb {R}^d$ (cf. (2.5)) between the points [*x*, 1] and $[\varvec{T}(x),q(x)]$ and suggests that $\textsf{H}\!\!\textsf{K}$ induces a distance in $\mathcal {M}(\mathbb {R}^d)$ which plays a similar role than the $\textrm{L}^2$ Kantorovich–Rubinstein–Wasserstein distance in ${\mathcal {P}}_2(\mathbb {R}^d)$. The dynamic formulation (1.1), moreover, suggests that its minimizers $(\mu _t)_{t\in [0,1]}$ should provide minimal geodesics in $(\mathcal {M}(\mathbb {R}^d),\textsf{H}\!\!\textsf{K})$ which behave like transport-growth interpolations between $\mu _0$ and $\mu _1$.

Inspired by the celebrated paper [28], we want to study the structure of such minimizers and to characterize integral functionals which are convex along such kind of interpolations.

### Improved Regularity of Potentials and Geodesics

In the first part of the paper we will exploit the equivalent formulations of $\textsf{H}\!\!\textsf{K}$ in order to obtain new information on the regularity and on the fine structure of the solutions to (1.3), (1.2), and (1.8).

More precisely, we will initially prove in Section 3 that the optimal $\textsf{H}\!\!\textsf{K}$ potential $\varphi _0$ is locally semi-convex outside a closed $(d{-}1)$-rectifiable set, so that when $\mu _0\ll \mathcal {L}^{d}$ and $\mu _1$ is concentrated in a neighborhood of ${\text {supp}}(\mu _0)$ of radius ${\pi /2}$ the Monge formulation (1.8) has a unique solution.

After the transformation $\xi _0:=\frac{1}{2}(\textrm{e}^{\varphi _0}-1)$ (which linearizes the second integrand in the duality formula (1.3)), we also obtain a family of maps, for $t\in [0,1]$,

1.9 $$\begin{aligned} \varvec{T}_{0\rightarrow t}(x)=x+\textbf{arctan}\Big (\frac{t\nabla \xi _0}{1{+}2t\xi _0(x)}\Big ),\quad q^2_{0\rightarrow t}(x):=(1{+}2t\xi _0(x))^2+t^2|\nabla \xi _0(x)|^2,\nonumber \\ \end{aligned}$$

with the following properties:

1. $(\varvec{T}_{0\rightarrow 1},q_{0\rightarrow 1})$ is the unique solution of (1.8) and provides the beautiful formula
  1.10 $$\begin{aligned} \textsf{H}\!\!\textsf{K}^2(\mu _0,\mu _1)= \int _{\mathbb {R}^d} \Big (4\xi _0^2+|\nabla \xi _0|^2\Big )\;\!\textrm{d}\mu _0, \end{aligned}$$
  showing that the (closure of the) space of $\mathrm C^1_c(\mathbb {R}^d)$ functions with respect to the Hilbertian norm
  1.11 $$\begin{aligned} \Vert \xi \Vert _{H^{1,2}(\mathbb {R}^d,\mu )}^2= \int _{\mathbb {R}^d}\Big (4\xi ^2+|\nabla \xi |^2\Big )\;\!\textrm{d}\mu \end{aligned}$$
  provides the natural notion of tangent space $\textrm{Tan}_\mu \mathcal {M}(\mathbb {R}^d)$ and a nonsmooth Riemannian formalism in $(\mathcal {M}(\mathbb {R}^d),\textsf{H}\!\!\textsf{K})$ as for the Otto calculus in $({\mathcal {P}}_2(\mathbb {R}^d),W_2)$.
2. The curve $\mu _t=(\varvec{T}_{0\rightarrow t},q_{0\rightarrow t})_\star \mu _0$ is an explicit characterization of the geodesic interpolation solving (1.1). A crucial fact is that for $\mu _0$-a.e. *x* the curve $[\varvec{T}_{0\rightarrow t}(x),q_{0\rightarrow t}(x)]$ is a geodesic in the cone space $\mathfrak {C}$ interpolating the points [*x*, 1] and $[\varvec{T}_{0\rightarrow 1}(x),q_{0\rightarrow 1}(x)]$.

It is then natural to investigate if the potential $\xi _0$ can be used to build an optimal solution $\xi _t$ of (1.2), which should at least formally solve the Hamilton-Jacobi equation

1.12 $$\begin{aligned} \partial _t\xi _t+\frac{1}{2}|\nabla \xi _t|^2+2\xi _t^2=0\quad \text {on the support of} \,\mu \, \text {in}\, (0,1)\times \mathbb {R}^d. \end{aligned}$$

This problem will be investigated in Section 4, by a detailed analysis of the regularity of the forward solutions to (1.2) provided by the generalized Hopf–Lax formula (see (4.2))

1.13 $$\begin{aligned} \xi _t(x)=\xi (t,x) = \big ({\mathscr {P}}_{\hspace{-2.0pt}t}\hspace{1.0pt} \xi _0\big )(x)= \frac{1}{t}\quad {\mathscr {P}}_{\hspace{-2.0pt}1}\hspace{1.0pt}\big (t\xi _0(\cdot )\big )(x)=\inf _{y\in {\mathbb {R}^{d}}} \frac{1}{2t} \Big (1-\frac{\cos ^2_{{\pi /2}}\!{\left( |x{-}y|\right) }}{1+ 2t\xi _0(y)}\Big ).\nonumber \\ \end{aligned}$$

It is well known that one cannot expect smoothness of such a solution; however, the particular structure of transport duality suggests that the final value $\xi _1$ given by (1.13) corresponds to the optimal potential $\varphi _1$ of the dual formulation (1.3) via the transformation $\xi _1=\frac{1}{2}(1-\mathrm e^{-2\varphi _1})$, so that the initial and final optimal potentials $\xi _0$ and $\xi _1$ are simultaneously linked by the forward-backward relation

1.14 $$\begin{aligned} \xi _1={\mathscr {P}}_1\xi _0,\quad \xi _0={\mathscr {R}}_1(\xi _1)\quad \text {where } {\mathscr {R}}_t(\eta ):=-{\mathscr {P}}_t(-\eta )\text { is the backward flow.}\nonumber \\ \end{aligned}$$

Following the approach of [34, Cha. 7] (see also [27, Sec. 8]) and using the reversibility in time of geodesics, we can add to the family of forward potentials $\xi _t$ given by (1.13) the crucial information provided by the backward solutions ${\bar{\xi }}_t$ starting from $\xi _1$:

1.15 $$\begin{aligned} {\bar{\xi }}_t:={\mathscr {R}}_{1-t}\xi _1= -{\mathscr {P}}_{1-t}\big ({-}\xi _1\big )\quad \text {for } t\in [0,1]. \end{aligned}$$

In general, $\xi _t$ and ${\bar{\xi }}_t$ do not coincide for $t\in (0,1)$ but still satisfy

1.16 $$\begin{aligned} \xi _t(x)\geqq {\bar{\xi }}_t(x)\quad \text {in }(0,1)\times \mathbb {R}^d,\quad \xi _0={\bar{\xi }}_0,\quad \xi _1={\bar{\xi }}_1. \end{aligned}$$

The crucial fact arising from the optimality condition (1.14), and the geometric property of the geodesic $(\mu _t)_{t\in [0,1]}$ is that for every $t\in [0,1]$

$$\begin{aligned} \text {the support of}\, \mu _t\, \text {is contained in the}\, {contact set} \quad \Xi _t:= \big \{\, x\in \mathbb {R}^d \, \big | \, \xi _t(x)={\bar{\xi }}_t(x) \,\big \} . \end{aligned}$$

On the contact set $(\Xi _t)_{t\in [0,1]}$, we can combine the (delicate) first- and second-order super-differentiability properties of $\xi _t$ arising from the inf-convolution structure of (1.13) with the corresponding sub-differentiability properties exhibited by ${\bar{\xi }}_t$.

Using tools from nonsmooth analysis, we are then able to give a rigorous meaning to the characteristic flow associated with (1.12), i.e. to the maps $t\mapsto \varvec{T}(t,\cdot )=\varvec{T}_{s\rightarrow t}(\cdot )$, $t\mapsto q(t,\cdot )=q_{s\rightarrow t}(\cdot )$ solving (we omit to write the explicit dependence on *x* when not needed)

1.17 $$\begin{aligned} \left\{ \begin{aligned}&{\dot{\varvec{T}}}(t)=\nabla \xi _t(\varvec{T}(t)),\\&\dot{q}(t)=2\xi _t(\varvec{T}(t))q(t), \end{aligned} \right. \quad \text {in }(0,1),\quad \varvec{T}(s,x)=x,\ q(s,x)=1. \end{aligned}$$

Moreover, we will prove that $\varvec{T}_{s\rightarrow t}$ is a family of bi-Lipschitz maps on the contact sets obeying a natural concatenation property. As can be expected, the maps $\varvec{T}_{s\rightarrow t},q_{s\rightarrow t}$ provide a precise representation of the geodesics via $\mu _t=(\varvec{T}_{s\rightarrow t},q_{s\rightarrow t})_\star \mu _s$ for all $s,t\in (0,1)$. In particular $(\varvec{T}_{s\rightarrow t},q_{s\rightarrow t})$ is an optimal transport-growth pair between $\mu _s$ and $\mu _t$ minimizing the cost of (1.8).

Using this valuable information, in Section 5 we obtain various relevant structural properties of geodesics in $(\mathcal {M}(\mathbb {R}^d),\textsf{H}\!\!\textsf{K})$ such as *non-branching, localization, and regularization effects*. In particular, independently of the regularity of $\mu _0$ and $\mu _1$, we will show that for $s\in (0,1)$ the Monge problem between $\mu _s$ and $\mu _0$ or between $\mu _s$ and $\mu _1$ always admit a unique solution, a property which is well known in the Kantorovich–Wasserstein framework.

Surprisingly enough, despite the lack of global regularity, we will also establish precise formulae for the first and second derivative of the differential of $\varvec{T}_{s\rightarrow t}$ (and thus the second order differential of $\xi _t$) along the flow, which coincides with the equations that one obtains by formally differentiation using the joint information of the Hamilton–Jacobi equation (1.12) and (1.17) assuming sufficient regularity. For instance, differentiating in time the first equation of (1.17) and differentiating in space (1.12), one finds that

$$\begin{aligned} \ddot{\varvec{T}}(t)=\partial _t\nabla \xi _t(\varvec{T}(t))+\textrm{D}^2\xi _t\nabla \xi _t(\varvec{T}(t)),\quad \partial _t\nabla \xi _t= -\textrm{D}^2\xi _t\nabla \xi _t+4\xi _t\nabla \xi _t, \end{aligned}$$

which yield

1.18a $$\begin{aligned} \ddot{\varvec{T}}(t)=4\xi _t(\varvec{T}(t))\nabla \xi _t(\varvec{T}(t)). \end{aligned}$$

For *q*(*t*), ${\mathsf B}(t):=\textrm{D}\varvec{T}_{s\rightarrow t}$, and its determinant $\delta (t):=\det {\mathsf B}(t)$ similar, just more involved, calculations yield the crucial second order equations

1.18b $$\begin{aligned} \ddot{q}(t)&=|\nabla \xi _t(\varvec{T}(t))|^2q(t), \end{aligned}$$

1.18c $$\begin{aligned} \ddot{{\mathsf B}}(t)&=-4\Big (\nabla \xi _t\otimes \nabla \xi _t+\xi _t\textrm{D}^2\xi _t\Big )\circ \varvec{T}(t)\cdot {\mathsf B}(t), \end{aligned}$$

1.18d $$\begin{aligned} \ddot{\delta }(t)&=\Big ((\Delta \xi _t)^2-|\textrm{D}^2\xi _t|^2- 4|\nabla \xi _t|^2-4\xi _t\Delta \xi _t\Big )\circ \varvec{T}(t)\cdot \delta (t). \end{aligned}$$

In our case, even though we do not have enough regularity to justify the above formal computations, we can still derive them rigorously by a deeper analysis using the variational properties of the contact set. Even if our discussion is restricted to the Hellinger–Kantorovich case and uses the particular form of the Hopf–Lax semigroup (1.13) and its characteristics (1.9), we think that our argument applies to more general cases and may provide new interesting estimates also in the typical balanced case of Optimal Transport.

Such regularity and the related second order estimates are sufficient to express the Lebesgue density $c_t$ of the measures $\mu _t$ and thus to obtain crucial information on its behavior along the flow. In particular, Corollary 5.5 shows that $c(t,\cdot )$ is given by

1.19a $$\begin{aligned}&c(t, y) \big |_{y = \varvec{T}_{s\rightarrow t}(x)} = c(s,x) \frac{\alpha _s(t,x)}{\delta _s(t,x)} \quad \text {with } \end{aligned}$$

1.19b $$\begin{aligned}&\alpha _s(t,x)=(1{+}2(t{-}s)\xi _s(x))^2 + (t{-}s)^2 |\nabla \xi _s(x)|^2 =q_{s\rightarrow t}(x) \end{aligned}$$

1.19c $$\begin{aligned}&\delta _s(t,x):= \det ( \textrm{D}\varvec{T}_{s\rightarrow t}(x)), \end{aligned}$$

and the time-dependent transport-growth mapping $\varvec{T}_{s\rightarrow t},q_{s\rightarrow t}$ are given in terms of $\xi$ via (1.17) and the analog of (1.9). In particular, we will show that if $\mu _s\ll \mathcal {L}^{d}$ for some $s\in (0,1)$ then $\mu _t\ll \mathcal {L}^{d}$ for every $t\in (0,1)$ and combining (1.18b), (1.18c), and (1.19a) we will also prove that $c_t$ is a convex function along the flow maps $\varvec{T}_{s\rightarrow t}$.

### Geodesic $\lambda$-Convexity of Functionals

The second part of the paper is devoted to establish necessary and sufficient conditions for geodesic $\lambda$-convexity of energy functionals ${\mathscr {E}}$ defined for a closed and convex domain $\Omega \subset \mathbb {R}^d$ with non-empty interior in the form

1.20 $$\begin{aligned} {\mathscr {E}}(\mu )= \displaystyle \int _{\!\!\Omega } \! E(c(x)) \;\!\textrm{d}x + E'_\infty \mu ^\perp (\Omega )\quad \text {for} \quad \mu =c\mathcal {L}^{d}{+}\mu ^\perp \text { with } \mu ^\perp \perp \mathcal {L}^{d}, \qquad \end{aligned}$$

where $E'_\infty := \lim _{c\rightarrow \infty }E(c)/c\in \mathbb {R}\cup \{+\infty \}$ is the recession constant and $E(0)=0$ holds.

In [26, Prop. 19] it was shown that the total-mass functional ${\mathscr {M}}:\mu \mapsto \mu ({\mathbb {R}^{d}})$ has the surprising property that it is exactly quadratic along $\textsf{H}\!\!\textsf{K}$ geodesics $\gamma :[0,1]\rightarrow \mathcal {M}({\mathbb {R}^{d}})$, namely

1.21 $$\begin{aligned} {\mathscr {M}}(\gamma (t)) = (1{-}t){\mathscr {M}}(\gamma (0)) + t {\mathscr {M}}(\gamma (1)) - t(1{-}t) \textsf{H}\!\!\textsf{K}(\gamma (0),\gamma (1))^2 \ \text { for } t \in [0,1].\nonumber \\ \end{aligned}$$

Thus, as a first observation we see that a density function *E* generates a geodesically $\lambda$-convex functional ${\mathscr {E}}$ if and only if $E_0: c\mapsto E(c)- \lambda c$ generates a geodesically convex functional (i.e. geodesically 0-convex). Hence, subsequently we can restrict to $\lambda =0$.

To explain the necessary and sufficient conditions on *E* for ${\mathscr {E}}$ to be geodesically convex, we first look at the differentiable case, and we define the shorthand notation

$$\begin{aligned} \varepsilon _0(c)=E(c), \quad \varepsilon _1(c)=c E'(c), \quad \varepsilon _2(c) = c^2 E''(c). \end{aligned}$$

For the Kantorovich–Wasserstein distance $\textsf{W}$ the necessary and sufficient conditions are the so-called McCann conditions [28]:

1.22 $$\begin{aligned} \varepsilon _2(c)\geqq & {} \frac{d{-}1}{d} \big ( \varepsilon _1(c)-\varepsilon _0(c)\big ) \geqq 0 \text { for all }c>0\nonumber \\ {}\Longleftrightarrow & {} \left\{ \begin{array}{l} r \mapsto r^{d}E(r^{-d}) \text { is lower semi-continuous and convex and } \\ r \mapsto (d{-}1)r^{d}E(r^{-d}) \text { is non-increasing on } {]0,\infty [}, \end{array}\right. \qquad \end{aligned}$$

(see also [2, Prop. 9.3.9]). For the Hellinger–Kakutani distance we simply need the condition

1.23 $$\begin{aligned} 2 \varepsilon _2(c) + \varepsilon _1(c) \geqq 0 \quad \Longleftrightarrow \quad \Big ( r \mapsto E(r^2) \text { is convex }\Big ). \end{aligned}$$

In the case of differentiable *E*, our main result yields the following necessary and sufficient conditions for geodesic convexity of ${\mathscr {E}}$ on $(\mathcal {M}({\mathbb {R}^{d}}),\textsf{H}\!\!\textsf{K})$, see Proposition 6.1,

1.24 $$\begin{aligned} (d{-}1)\big (\varepsilon _1(c)-\varepsilon _0(c)\big )\geqq 0 \quad \text {and}\quad \mathbb {B}(c) \geqq 0 \quad \text {for all }c>0, \end{aligned}$$

where the matrix $\mathbb {B}(c)\in \mathbb {R}^{2\times 2}_\text {sym}$ is given by

$$\begin{aligned} \mathbb {B}(c):= \left( \begin{array}{cc} \varepsilon _2(c)-\frac{d-1}{d}\big (\varepsilon _1(c){-}\varepsilon _0(c)\big ) &{} \varepsilon _2(c) -\frac{1}{2}\big (\varepsilon _1(c){-}\varepsilon _0(c) \big ) \\ \varepsilon _2(c) -\frac{1}{2}\big (\varepsilon _1(c){-}\varepsilon _0(c) \big ) &{} \varepsilon _2(c) + \frac{1}{2} \varepsilon _1(c) \end{array} \right) . \end{aligned}$$

We immediately see that the non-negativity of the diagonal element $\mathbb {B}_{11}(c)$ gives the first McCann condition in (1.22), and $\mathbb {B}_{22}(c) \geqq 0$ gives (1.23). However, the condition $\mathbb {B}(c)\geqq 0$ is strictly stronger, since e.g. it implies that the additional condition $(d{+}2)\varepsilon _1(c)-2\varepsilon _0(c)\geqq 0$ holds, see (6.2). This condition means that $c \mapsto c^{-2/(d+2)}E(c)$ has to be non-decreasing, which will be an important building block for the main geodesic convexity result.

Indeed, our main result in Theorem 7.2 is formulated for general lower semi-continuous and convex functions $E:{[0,\infty [}\rightarrow \mathbb {R}\cup \{\infty \}$ without differentiability assumptions. The conditions on *E* can be formulated most conveniently in terms of the auxiliary function $N_E:{]0,\infty [}^2\rightarrow \mathbb {R}\cup \{\infty \}$ defined via

1.25a $$\begin{aligned} N_E(\rho ,\gamma )= \big (\frac{\rho }{\gamma }\big )^d E\Big (\frac{\gamma ^{2+d}}{\rho ^d}\Big ). \end{aligned}$$

Then, ${\mathscr {E}}$ defined in (1.20) is geodesically convex if and only if $N_E$ satisfies

1.25b $$\begin{aligned}&N_E:{]0,\infty [}^2\rightarrow \mathbb {R}\cup \{\infty \} \text { is convex, and} \end{aligned}$$

1.25c $$\begin{aligned}&\rho \mapsto (d{-}1)N_E(\rho ,\gamma ) \text { is non-increasing}. \end{aligned}$$

The McCann conditions (1.22) are obtained by looking at $N_E(\cdot ,\gamma )$ for fixed $\gamma$, while the Hellinger–Kakutani condition (1.23) follows by looking at $s \mapsto N_E(s\rho ,s\gamma )$ for fixed $(\rho ,\gamma )$.

The proof of the sufficiency and necessity of condition (1.25) for geodesic convexity of ${\mathscr {E}}$ is based on the explicit representation (1.19) of the geodesic curves giving

$$\begin{aligned} {\mathscr {E}}(\mu (t)){} & {} =\int _\Omega E(c(t,y))\;\!\textrm{d}y = \int _\Omega e(t,x) \;\!\textrm{d}x \\{} & {} \qquad \text { where } e(t,x):=\delta _s(t,x)\,E\Big (c_s(x)\frac{\alpha _s(t,x)}{\delta _s(t,x)}\Big ). \end{aligned}$$

By definition, we have $\alpha _s(t,x)\geqq 0$, and Corollary 5.5 guarantees $\delta _s(t,x)>0$. Hence, we can introduce the two functions

$$\begin{aligned} \gamma (t,x)=\big (c_s(x)\alpha _s(t,x)\big )^{1/2} \quad \text { and } \quad \rho (t,x) = \big (c_s(x)\alpha _s(t,x) \big )^{1/2} \delta _s(t,x)^{1/d}, \end{aligned}$$

which connect the densities *e*(*t*, *x*) with the function $N_E$ defined in (1.25a) in the form

$$\begin{aligned} e = \delta \,E\big (\,c\,\frac{\alpha }{\delta }\,\big ) = N_E(\rho ,\gamma ). \end{aligned}$$

For smooth *E* we have smooth $N_E$ and may show convexity of $t\mapsto e(t,x)$ via

$$\begin{aligned} \partial _t^2 e(t,x)&=:\ddot{e} = \Big \langle \textrm{D}^2 N_E(\rho ,\gamma ) \left( {\begin{array}{c}{\dot{\rho }}\\ {\dot{\gamma }}\end{array}}\right) , \left( {\begin{array}{c}{\dot{\rho }}\\ {\dot{\gamma }}\end{array}}\right) \Big \rangle + \Big \langle \textrm{D}N_E(\rho ,\gamma ), \left( {\begin{array}{c}\ddot{\rho }\\ \ddot{\gamma }\end{array}}\right) \Big \rangle \geqq 0. \end{aligned}$$

By convexity of $N_E$, the term involving $\textrm{D}^2 N_E$ is non-negative, so it remains to show

1.26 $$\begin{aligned} \partial _\rho N_E(\rho ,\gamma ) \ddot{\rho } + \partial _\gamma N_E(\rho ,\gamma ) \ddot{\gamma }\geqq 0. \end{aligned}$$

To establish this, we use first that the scaling property $N_E(s^{1+d/2}\rho ,s \gamma )=s^2 N_E(\rho ,\gamma )$ for all $s>0$ (which follows from the definition of $N_E$ via *E*) and the convexity of $N_E$ imply

1.27 $$\begin{aligned} (1{-}4/d^2) \;\! \rho \;\! \partial _\rho N_E(\rho ,\gamma )+ \gamma \;\! \partial _\gamma N_E(\rho ,\gamma )\geqq 0, \end{aligned}$$

see Proposition 6.2. Second, we rely on a nontrivial curvature estimate for $(\rho ,\gamma )$, namely

1.28 $$\begin{aligned} \frac{\ddot{\gamma }(t,x)}{\gamma (t,x)} \geqq 0 \quad \text {and} \quad \frac{\ddot{\rho }(t,x)}{\rho (t,x)} \leqq \left( 1-\frac{4}{d}\right) \!\; \frac{\ddot{\gamma }(t,x)}{\gamma (t,x)}. \end{aligned}$$

Estimates (1.28) are provided in Proposition 5.7 and strongly rely on the explicit representation and the regularity properties of the geodesics developed in Sects. 4 and 5.

Combining (1.28) with $\partial _\rho N_E(\rho ,\gamma )\leqq 0$, the desired relation (1.26) easily follows, see Section 7. Finally, a simple integration over ${\mathbb {R}^{d}}$ provides the convexity of $t \mapsto {\mathscr {E}}(\mu (t))$. Note that we have indeed the larger factor $(1{-}4/d^2)$ in (1.27) while the curvature estimate in (1.28) has the smaller and hence “better” factor $(1{-}4/d)$.

As a consequence, we find that the power functionals ${\mathscr {E}}_m$ with $E_m(c)=c^m$ with $m>1$ are all geodesically convex, see Corollary 7.3. This result was already exploited in [13, Thm. 2.14]. We can study the discontinuous “Hele–Shaw case” $E(c)=-\lambda c$ for $c\in [0,1]$ and $E(c)=\infty$ for $c>1$. Moreover, in dimensions $d=1$ or 2 the densities $E_q(c)=- c^q$ with $q\in [\frac{d}{d+2},\frac{1}{2}]$ also lead to geodesically convex functionals ${\mathscr {E}}_q$, see again Corollary 7.3.

Two important differences with the balanced Kantorovich–Wasserstein case are worth noting. First, the Boltzmann logarithmic entropy functional corresponding to $E(c)=c\log c$ is not geodesically $\lambda$-convex for any value of $\lambda$, see Example 6.5. Second, if the space dimension *d* is larger than or equal to 3, then there are no geodesically convex power functionals of the form $E(x)=-c^m$ with exponent $m<1$, see Example 6.4. Some of these statements follow easily by observing that $\mu _t = t^2 \mu _1$ is the unique geodesic connecting $\mu _0=0$ and $\mu _1$.

### Applications and Outlook

In [15, 23], the JKO scheme (minimizing movement scheme) for a gradient system $(\mathcal {M}(\Omega ),\textsf{H}\!\!\textsf{K}_{\alpha ,\beta },{\mathscr {E}})$ is considered, i.e., for $\tau >0$ we iteratively define

1.29 $$\begin{aligned} \mu _{k\tau } \in \mathrm {Arg\,Min} \Big \{\, \frac{1}{2\tau } \textsf{H}\!\!\textsf{K}^2_{\alpha ,\beta } ( \mu _{(k-1)\tau }, \mu ) + {\mathscr {E}}(\mu ) \; \Big | \; \mu \in \mathcal {M}(\Omega ) \,\Big \} \end{aligned}$$

and consider the limit $\tau \downarrow 0$ (along subsequences) to obtain *generalized minimizing movements* (GMM) (cf.  [2]). Under suitable conditions, including the assumption ${\mathscr {E}}(\mu )= \int _\Omega \big ( E(c) + c V\big ) \;\!\textrm{d}x$ with $\mu = c{\mathcal {L}}^d$ and *E* superlinear, it is shown in [15, Thm. 3.4] that all GMM $\mu$ have the form $\mu (t)=c(t) {\mathcal {L}}^d$, and the density *c* is a weak solution of the reaction-diffusion equation

$$\begin{aligned} \partial _t c{} & {} = \alpha \mathop {\textrm{div}}\nolimits \big ( c \nabla (E'(c){+}V) \big ) - \beta \,u\,\big (E'(c){+}V \big ) \ \text { in }\Omega , \\{} & {} \quad c \nabla (E'(c){+}V)\cdot \textrm{n}=0 \ \text { on }\partial \Omega . \end{aligned}$$

In [24], the equation $u_t =0= \Delta u + a u \log u + bu$ is studied, whose solutions are steady states for HK gradient flows for ${\mathscr {E}}(u)=\int _{\mathbb {R}^{d}}u\log u \;\!\textrm{d}x$. We also refer to [13, 32], where equation (1.29) was studied for $E(c)=\frac{1}{m} c^m - \lambda c$ and $V\equiv 0$. The linear functional $\Phi (\mu )=\int _{\mathbb {R}^{d}}V(x)\;\!\textrm{d}\mu$ for a given potential $V\in \textrm{C}^0({\mathbb {R}^{d}})$ can easily be added, as its geodesic $\lambda$-convexity is characterized in [26, Prop. 20]. Note that our main convexity result, proved here for the first time, plays an important role in the existence and uniqueness results of [13], cf. Thm. 2.14 there.

In [23] it is shown that the GMM for the gradient system $(\mathcal {M}(\Omega ),\textsf{H}\!\!\textsf{K}_{\alpha ,\beta },{\mathscr {E}})$ are EVI$_\lambda$ solutions in the sense of [30]. Again the main ingredient is the geodesic $\lambda$-convexity of ${\mathscr {E}}$ in the form (1.20) contained in our main Theorem 7.2.

**Table:** 

Main Notation	
$\mathcal {M}(X),\ \mathcal {M}_2(X)$	finite positive Borel measures on *X* (with finite quadratic moment)
$\mathcal {P}(X),\ \mathcal {P}_2(X)$	Borel probability measures on *X* (with finite quadratic moment)
$T_\sharp \mu$	push forward of $\mu \in \mathcal {M}(X)$ by a map $T:X\rightarrow Y$: (2.1)
$\mu =c\mathcal {L}^{d}+\mu ^\perp$	Lebesgue decomposition of a measure $\mu \mathcal {M}(\mathbb {R}^d)$
$\textrm{C}_\textrm{b}(X)$	continuous and bounded real functions on *X*
$\cos _a(r)$	truncated function $\cos \big (\min \{a,r\}\big )$, $a>0$ (typically $a=\pi /2$)
$\textsf{W}_X(\mu _1,\mu _2)$	Kantorovich–Wasserstein distance in ${\mathcal {P}}_2(X)$
${\textbf {sin}},{\textbf {tan}}, \textbf{arctan},\cdots$	vector-valued version of the usual scalar functions, see (1.6)
$\textsf{H}\!\!\textsf{K}(\mu _1,\mu _2)$	Hellinger–Kantorovich distance in $\mathcal {M}(X)$: Section 2
$(\mathfrak {C},\ \textsf{d}_{a,\mathfrak {C}}),\ \mathfrak {o}$	metric cone on $\mathbb {R}^d$ and its vertex, see Subsection 2.1.2
$\textsf{W}_{a,\mathfrak {C}}$	$L^2$-Kantorovich–Wasserstein distance on ${\mathcal {P}}_2(\mathfrak {C})$ induced by $\textsf{d}_{a,\mathfrak {C}}$
$\textsf{x},\textsf{r}$	coordinate maps on $\mathfrak {C}$, see Subsection 2.1.2
$\pi ^0,\pi ^1$	coordinate maps on a Cartesian product $X_0\times X_1$, $\pi ^i(x_0,x_1)=x_i$
$\mathfrak {h}$	homogeneous projection from $\mathcal {M}_2(\mathfrak {C})$ to $\mathcal {M}(\mathbb {R}^d)$, see (2.8)
$S_i, S_i', S_i'', S_i^{\pi /2},\mu _i',\mu _i''$	see (2.12)–(2.13)
$(\varvec{T},q)_\star$	action of a transport-growth map, Definition 2.7
$\textrm{AC}^p([0,1];X)$	space of curves $\textrm{x}:[0,1]\rightarrow X$ with *p*-integrable metric speed
	forward and backward $\mathbb {L}$-transform for cost function $\mathbb {L}$, see (3.3)
$D_i',D_i''$	domains of $\nabla \varphi _i$ and $\textrm{D}^2\varphi _i$, see Theorem 3.2
$\xi _s={\mathscr {P}}_{\hspace{-2.0pt}s}\hspace{1.0pt}\xi , {\bar{\xi }}_s={\mathscr {R}}_{\hspace{-2.0pt}s}\hspace{1.0pt}\bar{\xi }$	for- and backward solution of Hamilton–Jacobi equation, (4.2), (4.3)
$\Xi _s$	contact set of forward and backward solutions $\xi _s$, $\overline{\xi }_s$, see (4.39)
$(\varvec{T}_{s\rightarrow t}, q_{s\rightarrow t})$	transport-growth map induced by for/backward solutions, Theorem 4.3

## The Hellinger–Kantorovich Distance

In this section, we recall a few properties and equivalent characterizations of the Hellinger–Kantorovich distance from [26, 27], that will turn out to be crucial in the following.

First, we fix some notation that we will extensively use: Let $(X,\textsf{d}_X)$ be a complete and separable metric space. In the present paper *X* will typically be $\mathbb {R}^d$ with the Euclidean distance, a closed convex subset thereof, the cone space $\mathfrak {C}$ on $\mathbb {R}^d$ (see Subsection 2.1.2), product spaces of the latter two, etc. We will denote by $\mathcal {M}(X)$ the space of all non-negative and finite Borel measures on *X* endowed with the weak topology induced by the duality with the continuous and bounded functions of $\textrm{C}_\textrm{b}(X)$. The subset of measures with finite quadratic moment will be denoted by $\mathcal {M}_2(X)$. The spaces $\mathcal {P}(X)$ and $\mathcal {P}_2(X)$ are the corresponding subsets of probability measures.

If $\mu \in \mathcal {M}(X)$ and $T:X\rightarrow Y$ is a Borel map with values in another metric space *Y*, then $T_\sharp \mu$ denotes the push-forward measure on $\mathcal {M}(Y)$, defined by

2.1 $$\begin{aligned} T_\sharp \mu (B):=\mu (T^{-1}(B))\quad \text {for every Borel set}\, B\subset Y. \end{aligned}$$

We will often denote elements of $X\times X$ by $(x_0,x_1)$ and the canonical projections by $\pi ^i:(x_0,x_1)\rightarrow x_i$, $i=0,1$. A coupling on *X* is a measure $\varvec{\gamma }\in \mathcal {M}(X{\times } X)$ with marginals $\gamma _i:=\pi ^i_\sharp \varvec{\gamma }$.

Given two measures $\mu _0,\mu _1\in \mathcal {M}_2(X)$ with equal mass $\mu _0(X)=\mu _1(X)$, their (quadratic) Kantorovich–Wasserstein distance $\textsf{W}_X$ is defined by

2.2 $$\begin{aligned} \begin{aligned} \textsf{W}_{\hspace{-1.0pt}X}(\mu _0,\mu _1)^2:=\min \bigg \{\int \int&\textsf{d}_X(x_0,x_1)^2\;\!\textrm{d}\varvec{\gamma }(x_0,x_1)\,\Big |\, \\&\varvec{\gamma }\in \mathcal {M}(X{\times } X),\ \pi ^i_\sharp \varvec{\gamma }=\mu _i,\ i=0,1\bigg \}. \end{aligned} \end{aligned}$$

We refer to [2] for a survey on the Kantorovich–Wasserstein distance and related topics.

### Equivalent Formulations of the Hellinger–Kantorovich Distance

The Hellinger–Kantorovich distance was introduced in [26, 27] and independently in [21] and [10, 11]. It is a generalization of the Kantorovich–Wasserstein distance to arbitrary non-negative and finite measures by taking creation and annihilation of mass into account. Indeed, the latter can be associated with a different notion of distance, namely the Hellinger–Kakutani distance, see [19] and [33]. In this sense, the Hellinger–Kantorovich distance should be viewed as an infimal convolution of the Kantorovich–Wasserstein and the Hellinger–Kakutani distance, cf. [27, Rem. 8.19].

In [27], five different equivalent formulations of the Hellinger–Kantorovich distance are given: (i) the dynamical formulation, (ii) the cone space formulation, (iii) the optimal entropy-transport problem, (iv) the dual formulation in terms of Hellinger–Kantorovich potentials, and (v) the formulation using Hamilton–Jacobi equations. We will present and briefly discuss each of them below, as all are useful for our analysis of geodesic convexity.

In the follows, we consider the Hellinger–Kantorovich distance for measures on the domain $\mathbb {R}^d$. However, it is easy to see that all arguments also work in the case of a closed and convex domain $\Omega \subset \mathbb {R}^d$. In particular, the latter is a complete, geodesic space.

#### Dynamic Approach

A first approach to the Hellinger–Kantorovich distance is related to the dynamic formulation, which naturally depends on two positive parameters $\alpha ,\beta >0$; these control the relative strength of the Kantorovich–Wasserstein part and of the Hellinger-Kakutani part (see [27, Section 8.5]).

##### Definition 2.1

(*The dynamic formulation*) For every $\mu _0,\mu _1\in \mathcal {M}({\mathbb {R}^{d}})$ we set

2.3 $$\begin{aligned} \begin{aligned} \textsf{H}\!\!\textsf{K}_{\alpha ,\beta }(\mu _0,\mu _1)^2= \min \bigg \{&\int _{0}^1\!\! \int _{\mathbb {R}^{d}}\big (\alpha \, |\Upsilon ( t,x)|^2 {+}\beta \xi (t,x)^2\big ) \;\!\textrm{d}\mu _t(x)\;\!\textrm{d}t\,\Big |\, \\&\mu \in \textrm{C}([0,1];\mathcal {M}(\mathbb {R}^d)), \ \mu _{t=i}=\mu _i,\ \text {(gCE) holds} \bigg \}, \end{aligned} \end{aligned}$$

where the generalized continuity equation for the Borel vector and scalar fields $\Upsilon : (0,1)\times \mathbb {R}^d\rightarrow \mathbb {R}^d$ and $\xi :(0,1)\times \mathbb {R}^d\rightarrow \mathbb {R}$ reads as

$$\begin{aligned} \text {(gCE) } \qquad \frac{\partial }{\partial t} \mu +\alpha \mathop {\textrm{div}}(\mu \;\! \Upsilon ) =\beta \, \xi \mu \quad \text {in } {\mathcal {D}}'( (0,1)\times {\mathbb {R}^{d}}). \end{aligned}$$

Notice that (2.3) yields in particular that $\mu \;\! \Upsilon$ and $\xi \mu$ are (vector and scalar) measures with finite total mass, so that the canonical formulation of (gCE) in ${\mathcal {D}}'( (0,1)\times {\mathbb {R}^{d}})$ makes sense. For optimal solutions one has $\Upsilon (t,x)=\nabla \xi (t,x)$ and the dual potential solves the generalized Hamilton–Jacobi equation

2.4 $$\begin{aligned} \partial _t\xi + \frac{\alpha }{2} |\nabla \xi |^2 +\frac{\beta }{2} \xi ^2=0 \end{aligned}$$

in a suitable sense [27, Theorem 8.20].

A simple rescaling technique shows that it is sufficient to restrict ourselves to a specific choice of the parameters $\alpha$ and $\beta$. In fact, it is easy to see that for every $\theta >0$ we have

$$\begin{aligned} \textsf{H}\!\!\textsf{K}_{\alpha ,\beta }(\mu _0,\mu _1)^2 =\theta \textsf{H}\!\!\textsf{K}_{\theta \alpha ,\theta \beta }(\mu _0,\mu _1)^2. \end{aligned}$$

Moreover, if $\lambda >0$ and we consider the spatial dilation $H:x\mapsto \lambda x$ in ${\mathbb {R}^{d}}$, we find

$$\begin{aligned} \textsf{H}\!\!\textsf{K}_{\alpha ,\beta }(\mu _0,\mu _1)^2= \textsf{H}\!\!\textsf{K}_{\alpha /\lambda ^2,\beta }(H_\sharp \mu _0,H_\sharp \mu _1)^2. \end{aligned}$$

Choosing $\lambda :=\sqrt{4\alpha /\beta }$, $\theta = 4/\beta$, and setting $\textsf{H}\!\!\textsf{K}:=\textsf{H}\!\!\textsf{K}_{1,4}$ we get

$$\begin{aligned} \textsf{H}\!\!\textsf{K}_{\alpha ,\beta }(\mu _0,\mu _1)^2= \frac{4}{\beta }\textsf{H}\!\!\textsf{K}_{4\alpha /\beta ,4}(\mu _0,\mu _1)^2= \frac{4}{\beta }\textsf{H}\!\!\textsf{K}(H_\sharp \mu _0,H_\sharp \mu _1)^2. \end{aligned}$$

Therefore, in order to keep simpler notation, in the remaining paper we will mainly consider the case $\alpha =1$ and $\beta =4$.

#### Cone Space Formulation

There is a second characterization that connects $\textsf{H}\!\!\textsf{K}$ with the classic Kantorovich–Wasserstein distance on the extended cone $\mathfrak {C}:=({\mathbb {R}^{d}}\times [0,\infty [)/\!\sim$, where $\sim$ is the equivalence relation which identifies all the points (*x*, 0) with the vertex $\mathfrak {o}$ of $\mathfrak {C}$. More precisely, we write $(x_0,r_0)\sim (x_1,r_1)$ if and only if $x_0=x_1$ and $r_0 = r_1$ or $r_0=r_1=0$ and introduce the notation [*x*, *r*] to denote the equivalence class associated with $(x,r)\in {\mathbb {R}^{d}}\times [0,\infty [$. The cone $\mathfrak {C}$ is a complete metric space endowed with the cone distances

2.5 $$\begin{aligned} \textsf{d}_{a,\mathfrak {C}}(\mathfrak {z}_0,\mathfrak {z}_1)^2:= r_0^2+r_1^2-2r_0r_1 \cos _{a}\!{( |x_1{-}x_0|)}, \quad \mathfrak {z}_i=[x_i,r_i],\ a\in (0,\pi ],\nonumber \\ \end{aligned}$$

see e.g. [4, Sect. 3.6.2], where we use the abbreviation $\cos _a(r):=\cos \big (\!\min \{a,r\}\big )$. Notice that the projection map $(x,r)\mapsto [x,r]$ is bijective from $\mathbb {R}^d\times (0,\infty )$ to $\mathfrak {C}_{\varvec{*}}:=\mathfrak {C}\setminus \{\mathfrak {o}\}$; we will denote by $(\textsf{x},\textsf{r})$ its inverse, which we extend to $\mathfrak {o}$ by setting $\textsf{x}(\mathfrak {o})=0,\ \textsf{r}(\mathfrak {o})=0$.

The most natural choice of the parameter *a* in (2.5) is $a:=\pi$: in this case the cone $(\mathfrak {C},\textsf{d}_{\pi ,\mathfrak {C}})$ is a geodesic space, i.e., given $\mathfrak {z}_i=[x_i,r_i]$, $i=0,1$, there exists a curve $\mathfrak {z}_t=[x_t,r_t]=\textrm{geo}_{t}\big (\mathfrak {z}_0,\mathfrak {z}_1\big )$, $t \in [0,1]$, connecting $\mathfrak {z}_0$ to $\mathfrak {z}_1$ and satisfying

2.6 $$\begin{aligned} \forall \,0\leqq s,t\leqq 1:\quad \textsf{d}_{\pi ,\mathfrak {C}}(\mathfrak {z}_s,\mathfrak {z}_t)=|t{-}s|\,\textsf{d}_{\pi ,\mathfrak {C}}(\mathfrak {z}_0,\mathfrak {z}_1). \end{aligned}$$

If one of the two points coincides with $\mathfrak {o}$, e.g. for $\mathfrak {z}_0=\mathfrak {o},$ it is immediate to check that $\mathfrak {z}_t=[x_1,tr_1]$. If $r_0,r_1>0$ and $|x_1 {-}x_0|< \pi /2$ then the unique geodesic curve reads (recall the convention in (1.6))

2.7 $$\begin{aligned} \begin{aligned}&r_t:=r_0\Big ((1{+}t u)^2+t^2|{\textbf{v}}|^2\Big )^{1/2},\quad x_t:=x_0+ \textbf{arctan}\big (\frac{t{\textbf{v}}}{1{+}tu}\Big ),\\&\text {where } u=\frac{r_1}{r_0}\cos (|x_1{-}x_0|)-1 \text { and } {\textbf{v}}:=\frac{r_1}{r_0}\,{\textbf {sin}}(x_1{-}x_0). \end{aligned} \end{aligned}$$

For example, if we operate the same construction starting from the one-dimensional set $\Omega =[0,L]\subset \mathbb {R}$ with $0<L \leqq \pi$ we can isometrically identify the cone space over $\Omega$ with the two-dimensional sector $\Sigma _\Omega = \big \{\, y=(r\,\cos x,\ r\,\sin x)\in \mathbb {R}^2 \, \big | \, r\geqq 0,~x\in [0,L] \,\big \}$ endowed with the Euclidean distance. For $L\in {]\pi ,2\pi [}$ the identification with the sector still holds, but the sector $\Sigma _\Omega$ is no more convex and for $x_0,x_1\in \Omega$ with $|x_0{-}x_1|\geqq \pi$ the cone distance corresponds to the geodesic distance on the sector $\Sigma _\Omega$, i.e. the length of the shortest path in $\Sigma _\Omega$ connecting two points.

On the one hand, we can define a homogeneous projection $\mathfrak {h}: \mathcal {M}_2(\mathfrak {C})\rightarrow \mathcal {M}({\mathbb {R}^{d}})$, via

2.8 $$\begin{aligned} \mathfrak {h}\lambda := \textsf{x}_\sharp (\textsf{r}^2\lambda )= \int _{r=0}^\infty r^2\,\lambda (\cdot ,\textrm{d}r), \end{aligned}$$

i.e. for every $\lambda \in \mathcal {M}_2(\mathfrak {C})$ and $\zeta \in \textrm{C}_b({\mathbb {R}^{d}})$ we have

$$\begin{aligned} \int _{\mathbb {R}^{d}}\zeta (x)\;\!\textrm{d}(\mathfrak {h}\lambda )= \int _\mathfrak {C}r^2 \zeta (x)\;\!\textrm{d}\lambda (x,r). \end{aligned}$$

On the other hand, measures in $\mathcal {M}({\mathbb {R}^{d}})$ can be “lifted” to measures in $\mathcal {M}_2(\mathfrak {C})$, e.g. by considering the measure $\mu \otimes \delta _{1}$ for $\mu \in \mathcal {M}({\mathbb {R}^{d}})$. More generally, for every Borel map $r:\mathbb {R}^d\rightarrow \left]0,\infty \right[$ and constant $m_0\geqq 0$, the measure $\lambda = m_0 \delta _{\mathfrak {o}} + \mu \otimes \frac{1}{r(\cdot )^2} \delta _{r(\cdot )}$ gives $\mathfrak {h}\lambda = \mu$.

Now, the cone space formulation of the Hellinger–Kantorovich distance between two measures $\mu _0$, $\mu _1\in \mathcal {M}({\mathbb {R}^{d}})$ is given as follows, (see [26, Sec. 3]);

##### Theorem 2.2

(Optimal transport formulation on the cone) For $\mu _0,\mu _1\in \mathcal {M}(\mathbb {R}^d)$ we have

2.9a $$\begin{aligned} \textsf{H}\!\!\textsf{K}(\mu _0,\mu _1)^2&= \min \Big \{\textsf{W}_{\pi ,\mathfrak {C}}(\lambda _0,\lambda _1)^2\,\Big |\,\lambda _i\in \mathcal P_2(\mathfrak {C}),\ \mathfrak {h}\lambda _i = \mu _i\Big \} \end{aligned}$$

2.9b $$\begin{aligned}&=\min \Big \{\int \int _{\mathfrak {C}\times \mathfrak {C}} \textsf{d}_{\pi ,\mathfrak {C}}(z_0,z_1)^2\textrm{d}{\varvec{\lambda }}(z_0,z_1)\,\Big |\, \mathfrak {h}_i{\varvec{\lambda }}=\mu _i\Big \}, \end{aligned}$$

where $\mathfrak {h}$ is defined in (2.8) and $\mathfrak {h}_i{\varvec{\lambda }}:=\mathfrak {h}(\pi ^i_\sharp {\varvec{\lambda }})$ for ${\varvec{\lambda }}\in \mathcal {M}_2(\mathfrak {C}{\times }\mathfrak {C})$ and $i=0,1$.

The cone space formulation is reminiscent of classical optimal transport problems. Here, however, the marginals $\lambda _i$ of the transport plan ${\varvec{\lambda }}\in \mathcal {M}(\mathfrak {C}\times \mathfrak {C})$ are not fixed, and it is part of the problem to find an optimal pair of measures $\lambda _i$ satisfying the constraints $\mathfrak {h}\lambda _i=\mu _i$ and having minimal Kantorovich–Wasserstein distance on the cone space.

##### Remark 2.3

(**Hellinger–Kantorovich space as cone**) In [22] it is shown that the space $(\mathcal {M}(\mathbb {R}^d);\textsf{H}\!\!\textsf{K})$ can be understood as a cone space over the geodesic space $(\mathcal {P}(\mathbb {R}^d),\mathsf S\!\textsf{H}\!\!\textsf{K})$ where the *spherical Hellinger–Kantorovich distance* in $\mathcal {P}(\mathbb {R}^d)$ reads $\mathsf S\!\textsf{H}\!\!\textsf{K}(\nu _0,\nu _1):= \arccos \big ( 1-\frac{1}{2} \textsf{H}\!\!\textsf{K}(\nu _0,\nu _1)^2\big )$. It would be interesting to analyze geodesic convexity properties of functionals ${\mathscr {E}}$ as in (1.20) on this space; see [23] for a first result.

The cone space formulation in (2.9) reveals many interesting geometric properties of the Hellinger–Kantorovich distance, e.g. Hellinger–Kantorovich geodesics are directly connected to geodesic curves in the cone space $\mathfrak {C}$, see below. Moreover, it can be deduced that a sharp threshold exists, which distinguishes between transport of mass and pure growth (i.e. creation or destruction) of mass.

##### Remark 2.4

The link between the dynamical formulation in (2.3) and the cone-space formulation in (2.9) of the Hellinger–Kantorovich distance can be best seen from a Lagrangian point of view. Let $\textsf{Lag}_{\alpha ,\beta }( X, r;V,\varrho )= \frac{r^2}{\alpha }|V|^2+ \frac{4}{\beta } \varrho ^2$ denote the rescaled Lagrangian in the definition of the dynamical functional (2.3) corresponding to a curve of the form $\mu _t:=r^2(t)\delta _{X(t)}$ and consider for fixed $r_0$, $r_1>0$ and $x_0$, $x_1\in {\mathbb {R}^{d}}$ the minimization problem

$$\begin{aligned} M_{\alpha ,\beta } (x_0,r_0;x_1,r_1):= \min \Big \{ \!\int _0^1 \!\!\textsf{Lag}_{\alpha ,\beta }\big (X(s),r(s);\dot{X}(s),\dot{r}(s)\big )\;\!\textrm{d}s\, \Big |\\ (X,r)\in \textrm{C}^1\big ([0,1];{\mathbb {R}^{d}}\times \mathbb {R}_+\big ), X(i)=x_i,~r(i)=r_i,\Big \}. \end{aligned}$$

It is not hard to check [26, Sec. 3.1] that we obtain for $(\alpha ,\beta )=(1,4)$ the explicit formula

$$\begin{aligned} \textsf{H}\!\!\textsf{K}(\mu _0,\mu _1)^2=M_{1,4} (x_0,r_0;x_1,r_1) = \textsf{d}_{\pi ,\mathfrak {C}}([x_0,r_0],[x_1,r_1])^2, \end{aligned}$$

which is the Hellinger–Kantorovich distance of the two Dirac measures $\mu _0=r_0^2\delta _{x_0}$ and $\mu _1 = r_1^2\delta _{x_1}$ in the case that $|x_0{-}x_1|<\pi /2$.

When $|x_0{-}x_1|\geqq \pi /2$, one can always connect $\mu _0$ to $\mu _1$ by the curve $\mu _t:=\big ((1{-}t)r_0\big )^2\delta _{x_0}+t^2r_1^2\delta _{x_1}$ (whose support is no longer concentrated on a single point) obtaining

$$\begin{aligned} \textsf{H}\!\!\textsf{K}(\mu _0,\mu _1)^2=2=\textsf{d}_{\pi /2,\mathfrak {C}}([x_0,r_0],[x_1,r_1])^2, \end{aligned}$$

and showing the role of the threshold $\pi /2$ instead of $\pi$ in the computation of $\textsf{H}\!\!\textsf{K}$.

The explicit computation of the previous remark is in fact a particular case of a general result [27, Lem. 7.9+7.19].

##### Theorem 2.5

(Effective $\pi /2$-threshold in the cone distance) Let $\mu _0,\mu _1\in \mathcal {M}({\mathbb {R}^{d}})$, if ${\varvec{\lambda }}\in \mathcal {M}_2(\mathfrak {C}{\times }\mathfrak {C})$ is an optimal plan for the cone-space formulation (2.9) then  is still optimal and

2.10 $$\begin{aligned} {\varvec{\lambda }}\Big (\big \{([x_0,r_0],[x_1,r_1])\in \mathfrak {C}\times \mathfrak {C}\,\Big | \, r_0r_1>0 \text { and } |x_0{-}x_1|>\frac{\pi }{2}\big \}\Big )=0, \end{aligned}$$

so that

2.11a $$\begin{aligned} \textsf{H}\!\!\textsf{K}(\mu _0,\mu _1)^2&= \min \Big \{\textsf{W}_{\pi /2,\mathfrak {C}}(\lambda _0,\lambda _1)^2\,\Big |\,\lambda _i\in \mathcal P_2(\mathfrak {C}),\ \mathfrak {h}\lambda _i = \mu _i\Big \} \end{aligned}$$

2.11b $$\begin{aligned}&=\min \Big \{\int \int _{\mathfrak {C}\times \mathfrak {C}} \textsf{d}_{\pi /2,\mathfrak {C}}(z_0,z_1)^2\textrm{d}{\varvec{\lambda }}(z_0,z_1)\,\Big |\, \mathfrak {h}_i{\varvec{\lambda }}=\mu _i\Big \}. \end{aligned}$$

Moreover, setting for $i=0,\tau$

**Fig. 1:**
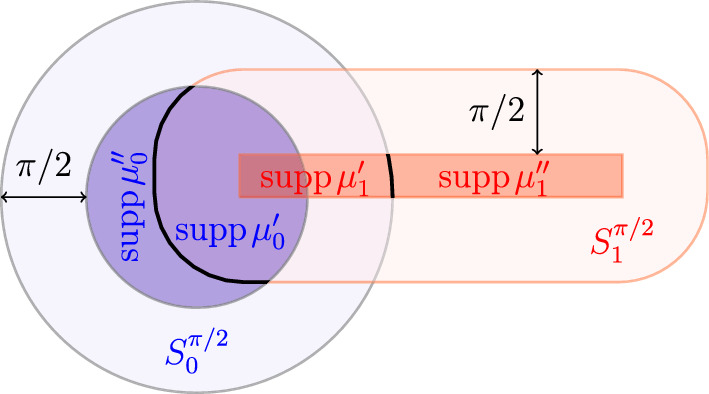
The decomposition of the closed supports $S_i={\text {supp}}\mu _i$ of the measures $\mu _i=\mu '_i+\mu ''_i$ as given in (2.13) with cut-off at $\pi /2$. The open sets $S^{\pi /2}_0$ and $S^{\pi /2}_1$ denote the $\pi /2$-neighborhoods of the supports $S_1$ and $S_0$, respectively, and ,  are the corresponding restrictions of the measures $\mu _i$

2.12 $$\begin{aligned} \begin{aligned}&S_i:={\text {supp}}(\mu _i),\quad S^{\pi /2}_i:= \big \{\, x\in { {\mathbb {R}^{d}}} \, \big | \, \textrm{dist}(x,S_i) <{\pi /2} \,\big \} , \\&S_i':=S_i \cap S^{\pi /2}_{1-i}, \quad \text { and } S_i'':= S_i \setminus S^{\pi /2}_{1-i}, \end{aligned} \end{aligned}$$

(see Fig. 1) with the related decomposition2.13then we have that

2.14a $$\begin{aligned}{} & {} \textsf{H}\!\!\textsf{K}(\mu _0,\mu _\tau )^2= \textsf{H}\!\!\textsf{K}(\mu _0',\mu _\tau ')^2 + \textsf{H}\!\!\textsf{K}(\mu _0'',\mu _\tau '')^2, \end{aligned}$$

2.14b $$\begin{aligned}{} & {} \textsf{H}\!\!\textsf{K}(\mu _0'',\mu _\tau '')^2=\mu _0''({\mathbb {R}^{d}})+\mu _\tau ''({\mathbb {R}^{d}})= \mu _0({\mathbb {R}^{d}}\setminus S_0')+ \mu _\tau ({\mathbb {R}^{d}}\setminus S_\tau ').\qquad \quad \end{aligned}$$

Note that (2.14a) shows that the decomposition in (2.13) is extremal with respect to the subadditivity property in Lemma 7.8 of [27], and (2.14b) shows that the computation of $\textsf{H}\!\!\textsf{K}^2$ between $\mu _0''$ and $\mu _1''$ is trivial, so that no information is lost if one restricts the evaluation of  and . Motivated by the above properties, we introduce the following definition of reduced pairs, which will play a crucial role in our analysis of geodesic curves;

##### Definition 2.6

(*Reduced pairs*) A pair $(\mu _0,\mu _1)\in \mathcal {M}({\mathbb {R}^{d}})^2$ is called *reduced* (resp. *strongly reduced*) if $\mu _i(S_i'')=0$, i.e. $\mu _i=\mu _i'$ for $i=0$ and 1 (resp. if $S_i\subset S^{\pi /2}_{1-i}$).

By definition, the sets $S_i=\textrm{supp}(\mu _i)$ are closed and $S^{\pi /2}_i$ are open, so that $S''_i=S_i\setminus S^{\pi /2}_{1-i}$ is closed as well, but $S'_i=S_i\cap S^{\pi /2}_{1-i}$ may be neither closed nor open. In the strongly reduced case the condition $S_i\subset S^{\pi /2}_{1-i}$ means that, at least locally, the closed set $S_i$ has a positive distance to the boundary of the open set $S^{\pi /2}_{1-i}$.

Notice that for every $(\mu _0,\mu _1)\in \mathcal {M}({\mathbb {R}^{d}})^2$ the corresponding pair $(\mu _0',\mu _1')$ defined according to (2.12)–(2.13) is reduced by construction. In fact, if $x\in S_0'$ then there exists $y\in {\text {supp}}(\mu _1)$ with $|x{-}y|<\pi /2$: clearly $y\in S_1'$ so that $\textrm{dist}(x,{\text {supp}}(\mu _1'))\leqq \textrm{dist}(x,S_1') < \pi /2$.

#### Transport-growth Maps

It is useful to express (2.11b) in an equivalent way, which extends the notion of transport maps to the unbalanced case. This relies on special families of plans in ${\varvec{\lambda }}\in \mathcal {M}_2(\mathfrak {C}^2)$ with $\mathfrak {h}_i{\varvec{\lambda }}= \mu _i$ generated by transport-growth systems.

##### Definition 2.7

(*Transport-growth maps*) Let $\nu \in \mathcal {M}(Y)$, where *Y* is some Polish space. A transport-growth map is a $\nu$-measurable map $(\varvec{T},q):Y\rightarrow X\times [0,\infty )$ with $q\in L^2(Y,\nu )$. It acts on $\nu$ according to this rule:

2.15 $$\begin{aligned} (\varvec{T},q)_\star \nu :=\varvec{T}_\sharp (q^2\nu ) =\mathfrak {h}((\varvec{T},q)_\sharp \nu ). \end{aligned}$$

there the last identity involves the obvious generalization of the definition (2.8) of homogeneous projection $\mathfrak {h}$ from $\mathcal {M}_2(X\times [0,\infty ))$ to $\mathcal {M}(X)$.

We notice that transport-growth maps obey the composition rule

2.16 $$\begin{aligned} (\varvec{T}_2,q_2)_\star (\varvec{T}_1,q_1)_\star \nu =(\varvec{T},q)_\star \nu \quad \text {where}\quad \varvec{T}:=\varvec{T}_2\circ \varvec{T}_1,\ q:=(q_2\circ \varvec{T}_1)q_1.\nonumber \\ \end{aligned}$$

Transport-growth maps provide useful upper bounds for the $\textsf{H}\!\!\textsf{K}$ metric, playing a similar role of transport maps for the Kantorovich–Wasserstein distance. In fact, for every choice of maps $(\varvec{T}_i, q_i):Y\rightarrow \mathbb {R}^d\times [0,\infty )$, $i=0,1$, associated with the measure $\nu \in \mathcal {M}(Y),$ we have

2.17 $$\begin{aligned} \textsf{H}\!\!\textsf{K}^2(\mu _0,\mu _1)\leqq \int _Y \Big (q_0^2+q_1^2-2q_0q_1 \cos _{\pi /2}\!{(|\varvec{T}_0{-}\varvec{T}_1|)} \Big )\;\!\textrm{d}\nu \quad \mu _i:=(\varvec{T}_i,q_i)_\star \nu .\nonumber \\ \end{aligned}$$

In order to show (2.17) it is sufficient to check that the measure ${\varvec{\lambda }}\in \mathcal {M}_2(\mathfrak {C}^2)$ defined by

$$\begin{aligned} {\varvec{\lambda }}:=(\varvec{T}_0,q_0;\varvec{T}_1,q_1)_\sharp \nu ,\quad \text { satisfies}\quad \mathfrak {h}_i{\varvec{\lambda }}=\mu _i \end{aligned}$$

so that (2.17) follows from (2.11b) and the identity

2.18 $$\begin{aligned} \int _{\mathfrak {C}^2} \textsf{d}_{\pi /2,\mathfrak {C}}(z_0,z_1)^2 \;\!\textrm{d}{\varvec{\lambda }}= \int _Y \Big (q_0^2+q_1^2-2q_0q_1 \cos _{\pi /2}\!{(|\varvec{T}_0{-}\varvec{T}_1|)}\Big ) \;\!\textrm{d}\nu . \qquad \end{aligned}$$

On the other hand, choosing $Y=\mathfrak {C}\times \mathfrak {C}$ and an optimal plan $\nu ={\varvec{\lambda }}\in \mathcal {M}_2(\mathfrak {C}\times \mathfrak {C})$ for (2.11b)

and setting $\varvec{T}_i([x_0,r_0],[x_1,r_1]):=x_i$ and $q_i([x_0,r_0],[x_1,r_1])=r_i$, we immediately find

2.19 $$\begin{aligned} \textsf{H}\!\!\textsf{K}^2(\mu _0,\mu _1)= \int _{\mathfrak {C}\times \mathfrak {C}} \Big (q_0^2+q_1^2-2q_0q_1 \cos _{\pi /2}\!{(|\varvec{T}_0{-}\varvec{T}_1|)} \Big )\;\!\textrm{d}{\varvec{\lambda }},\quad \mu _i:=(\varvec{T}_i,q_i)_\star {\varvec{\lambda }},\nonumber \\ \end{aligned}$$

and therefore equality holds in (2.17).

##### Corollary 2.8

($\textsf{H}\!\!\textsf{K}$ via transport-growth maps) For every $\mu _0,\mu _1\in \mathcal {M}(\mathbb {R}^d)$ we have

2.20 $$\begin{aligned} \textsf{H}\!\!\textsf{K}^2(\mu _0,\mu _1)= & {} \min \Big \{\int _{\mathfrak {C}\times \mathfrak {C}} \Big (q_0^2+q_1^2 -2q_0q_1 \cos _{\pi /2}\!{(|\varvec{T}_0{-}\varvec{T}_1|)}\Big ) \;\!\textrm{d}{\varvec{\lambda }}\Big |\, {\varvec{\lambda }}\in \mathcal {M}( Y), \nonumber \\{} & {} \quad Y\text {Polish,\ }(\varvec{T}_i,q_i):Y \rightarrow \mathbb {R}^d\times [0,+\infty ),\ \mu _i:=(\varvec{T}_i,q_i)_\star {\varvec{\lambda }}\Big \};\nonumber \\ \end{aligned}$$

moreover, it is not restrictive to choose $Y=\mathfrak {C}\times \mathfrak {C}$ in (2.20).

Inspired by the so-called Monge formulation of Optimal Transport, it is natural to look for similar improvement of (2.20), when $Y=\mathbb {R}^d$, $\nu =\mu _0$, $\varvec{T}_0(x_0)=x_0$ is the identity map, and $q(x_0)\equiv 1$.

##### Problem 2.9

(Monge formulation of $\textsf{H}\!\!\textsf{K}$ problem) Given $\mu _0,\mu _1\in \mathcal {M}({\mathbb {R}^{d}})$ such that $\mu _1=\mu _1'$, $\mu _1''=0$ (recall (2.12) and (2.13)), find an optimal transport-growth pair $(\varvec{T},q):\mathbb {R}^d\rightarrow \mathbb {R}^d\times [0,\infty )$ minimizing the cost

2.21 $$\begin{aligned} {\mathcal {C}}(\varvec{T},q;\mu _0):=\int _{{\mathbb {R}^{d}}} \Big (1+q^2(x)-2q(x) \cos _{{\pi /2}}\!{(|\varvec{T}(x){-}x|)}\Big )\;\!\textrm{d}\mu _0(x)\qquad \end{aligned}$$

among all the transport-growth maps satisfying $(\varvec{T},q)_\star \mu _0=\mu _1$

By (2.17) we have the bound

2.22 $$\begin{aligned} \begin{aligned} \textsf{H}\!\!\textsf{K}(\mu _0,\mu _1)^2 \leqq \inf \Big \{{\mathcal {C}}(\varvec{T},q;\mu _0)\Big | (\varvec{T},q)_\star \mu _0=\mu _1\Big \}. \end{aligned} \end{aligned}$$

When $\mu _0 \ll \mathcal {L}^{d}$ and the support of $\mu _1$ is contained in the closed neighborhood of radius $\pi /2$ of the support of $\mu _0$, the results of the next section (cf. Corollary 3.5), which are a consequence of the optimality conditions in Theorem 2.14, show that the minimum of Problem 2.9 is attained and realizes the equality in (2.22).

#### Entropy-Transport Problem

A third point of view, typical of optimal transport problems, characterizes the Hellinger–Kantorovich distance via the static Logarithmic Entropy Transport (LET) variational formulation.

We define the logarithmic entropy density $F:{}[0,\infty [\rightarrow [0,\infty [{}$ via

$$\begin{aligned} F(s):= s\log s - s + 1\quad \text {for}\, s>0\quad \text {and} \quad F(0):=1, \end{aligned}$$

and the cost function $\textrm{L}_1:\mathbb {R}^d\rightarrow [0,\infty ]$ via

2.23 $$\begin{aligned} \textrm{L}_1(x):= \frac{1}{2}\ell (|x|),\quad \ell (r):= {\left\{ \begin{array}{ll} \displaystyle -\log (\cos ^2(r))=\log \big (1{+}\tan ^2(r)\big )&{}\text {for }r <\pi /2,\\ +\infty &{}\text {otherwise.} \end{array}\right. }\nonumber \\ \end{aligned}$$

For given $\mu _0,\mu _1 \in \mathcal {M}({\mathbb {R}^{d}})$ the entropy-transport functional $\mathscr {E}\hspace{-3.0pt}\mathcal {T}(\,\cdot \,;\mu _0,\mu _1): \mathcal {M}({\mathbb {R}^{d}}\times {\mathbb {R}^{d}})\rightarrow [0,\infty ]$ reads as

2.24 $$\begin{aligned} \mathscr {E}\hspace{-3.0pt}\mathcal {T}({\varvec{\eta }};\mu _0,\mu _1):= \int _{\mathbb {R}^{d}}F(\sigma _0)\;\!\textrm{d}\mu _0+ \int _{\mathbb {R}^{d}}F(\sigma _1)\;\!\textrm{d}\mu _1+ \iint _{{\mathbb {R}^{d}}\times {\mathbb {R}^{d}}}2\textrm{L}_1(x_0{-}x_1)\;\!\textrm{d}{\varvec{\eta }},\nonumber \\ \end{aligned}$$

with $(\pi _i)_\sharp {\varvec{\eta }}=\sigma _i\mu _i\ll \mu _i$. As usual, we set $\mathscr {E}\hspace{-3.0pt}\mathcal {T}({\varvec{\eta }};\mu _0,\mu _1):=+\infty$ if one of the marginals $(\pi _i)_\sharp {\varvec{\eta }}$ of ${\varvec{\eta }}$ is not absolutely continuous with respect to $\mu _i$. With this definition, the equivalent formulation of the Hellinger–Kantorovich distance as entropy-transport problem reads as follows:

##### Theorem 2.10

(LET formulation) For every $\mu _0,\mu _1\in \mathcal {M}({\mathbb {R}^{d}})$ we have

2.25 $$\begin{aligned} \textsf{H}\!\!\textsf{K}(\mu _0,\mu _1)^2=\min \Big \{\mathscr {E}\hspace{-3.0pt}\mathcal {T}({\varvec{\eta }};\mu _0,\mu _1)\,\big |\, {\varvec{\eta }}\in \mathcal {M}({\mathbb {R}^{d}}\times {\mathbb {R}^{d}}) \Big \}. \end{aligned}$$

Moreover, recalling the decomposition (2.12)–(2.13),

1. the pairs $(\mu _0,\mu _\tau )$ and $(\mu '_0,\mu '_\tau )$ share the same optimal plans ${\varvec{\eta }}$
2. if we set $g_0(x_0):=([x_0,1],\mathfrak {o})$ and $g_1(x_1):=(\mathfrak {o},[x_1,1])$, every optimal plan ${\varvec{\eta }}\in \mathcal {M}({\mathbb {R}^{d}}\times {\mathbb {R}^{d}})$ for the entropy-transport formulation in (2.25) induces optimal plans ${\varvec{\beta }}$ (resp. ${\varvec{\beta }}'$) in $\mathcal {M}( \mathfrak {C}\times \mathfrak {C})$ for the pair $(\mu _0,\mu _1)$ (resp. the reduced pair $(\mu _0',\mu _1')$) via
  2.26 $$\begin{aligned} {\varvec{\beta }}':=(x_0,\sigma _0^{-1/2};x_1,\sigma _1^{-1/2})_\sharp {\varvec{\eta }},\quad {\varvec{\beta }}:={\varvec{\beta }}'+(g_0)_\sharp \, \mu ''_0 +(g_1)_\sharp \, \mu _1''. \end{aligned}$$

An optimal transport plan ${\varvec{\eta }}$, which always exists, gives the effective transport of mass. Note, in particular, that the finiteness of $\mathscr {E}\hspace{-3.0pt}\mathcal {T}$ only requires $(\pi _i)_\sharp {\varvec{\eta }}=\eta _i\ll \mu _i$ (which is considerably weaker than the usual transport constraint $(\pi _i)_\sharp {\varvec{\eta }}=\mu _i$) and the cost of a deviation of $\eta _i$ from $\mu _i$ is given by the entropy functionals associated with *F*. Moreover, the cost function $\ell$ is finite in the case $|x_0{-}x_1|<\pi /2$, which highlights the sharp threshold between transport and pure creation/destruction. Notice that we could equivalently use the truncated function $\cos ^2_{{\pi /2}}\!{\left( r\right) }=\cos ^2(\min \{r,{\pi /2}\})$ instead of $\cos ^2(r)$ in (2.23). As we have already seen, the function $r \mapsto \cos ^2_{{\pi /2}}\!{\left( r\right) }$ plays an important role in many formulae.

In general, optimal entropy-transport plans ${\varvec{\eta }}\in \mathcal {M}({\mathbb {R}^{d}}\times {\mathbb {R}^{d}})$ are not unique. However, due to the strict convexity of *F*, their marginals $\eta _i$ are unique so that the non-uniqueness of the plan ${\varvec{\eta }}$ is solely a property of the optimal transport problem associated with the cost function $(x_0,x_1) \mapsto 2\textrm{L}_1(x_1{-}x_0)= \ell \big (|x_1{-}x_0|\big )$.

##### Remark 2.11

Besides (2.26), the connection between the cone-space formation and the logarithmic entropy-transport problem is given by the homogeneous marginal perspective function, namely

$$\begin{aligned} \textsf{d}_{\pi /2,\mathfrak {C}} ([x_0, r_0],[x_1,r_1])^2 =\inf \big \{ r_0^2 F(\tfrac{\theta }{r_0^2}) + r_1^2F\big (\tfrac{\theta }{r_1^2}\big ) +2\theta \textrm{L}_1(x_0{-}x_1) \,\big |\, \theta > 0\big \}, \end{aligned}$$

where $r_i^2$ plays the role of the reverse densities $1/\sigma _i$ and $\theta$ is a scaling parameter, see [27, Sec. 5].

We highlight that the logarithmic entropy-transport formulation (2.25) can be easily generalized by considering convex and lower semi-continuous functions $F_0$ and $F_1$ and cost functions $\ell$, see [27, Part I].

Applying the previous Theorem 2.10 we can refine formula (2.18) by providing an optimal pair of transport-growth maps solving (2.20) in the restricted set $Y=S_0\times S_1\subset {\mathbb {R}^{d}}\times {\mathbb {R}^{d}}$. Indeed, we can choose arbitrary points ${\bar{x}}_i\in S_i$ and

2.27 $$\begin{aligned} {\varvec{\nu }}:= & {} {\varvec{\eta }}+ \mu _0'' {\otimes }\delta _{{\bar{x}}_1}+\delta _{{\bar{x}}_0}{\otimes }\mu _1'', \nonumber \\ \varvec{T}_i(x_0,x_1):= & {} x_i,\quad q_i(x_0,x_1):= {\left\{ \begin{array}{ll} \sigma _i^{-1/2}(x_i)&{}\text {if }(x_0,x_1)\in S_0'\times S_1',\\ 1&{}\text {if }(x_0,x_1)\in (S_0\times S_1)\setminus (S_0'\times S_1'), \end{array}\right. }\nonumber \\ \end{aligned}$$

which satisfies

2.28 $$\begin{aligned} (\varvec{T}_i,q_i)_\star {\varvec{\nu }}=\mu _i,\quad \textsf{H}\!\!\textsf{K}^2(\mu _0,\mu _1)= \int _Y \Big (q_0^2+q_1^2-2q_0q_1\cos _{{\pi /2}}\!{(|\varvec{T}_0{-}\varvec{T}_1|)}\Big )\;\!\textrm{d}{\varvec{\nu }}.\nonumber \\ \end{aligned}$$

#### Dual Formulation with Hellinger–Kantorovich Potentials

In analogy to the Kantorovich–Wasserstein distance, we can give a dual formulation in terms of Hellinger–Kantorovich potentials. We slightly modify the notation of [27], in order to be more consistent with the approach by the Hamilton–Jacobi equations (and the related Hopf–Lax solutions) of Section 4 and to deal with rescaled distances. As we will study segments of constant-speed geodesics $t\rightarrow \mu _t$ of length $\tau = t{-}s$ for $0\leqq s < t \leqq 1$, it will be convenient to introduce a scaling parameter $\tau >0$ that in certain parts will be replaced by 1, namely if we consider a whole geodesic. With this parameter, we set

2.29 $$\begin{aligned} F_\tau (s):=\frac{1}{2\tau } F(s),\quad \textrm{L}_\tau (x)=\frac{1}{2\tau } \ell (|x|), \quad \mathscr {E}\hspace{-3.0pt}\mathcal {T}_\tau ({\varvec{\eta }};\mu _0,\mu _\tau )=\frac{1}{2\tau }\mathscr {E}\hspace{-3.0pt}\mathcal {T}({\varvec{\eta }};\mu _0,\mu _\tau ),\nonumber \\ \end{aligned}$$

and the corresponding

2.30 $$\begin{aligned} \frac{1}{2\tau }\textsf{H}\!\!\textsf{K}^2(\mu _0,\mu _\tau )=\min \Big \{\mathscr {E}\hspace{-3.0pt}\mathcal {T}_\tau ({\varvec{\eta }};\mu _0,\mu _\tau ) \,\Big |\, {\varvec{\eta }}\in \mathcal {M}({\mathbb {R}^{d}}\times {\mathbb {R}^{d}}) \Big \}. \end{aligned}$$

It is clear that minimizers ${\varvec{\eta }}$ of (2.30) are independent of the coefficient $\frac{1}{2\tau }$ in front of $\textsf{H}\!\!\textsf{K}$ and coincide with solutions to (2.25) if $\mu _\tau =\mu _1$. The role of $\tau$ just affects the rescaling of the potentials $\varphi$ and $\xi$ we will introduce below.

We also introduce the Legendre transform of $F_\tau$

2.31 $$\begin{aligned}{} & {} \check{\textsf{G}}_\tau (\varphi ):= F_\tau ^* (\varphi ) = \sup _{s>0} \varphi s-F_\tau (s)= \frac{\textrm{e}^{2\tau \varphi }-1}{2\tau },\nonumber \\{} & {} \textsf{G}_\tau (\varphi ):=\frac{1-\textrm{e}^{-2\tau \varphi }}{2\tau }=-\check{\textsf{G}}_\tau (-\varphi ), \end{aligned}$$

extended to $[-\infty ,+\infty ]$ by

2.32 $$\begin{aligned} \textsf{G}_\tau (+\infty )=-\check{\textsf{G}}_\tau (-\infty )=\frac{1}{2\tau },\quad \textsf{G}_\tau (-\infty )=-\check{\textsf{G}}_\tau (+\infty )=+\infty , \end{aligned}$$

and their inverses

2.33 $$\begin{aligned} \check{\textsf{G}}^{-1}_\tau (\xi ):=\frac{1}{2\tau }\log (1{+}2\tau \xi ),\quad {\textsf{G}}^{-1}_\tau (\xi ):= -\frac{1}{2\tau }\log (1{-}2\tau \xi )=-\check{\textsf{G}}^{-1}_\tau (-\xi ),\nonumber \\ \end{aligned}$$

defined for $\xi \in [-\frac{1}{2\tau },+\infty ]$ and $\xi \in [-\infty ,\frac{1}{2\tau }]$ respectively, with the obvious convention induced by (2.32). With Theorem 6.3 in [27] (see also Section 4 therein), we have the equivalent characterization of $\textsf{H}\!\!\textsf{K}$ via the dual formulation

2.34a $$\begin{aligned} \frac{1}{2\tau } \textsf{H}\!\!\textsf{K}(\mu _0,\mu _\tau )^2&=\sup \Big \{\int _{\mathbb {R}^{d}}\textsf{G}_\tau (\varphi _\tau )\;\!\textrm{d}\mu _\tau -\int _{\mathbb {R}^{d}}\check{\textsf{G}}_\tau (\varphi _0) \;\!\textrm{d}\mu _0\,\Big | \, \nonumber \\&\hspace{5em}\varphi _0,\varphi _\tau \in \textrm{C}_b({\mathbb {R}^{d}}),~ \varphi _\tau (x_\tau ){-}\varphi _0(x_0)\leqq \textrm{L}_\tau (x_\tau {-}x_0)\Big \} \nonumber \\&= \sup \Big \{\int _{\mathbb {R}^{d}}\xi _\tau \;\!\textrm{d}\mu _\tau -\int _{\mathbb {R}^{d}}\xi _0\;\!\textrm{d}\mu _0\,\Big |\, \xi _i\in \textrm{C}_b({\mathbb {R}^{d}}),\nonumber \\&\qquad \qquad \sup _{\mathbb {R}^{d}}\xi _\tau <\frac{1}{2\tau },\ \inf _{\mathbb {R}^{d}}\xi _0>- \frac{1}{2\tau } \end{aligned}$$

2.34b $$\begin{aligned}&\qquad \qquad \big (1{-} 2\tau \xi _\tau (x_\tau )\big ) \big (1{+}2\tau \xi _0(x_0)\big ) \geqq \cos ^2_{{\pi /2}}\!{\left( |x_0{-}x_\tau |\right) } \Big \}. \end{aligned}$$

Note that the formulations in (2.34a) and (2.34b) are connected by the transformation $\xi _\tau = \textsf{G}_\tau (\varphi _\tau ),\ \xi _0=\check{\textsf{G}}_\tau (\varphi _0)$ and the last condition in (2.34b) is equivalent to

2.35 $$\begin{aligned} {\textsf{G}}^{-1}_\tau \big (\xi _\tau (x_\tau )\big ){-} \check{\textsf{G}}^{-1}_\tau \big (\xi _0(x_0)\big )\leqq \textrm{L}_\tau (x_\tau {-}x_0). \end{aligned}$$

It is not difficult to check that one can also consider Borel functions in (2.34a) and (2.34b), e.g. for all Borel functions $\varphi _i:\mathbb {R}^d \rightarrow [-\infty ,+\infty ]$ with

2.36 $$\begin{aligned}{} & {} \int _{\mathbb {R}^d}\textrm{e}^{-2\tau \varphi _\tau }\;\!\textrm{d}\mu _\tau<\infty ,\quad \int _{\mathbb {R}^d}\textrm{e}^{2\tau \varphi _0}\;\!\textrm{d}\mu _0<\infty ,\nonumber \\{} & {} \varphi _\tau (x_1) \leqq \textrm{L}_\tau (x_1{-}x_0) +\varphi _0(x_0) \quad \text {for all } x_0,x_\tau \in \mathbb {R}^d\text { with }|x_0{-}x_\tau |<\pi /2,\nonumber \\ \end{aligned}$$

we have

2.37 $$\begin{aligned} \frac{1}{2\tau }\textsf{H}\!\!\textsf{K}(\mu _0,\mu _\tau )^2 \geqq \int _{\mathbb {R}^{d}}\textsf{G}_\tau (\varphi _\tau )\;\!\textrm{d}\mu _\tau -\int _{\mathbb {R}^{d}}\check{\textsf{G}}_\tau (\varphi _0)\;\!\textrm{d}\mu _0. \end{aligned}$$

If we allow extended valued Borel functions, the supremum in (2.34a) and (2.34b) are attained.

##### Theorem 2.12

(Existence of optimal dual pairs) For all $\mu _0,\mu _\tau \in \mathcal {M}(\mathbb {R}^d)$ and $\tau >0$ there exists an optimal pair of Borel potentials $\varphi _0,\varphi _\tau :\mathbb {R}^d\rightarrow [-\infty ,+\infty ]$ which is admissible according to (2.36) and realizes equality in (2.37), namely

2.38 $$\begin{aligned} \frac{1}{2\tau }\textsf{H}\!\!\textsf{K}(\mu _0,\mu _\tau )^2 =\int _{\mathbb {R}^{d}}\textsf{G}_\tau (\varphi _\tau )\;\!\textrm{d}\mu _\tau - \int _{\mathbb {R}^{d}}\check{\textsf{G}}_\tau (\varphi _0)\;\!\textrm{d}\mu _0. \end{aligned}$$

The transformations $\xi _0:=\check{\textsf{G}}_\tau (\varphi _0):\mathbb {R}^d\rightarrow [ -1/(2\tau ), +\infty ]$, and $\xi _\tau :=\textsf{G}_\tau (\varphi _\tau ):\mathbb {R}^d\rightarrow [-\infty ,1/(2\tau ) ]$, give an optimal pair for (2.34b) (dropping $\xi _i\in \textrm{C}_b(\mathbb {R}^d)$) satisfying

2.39 $$\begin{aligned} \int _{\mathbb {R}^d}|\xi _i|\;\!\textrm{d}\mu _i&<\infty ,\quad i=0,\tau , \end{aligned}$$

2.40 $$\begin{aligned} (1{-}2\tau \xi _\tau (x_\tau ))( {1{+}2\tau \xi _0(x_0)})&\geqq \cos ^2_{{\pi /2}}\!{\left( |x_0{-}x_\tau |\right) } \quad \text {if }\xi _0(x_0)<\infty , \ \xi _\tau (x_\tau ) > - \infty , \end{aligned}$$

2.41 $$\begin{aligned} \frac{1}{2\tau } \textsf{H}\!\!\textsf{K}(\mu _0,\mu _\tau )^2&=\int _{{\mathbb {R}^{d}}}\xi _\tau \;\!\textrm{d}\mu _\tau - \int _{{\mathbb {R}^{d}}} \xi _0\,\;\!\textrm{d}\mu _0. \end{aligned}$$

##### Remark 2.13

Denoting by $S_i: ={\text {supp}}(\mu _i)$ the support of $\mu _i$ for $i=0$ and 1, we remark that it is always sufficient to find Borel potentials $\varphi _i:S_i\rightarrow [-\infty ,+\infty ]$ satisfying (2.36) on $S_0\times S_1$ instead of $\mathbb {R}^d \times \mathbb {R}^d$. By setting ${\tilde{\varphi }}_1:=-\infty$ in $\mathbb {R}^d\setminus S_1$ and ${\tilde{\varphi }}_0:=+\infty$ in $\mathbb {R}^d\setminus S_0$ we obtain a pair still satisfying (2.36) and (2.38). This freedom will be useful in Theorem 2.14 below.

Moreover, notice that (2.34b) can be rewritten as

$$\begin{aligned} \frac{1}{2\tau }\textsf{H}\!\!\textsf{K}(\mu _0,\mu _\tau )^2 = \sup \bigg \{ \int _{\mathbb {R}^{d}}{\mathscr {P}}_{\hspace{-2.0pt}\tau }\xi _0 \;\!\textrm{d}\mu _\tau - \int _{\mathbb {R}^{d}}\xi _0\;\!\textrm{d}\mu _0\,\Big |\, \xi _0\in \textrm{C}_b({\mathbb {R}^{d}}),\ \xi _0 > - \frac{1}{2\tau } \bigg \}, \end{aligned}$$

where ${\mathscr {P}}_{\hspace{-2.0pt}\tau }\hspace{1.0pt}\xi$ is defined in (1.13). In particular, the operator ${\mathscr {P}}_\tau$ is directly connected to the dynamical formulation in (2.3), and we will thoroughly study its properties in Section 4.

### First Order Optimality for $\textsf{H}\!\!\textsf{K}$

From the above discussion, we have already seen that there is never any transport over distances larger than $\pi /2$. This transport bound will also be seen in the following optimality conditions for the marginal densities $\sigma _i$ defined in (2.24).

#### Theorem 2.14

(Optimality conditions [27, Thm. 6.3]) Let $\mu _0,\mu _\tau \in \mathcal {M}({\mathbb {R}^{d}})$ and let $S_i,S_i',S_i'',\mu _i'$ be defined as in (2.12)–(2.13). The following holds:

1. A plan ${\varvec{\eta }}\in \mathcal {M}({\mathbb {R}^{d}}\times {\mathbb {R}^{d}})$ is optimal for the logarithmic entropy-transport problem in (2.30) if and only if
  $$\iint \ell \;\!\textrm{d}{\varvec{\eta }}<\infty $$
  its marginals $\eta _i$ are absolutely continuous with respect to $\mu _i'$ (equivalently, $\eta _i$ are absolutely continuous with respect to $\mu _i$ and $\eta _i(S_i'')=0$),
  there exist Borel densities $\sigma _i: \mathbb {R}^d\rightarrow [0,\infty ]$ such that $\eta _i=\sigma _i \mu _i'$ and
    2.42a $$\begin{aligned} \sigma _i=0\quad&\text {on }S_i'', \end{aligned}$$
    2.42b $$\begin{aligned} 0<\sigma _i<\infty \quad&\text {on }S_i', \end{aligned}$$
    2.42c $$\begin{aligned} \sigma _i=+\infty \quad&\text {on }\mathbb {R}^d\setminus S_i, \end{aligned}$$
    2.42d $$\begin{aligned} \sigma _0(x_0)\sigma _\tau (x_\tau )\geqq \cos ^2_{{\pi /2}}\!{\left( |x_0 {-}x_\tau |\right) }\quad&\text {on } S_0\times S_\tau , \end{aligned}$$
    2.42e $$\begin{aligned} \sigma _0(x_0)\sigma _\tau (x_\tau )= \cos ^2_{{\pi /2}}\!{\left( |x_0 {-}x_\tau |\right) } \quad&{\varvec{\eta }}\text {-a.e.\,on } S_0\times S_\tau . \end{aligned}$$
  In particular, the marginals $\eta _i$ are unique and the densities $\sigma _i$ are unique $\mu '_i$-a.e.
2. If ${\varvec{\eta }}$ is optimal and $S_i,\,S_i',\,S_i''$ and $\sigma _i$ are defined as above, the pairs of potentials defined by
  2.43 $$\begin{aligned} \varphi _\tau :={}&{\left\{ \begin{array}{ll} -\frac{1}{2\tau }\log \sigma _\tau &{}\text {in }S_\tau ',\\ +\infty &{}\text {in }S_\tau '',\\ -\infty &{}\text {in }{\mathbb {R}^{d}}\setminus S_\tau ; \end{array}\right. }&\varphi _0:={}&{\left\{ \begin{array}{ll} \frac{1}{2\tau } \log \sigma _0 &{}\text {in }S_0',\\ -\infty &{}\text {in }S_0'',\\ +\infty &{}\text {in }{\mathbb {R}^{d}}\setminus S_0; \end{array}\right. } \end{aligned}$$
  2.44 $$\begin{aligned} \xi _\tau :={}&{\left\{ \begin{array}{ll} \frac{1{-}\sigma _\tau }{2\tau } &{}\text {in }S_\tau ',\\ \frac{1}{2\tau }&{}\text {in }S_\tau '',\\ -\infty &{}\text {in }{\mathbb {R}^{d}}\setminus S_\tau ; \end{array}\right. }&\xi _0:={}&{\left\{ \begin{array}{ll} \frac{\sigma _0{-}1}{2\tau } &{}\text {in }S_0',\\ -\frac{1}{2\tau }&{}\text {in }S_0'',\\ +\infty &{}\text {in }{\mathbb {R}^{d}}\setminus S_0; \end{array}\right. } \end{aligned}$$
  are optimal in the respective dual relaxed characterizations of Theorem 2.12 and satisfy ${\varvec{\eta }}$-a.e. in $\mathbb {R}^d\times \mathbb {R}^d$
  2.45a $$\begin{aligned} \varphi _i(x_i)&\in \mathbb {R},&\varphi _\tau (x_\tau )-\varphi _0(x_0)&= \textrm{L}_\tau (x_\tau {-}x_0), \end{aligned}$$
  2.45b $$\begin{aligned} -\xi _0(x_0),\xi _\tau (x_\tau )&\in \big (\frac{1}{2\tau },\infty \big ),\hspace{-6.0pt}&(1{+}2\tau \xi _0(x_0))(1{-}2\tau \xi _\tau (x_\tau ))&= \cos ^2_{{\pi /2}}\!{\left( |x_0{-}x_\tau |\right) }. \end{aligned}$$
3. Conversely, if ${\varvec{\eta }}$ is optimal and $(\varphi _0,\varphi _\tau )$ (resp. $(\xi _0,\xi _\tau )$) is an optimal pair according to Theorem 2.12, then (2.45a) (resp. (2.45b)) holds ${\varvec{\eta }}$-a.e. and
  2.46 $$\begin{aligned} \begin{aligned} \sigma _\tau ={}&\textrm{e}^{-2\tau \varphi _\tau }=1{-}2\tau \xi _\tau \hspace{-4.0pt}&\mu _\tau \,\text {-a.e. in }\,S_\tau ',{} & {} \varphi _\tau ={}&+\infty , \ \xi _\tau =\frac{1}{2\tau }&\mu _\tau \,\text {-a.e. in}\,S_\tau '', \\ \sigma _0={}&\textrm{e}^{2\tau \varphi _0}=1{+}2\tau \xi _0 \hspace{-4.0pt}&\mu _0\,\text {-a.e. in}\,S_0',{} & {} \varphi _0={}&-\infty , \ \xi _0=-\frac{1}{2\tau }&\mu _0\,\text {-a.e. in}\,S_0''. \end{aligned}\nonumber \\ \end{aligned}$$

## Regularity of Static $\textsf{H}\!\!\textsf{K}$ Potentials $\varphi _0$ and $\varphi _1$

In this section, we will carefully study the regularity of a pair $(\varphi _0,\varphi _1)$ of optimal $\textsf{H}\!\!\textsf{K}$ potentials arising in (2.43) of Theorem 2.14. We will improve the previous approximate differentiability result of [27, Thm. 6.6(iii)] (see also [2, Thm. 6.2.7]) by adapting the argument of [14] and extending the classical result of [16] to the $\textsf{H}\!\!\textsf{K}$ setting. In fact, this section is largely independent of the specific $\textsf{H}\!\!\textsf{K}$ setting but relies purely on the theory of $\mathbb {L}$-transforms. As we are interested in the special case of continuous, extended values cost functions $\mathbb {L}= \textrm{L}_\tau =\frac{1}{\tau } \textrm{L}_1:\mathbb {R}^d\rightarrow [0,+\infty ]$ which attain the value $+\infty$ outside a ball, we cannot rely on existing results and have to provide a careful analysis of this case (but see also [7, 8, 18, 20, 29] for different situations of discontinuous costs taking the value $+\infty$).

We will use the notion of locally semi-concave and semi-convex functions; recall that a function $\varphi :U\rightarrow \mathbb {R}$ defined in some open set *U* of $\mathbb {R}^d$ is locally semi-concave if for every point ${\bar{x}}\in U$ there exists $\rho >0$ and a constant $C>0$ with

3.1 $$\begin{aligned} x\mapsto \varphi (x)-\frac{C}{2} |x|^2 \quad \text {is concave in }B_\rho ({\bar{x}}). \end{aligned}$$

A function $\varphi$ is locally semi-convex if $-\varphi$ is locally semi-concave. Let us recall that locally semi-concave functions are locally Lipschitz and thus differentiable almost everywhere. We will denote by $\textrm{dom}(\nabla \varphi )$ the domain of their differential. By Alexandrov’s Theorem (see [2, Thm. 5.5.4]), there exists for almost every $x\in \textrm{dom}(\nabla \varphi )$ a symmetric matrix $\mathsf A=:\textrm{D}^2 \varphi ( x)$ such that

3.2a $$\begin{aligned}&\lim _{y\rightarrow x}\frac{\varphi (y)-\varphi (x)-\langle \nabla \varphi (x),y{-}x\rangle -\frac{1}{2} \langle \textsf{A}(y{-}x),y{-}x\rangle }{|y{-}x|^2}=0, \end{aligned}$$

3.2b $$\begin{aligned}&\text {and} \quad \lim _{\underset{y\in \textrm{dom}(\nabla \varphi )}{y\rightarrow x}}\frac{\nabla \varphi (y)-\nabla \varphi (x)- \textsf{A}(y{-}x)}{|y{-}x|}=0. \end{aligned}$$

We will denote by $\textrm{dom}(\textrm{D}^2\varphi )$ the subset of density points in $\textrm{dom}(\nabla \varphi )$ where (3.2a) and (3.2b) hold.

As the optimality of potential pairs $(\varphi _0,\varphi _1)$ is closely related to the theory of $\mathbb {L}$-transforms, we give the basic definitions first and then derive the associated regularity properties under additional smoothness assumptions.

For simplicity, we restrict the analysis of the remaining text to continuous functions $\mathbb {L}: \mathbb {R}^d \rightarrow [0,\infty ]$ satisfying $\textrm{dom}(\mathbb {L}) = \big \{\, z \in \mathbb {R}^d \, \big | \, \mathbb {L}(z)\in \mathbb {R} \,\big \} =B_{\mathsf R}(0)$ for some ${\mathsf R}>0$, i.e. $\mathbb {L}(z)<\infty$ for $|z|<\mathsf R$ and $\mathbb {L}(z)=+\infty$ for $|z|\geqq \mathsf R$. By continuity of $\mathbb {L}$ this behavior implies $\mathbb {L}(z_k)\rightarrow +\infty$ if $\liminf _{k\rightarrow \infty } |z_k| \geqq \mathsf R$.

We define the *forward* $\mathbb {L}$*-transform* $\varphi _0^{\mathbb {L}\rightarrow }$ of a l.s.c. function $\varphi _0$ and the *backward* $\mathbb {L}$*-transform*  of an u.s.c. function $\varphi _1$ via3.3where the restriction of the infimum and supremum in (3.3) to the balls $B_\mathsf R(x_i)$, corresponding to the shifted proper domain of $\mathbb {L}$, is important to avoid the expression “$\infty - \infty$”. It will turn out that $\varphi _0^{\mathbb {L}\rightarrow }$ is u.s.c. and  is l.s.c. Of course, these transformations are related by3.4and for arbitrary functions $\psi _i:\mathbb {R}^d \rightarrow [-\infty ,+\infty ]$ we have the general relations3.5see [34, Ch. 5]. For later usage, we consider the following elementary example.

### Example 3.1

(Forward and backward $\mathbb {L}$-transform) We consider the potentials

$$\begin{aligned} \varphi _0(x_0) = {\left\{ \begin{array}{ll} a_0 &{}\text {for } x_0=y_0, \\ +\infty &{} \text {otherwise}, \end{array}\right. } \quad \text { and }\quad \varphi _1(x_1)= {\left\{ \begin{array}{ll} a_1 &{}\text {for } x_1=y_1, \\ -\infty &{} \text {otherwise}, \end{array}\right. } \end{aligned}$$

where $-\infty \leqq a_0 <+\infty$, $-\infty < a_1 \leqq +\infty$ and $y_0,y_1\in \mathbb {R}^d$ are fixed. For $a_0,a_1 \in \mathbb {R}$ we find the transformsFor $a_0=-\infty$ and $a_1 = + \infty$, we obtain the transformsAs $B_\mathsf R(y_i)$ is open, we see that $\varphi ^{\mathbb {L}\rightarrow }_0$ is u.s.c. and  is l.s.c. Moreover, observe that  and , so that (3.5) is true for  and $\psi _1 \in \big \{\varphi _1, \varphi _0^{\mathbb {L}\rightarrow } \big \}$, respectively.

For $\mathsf R>0$ and sets $S\subset \mathbb {R}^d$, we introduce the notation

3.6 $$\begin{aligned}{} & {} S^{\mathsf R}:= \big \{\, x\in \mathbb {R}^d \, \big | \, \textrm{dist}(x,S)< \mathsf R \,\big \} ,\nonumber \\ {}{} & {} {\text {ext}}_{\mathsf R}(S):=\bigcup _{x:\ \textrm{dist}(x,S)> \mathsf R} B_{\mathsf R}(x),\quad {\text {bdry}}_{\mathsf R}(S):=\partial S\cap \partial \big ({\text {ext}}_{\mathsf R}(S)\big ). \end{aligned}$$

In particular, ${\text {ext}}_{\mathsf R}(S)$ is the open subset of $\mathbb {R}^d\setminus S$ obtained by taking the union of all the open balls of radius ${\mathsf R}$ that do not intersect *S*. If *S* is closed and satisfies an exterior sphere condition of radius ${\mathsf R}$ at every point of its boundary (e.g. if *S* is convex) then ${\text {ext}}_{\mathsf R}(S)$ coincides with $\mathbb {R}^d{\setminus } S$ and ${\text {bdry}}_{\mathsf R}(S)=\partial S$.

In general, ${\text {bdry}}_{\mathsf R}(S)$ is a subset of the boundary of *S*, precisely made by all points of $\partial S$ satisfying an exterior sphere condition of radius ${\mathsf R}$ with respect to *S*:

3.7 $$\begin{aligned} x\in {\text {bdry}}_{\mathsf R}(S)\quad \Longleftrightarrow \quad x\in \partial S\text { and }\exists \,y\in \mathbb {R}^d: \ |x{-}y|={\mathsf R},\ B_{\mathsf R}(y)\cap S=\emptyset . \nonumber \\ \end{aligned}$$

**Fig. 2:**
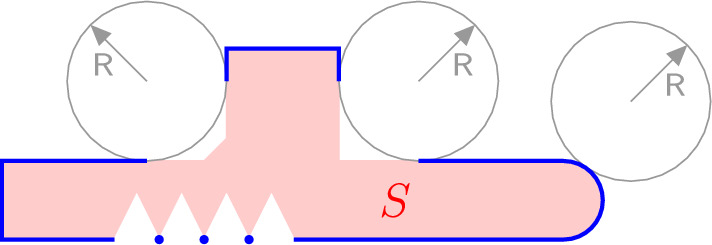
Visualization of ${\text {bdry}}_{\mathsf R}(S)$ (thick) as subset of the boundary $\partial S$ of the set *S* (light red)

In fact, if $x\in {\text {bdry}}_{\mathsf R}(S)$ then there exist sequences $x_n, y_n$ such that $x_n\rightarrow x$, $|x_n{-}y_n|<\mathsf R$ and $B_{\mathsf R}(y_n)\cap S=\emptyset$. Possibly extracting a subsequence, we can assume that $y_n\rightarrow y$, $B_{\mathsf R} (y)\cap S=\emptyset$, and $|x{-}y|\leqq \mathsf R$. Since $x\in \partial S$, it is not possible that $|x{-}y|<{\mathsf R}$, so that the left-to-right implication of (3.7) holds. On the other hand, if $x\in \partial S$, $|x{-}y|=\mathsf R$, and $B_\mathsf R(y)\cap S=\emptyset$, it is immediate to check that $x\in \partial ({\text {ext}}_{\mathsf R}(S))$, see also Fig. 2.

In Theorem 3.3(2) we will use that for arbitrary sets *S* the boundary part ${\text {bdry}}_{\mathsf R}(S)$ is countably $(d{-}1)$-rectifiable, see [34, Th. 10.48(ii)], and hence has $\mathcal {L}^{d}$ measure 0.

The next result shows how the properties of $\mathbb {L}$ provide regularity of the backward transform . Of course, an analogous statement holds for the forward transform using (3.4). The important fact is that the *upper bounds* on the second derivatives of $\mathbb {L}$ generate semi-convexity of $\varphi _0$ (i.e. *lower bounds* on $\textrm{D}^2\varphi _0$), see Assertions 5 and 6. As $\textrm{D}^2 \mathbb {L}(z)$ blows up at the boundary of $B_\mathsf R(0)$, it is essential to use the fact that $\mathbb {L}(z_k)\rightarrow +\infty$ for $|z_k|\uparrow \mathsf R$.

### Theorem 3.2

(Regularity of the $\mathbb {L}$-transform) Let $\mathbb {L}: \mathbb {R}^d\rightarrow [0,+\infty ]$ satisfy

3.8a $$\begin{aligned}&\mathbb {L}: \mathbb {R}^d\rightarrow [0,+\infty ] \text { is continuous and } \mathbb {L}(0)=0, \end{aligned}$$

3.8b $$\begin{aligned}&\mathbb {L}\big |_{B_\mathsf R(0)} \in \textrm{C}^2(B_\mathsf R(0)) \text { and } \mathbb {L}(z)=+\infty \text { if } |z|\geqq \mathsf R, \end{aligned}$$

3.8c $$\begin{aligned}&\mathbb {L}\text { is uniformly convex, i.e.\ } \exists \, \lambda _*>0 \ \forall \, z \in B_\mathsf R(0): \ \textrm{D}^2\mathbb {L}(z) \geqq \lambda _* I. \end{aligned}$$

For an u.s.c. function $\varphi _1:\mathbb {R}^d\rightarrow [-\infty ,+\infty ]$, we consider the backward $\mathbb {L}$-transform  and set

3.9 $$\begin{aligned} \begin{aligned}&O_0 = \{\varphi _0>-\infty \}, \quad Q_0=\{\varphi _0<+\infty \}, \\&O_1 = \{\varphi _1<+\infty \}, \quad Q_1=\{\varphi _1>-\infty \},\quad \text {and} \quad \Omega _0 = O_0\cap \textrm{int}(Q_0). \end{aligned} \end{aligned}$$

Then, the following assertions hold:

1. The function $\varphi _0$ is l.s.c. and satisfies
  3.10 $$\begin{aligned}&\inf \varphi _0\geqq \inf \varphi _1 \quad \text {and} \quad \sup \varphi _0\leqq \sup \varphi _1, \end{aligned}$$
  3.11 $$\begin{aligned}&\quad (Q_1)^{\mathsf R} \subset O_0 \quad \text {and} \quad \Big (\varphi _0(x_0)=-\infty \ \Leftrightarrow \ B_\mathsf R(x_0)\subset {\text {ext}}_{\mathsf R}(Q_1)\subset \{\varphi _1=-\infty \} \Big ). \end{aligned}$$
  The sets $O_0,\ O_1$, and $\Omega _0$ are open.
2. The set $Q_0$ satisfies an external sphere condition of radius $\mathsf R$, namely
  3.12 $$\begin{aligned} \mathbb {R}^d\setminus {\text {cl}}(Q_0)={\text {ext}}_{\mathsf R}(Q_0)\quad \text {and} \quad \partial Q_0={\text {bdry}}_{\mathsf R}(Q_0), \end{aligned}$$
  so that the topological boundary of $Q_0$ is countably $(d{-}1)$-rectifiable.
3. The “contact set” $M:= M_{-\infty }\cup M_{+\infty } \cup M_\textrm{fin}\subset \mathbb {R}^d\times \mathbb {R}^d$ defined via
  3.13 $$\begin{aligned} \begin{aligned} M_\textrm{fin}:={}&\big \{\, (x_0,x_1) \, \big | \, \varphi _i(x_i)\in \mathbb {R}, ~\varphi _1(x_1)=\mathbb {L}(x_0{-}x_1) +\varphi _0(x_0) \,\big \} , \\ M_{-\infty }:={}&\big \{\, (x_0,x_1) \, \big | \, \varphi _0(x_0)=-\infty ,\ |x_1{-}x_0|\geqq \mathsf R \,\big \} , \\ M_{+\infty }:={}&\big \{\, (x_0,x_1) \, \big | \, \varphi _1(x_1)=+\infty ,\ |x_1{-}x_0|\geqq \mathsf R \,\big \} , \end{aligned} \end{aligned}$$
  is closed.
4. For every ${\bar{x}}_0\in \Omega _0$, the section $M_{0\rightarrow 1}[{\bar{x}}_0]:= \big \{\, x_1 \, \big | \, ({\bar{x}}_0,x_1)\in M_{\textrm{fin}} \,\big \}$ of $M_{\textrm{fin}}$ is nonempty, compact, and included in $Q_1$. Moreover, for every compact $K\subset \Omega _0$ there exists $\theta \in (0,\mathsf R)$ and $a',a''\in \mathbb {R}$ such that
  3.14 $$\begin{aligned} |x_1{-}{\bar{x}}_0|\leqq \theta \text { and } a'\leqq \varphi _1(x_1)\leqq a'' \quad \text {whenever } {\bar{x}}_0\in K \text { and } x_1\in M_{0\rightarrow 1}[{\bar{x}}_0].\nonumber \\ \end{aligned}$$
5. The restriction of $\varphi _0$ to the open set $\Omega _0$ is locally semi-convex, and in particular locally Lipschitz and thus continuous.
6. If $D_0':=\textrm{dom}(\nabla \varphi _0)\subset \Omega _0$, $D_0''=\textrm{dom}(\textrm{D}^2\varphi _0) \subset D_0'$, then $D''_0$ has full Lebesgue measure in $\Omega _0$. For every $x\in D_0'$, the set $M_{0\rightarrow 1}[x]$ contains a unique point $y=\varvec{T}_{0\rightarrow 1}(x)$. The induced map $\varvec{T}_{0\rightarrow 1}:D_0'\rightarrow \mathbb {R}^d$ is differentiable according to (3.2b) in $D_0''$ and satisfies the following properties:
  3.15 $$\begin{aligned} \text {(a) }&|x{-}\varvec{T}_{0\rightarrow 1}(x)|<\mathsf R\text { and } \nabla \varphi _0(x) = (\nabla \mathbb {L})\big (x{-}\varvec{T}_{0\rightarrow 1}(x)\big ) \text { for all }x\in D_0', \end{aligned}$$
  3.16 $$\begin{aligned} \text {(b) }&\textrm{D}^2 \varphi _0(x) \geqq -\textrm{D}^2\mathbb {L}\,\big (x{-}\varvec{T}_0(x)\big ) \quad \text {for all } x\in D_0'', \end{aligned}$$
  3.17 $$\begin{aligned} \text {(c) }&\textrm{D}\varvec{T}_{0\rightarrow 1}(x) \text { is diagonalizable with nonnegative eigenvalues on } D_0''. \end{aligned}$$

### Proof

We divide the proof in various steps, corresponding to each assertion.

Assertion (1). To check that $\varphi _0$ is l.s.c. we assume $\varphi _0(x_0)>a$ for some $a\in [-\infty ,+\infty )$, then there exists $y \in B_\mathsf R(x_0)$ such that $\varphi _1(y)-\mathbb {L}(y{-}x_0)>a$. As $\mathbb {L}$ is continuous, we can find $\delta \in (0,\mathsf R-|y{-}x_0|)$ such that $\varphi _1(y)-\mathbb {L}(y{-}x)> a$ for every $x\in B_\delta (x_0)$. By definition of $\varphi _0$ this estimate implies $\varphi _0(x)>a$ on $B_\delta (x_0)$, and lower semi-continuity is shown.

The estimates in (3.10) are elementary following from $\mathbb {L}(0)=0$ and $\mathbb {L}(z)\geqq 0$, respectively. The relation in (3.11) follows from the fact that $\varphi _0(x_0)=-\infty$ implies $\varphi _1(y)\equiv -\infty$ in $B_\mathsf R(x_0)$. The openness of $O_0$ and $O_1$ follows because $\varphi _0$ is l.s.c. and $\varphi _1$ is u.s.c. This property in turn implies that $\Omega _0= O_0 \cap \textrm{int}(Q_0)$ is open.

Assertion 2. Recalling $Q_0=\{\varphi _0<+\infty \}$ it is sufficient to notice that

3.18a $$\begin{aligned}&{\bar{x}} \in \mathbb {R}^d\setminus Q_0 \ \Leftrightarrow \ \varphi _0({\bar{x}})=+\infty \ \Rightarrow \ \exists \,{\bar{y}}: |{\bar{x}}{-}{\bar{y}}|\leqq \mathsf R\text { and } \varphi _1(\bar{y})=+\infty , \end{aligned}$$

where we used $\mathbb {L}\geqq 0$ and the upper semicontinuity of $\varphi _1$. However, using $\textrm{dom}(\mathbb {L})=B_\mathsf R(0)$ we obtain

3.18b $$\begin{aligned} \varphi _1({\bar{y}})=+\infty \quad \Rightarrow \quad \varphi _0(x)=+\infty \text { for all } x\in B_{\mathsf R}({\bar{y}}). \end{aligned}$$

This implication means that if ${\bar{x}}\in \mathbb {R}^d\setminus Q_0$ then ${\bar{x}}\in {\text {cl}}({\text {ext}}_{\mathsf R}(Q_0))$, so that $\partial Q_0=\partial (\mathbb {R}^d{\setminus } Q_0)= \partial {\text {cl}}( {\text {ext}}_{\mathsf R}(Q_0))=\partial {\text {ext}}_{\mathsf R}(Q_0)$.

Assertion 3. The closedness of $M_{\pm \infty }$ follows easily by the semi-continuities of $\varphi _i$. For $M_\text {fin}$ we consider a sequence $(x_{0,n},x_{1,n})\in M_{\textrm{fin}}$ to $(x_0,x_1)$. If $|x_0{-}x_1|< \mathsf R$, then we have $\varphi _1(x_1)\geqq \mathbb {L}(x_1{-}x_0) + \varphi _0(x_0)$ by the semi-continuities. As the opposite inequality is always satisfied, we obtain the equality. We can also exclude that $\varphi _0(x_0)=\varphi _1(x_1)=+\infty$ (resp. $-\infty$), since otherwise $\varphi _0(x)\equiv +\infty$ in $B_\mathsf R(x_1)$ by (3.18b) which contains a neighborhood of $x_0$ (resp. $\varphi _1(x)\equiv -\infty$ in $B_\mathsf R(x_0)$ by (3.11), which contains a neighborhood of $x_1$), so that $(x_0,x_1)\in M_{\textrm{fin}}$. If $|x_1{-}x_0|\geqq \mathsf R$ and $(x_0,x_1)$ does not belong to $M_{-\infty }$ then we have $\liminf _{n\rightarrow \infty } \varphi _0(x_{0,n})\geqq \varphi _0(x_0)>-\infty$ so that

$$\begin{aligned} \varphi _1(x_1)\geqq \limsup _{n\rightarrow \infty }\varphi _1(x_{1,n})= \limsup _{n\rightarrow \infty }\mathbb {L}(x_{1,n}-x_{0,n})+\varphi _1(x_{0,n})= +\infty \end{aligned}$$

and $(x_0,x_1)\in M_{+\infty }$. Hence, $M=M_\textrm{fin}\cup M_{+\infty } \cup M_{-\infty }$ is closed.

Assertion 4. Let us first show that $\varphi _0$ *is locally bounded from above in the interior of* $Q_0$, *i.e. the open set* $Q_0\setminus \partial Q_0$. In fact, if a sequence $x_n$ is converging to ${\bar{x}}\in Q_0{\setminus } \partial Q_0$ with $\varphi _0(x_n) \uparrow +\infty$, by arguing as before and using $\varphi _0(x_n)=\sup _{y\in B_{\mathsf R}(x_n)}\varphi _1(y)-\mathbb {L}(y {-}x_n)$, we find ${\bar{y}}\in \overline{B_{\mathsf R}({\bar{x}})}$ with $\varphi _1(\bar{y})=+\infty$. Now (3.18b) gives $\varphi _0(x)=+\infty$ for all $x\in B_\mathsf R({\bar{y}})$, which contradicts the fact that $\varphi _0(x) < +\infty$ in a neighborhood of $\bar{x}$, because of $|{\bar{x}}{-}{\bar{y}}|\leqq \mathsf R$.

We fix now a compact subset *K* of the open set $\Omega _0$, a point ${\bar{x}}\in K$, and consider the section $M_{0\rightarrow 1}[{\bar{x}}]$ of the contact set $M_{\textrm{fin}}$. Let $\eta >0$ be sufficiently small so that $K_\eta := \big \{\, x\in \mathbb {R}^d \, \big | \, \textrm{dist}(x,K)\leqq \eta \,\big \} \subset \Omega _0$ and let $\overline{a}:=\sup _{K_\eta } \varphi _0$, where $a<+\infty$ by the previous claim. By l.s.c. of $\varphi _0$, we also have ${\underline{a}}:= \inf _{K_\eta } \varphi _0 >-\infty$.

By the definition of , for every $\varepsilon \in (0,1]$ the sets

3.19 $$\begin{aligned} M^\varepsilon ({\bar{x}}):= \Big \{\, y\in B_{\mathsf R}({\bar{x}}) \; \Big | \; \varphi _1(y) \geqq \mathbb {L}( y {-} {\bar{x}}) +\varphi _0({\bar{x}}) -\varepsilon \,\Big \} , \end{aligned}$$

are non-empty. We choose $y\in M^1({\bar{x}})$ and set $x_\vartheta :=\vartheta {\bar{x}}+ (1 {-}\vartheta ) y$ with $\vartheta = 1-\eta /\mathsf R$, which implies $|x_\vartheta {-}{\bar{x}}| \leqq \eta$, and hence $x_\vartheta \in K_\eta$. Moreover, we have $|x_\vartheta {-}y|\leqq \mathsf R-\eta$. Therefore, for $y \in M^1({\bar{x}}) \subset B_\mathsf R({\bar{x}})$ we find

3.20 $$\begin{aligned} \begin{aligned} \varphi _1(y)&\leqq \mathbb {L}(y{-} x_\vartheta )+\varphi _0(x_\vartheta ) \leqq a'':= \overline{a} + \widehat{\ell }(\mathsf R{-}\eta ) < \infty , \\ \varphi _1(y)&\geqq \varphi _0({\bar{x}}) + \mathbb {L}(y{-}{\bar{x}}) -1\geqq a':= {\underline{a}} >-\infty , \end{aligned} \end{aligned}$$

where $\widehat{\ell }(\varrho ):= \sup _{z\in B_{\varrho }(0)} \mathbb {L}(z)$. Combining the last two estimates we additionally find

3.21 $$\begin{aligned} \mathbb {L}(y{-}{\bar{x}})\leqq \varphi _1(y)-\varphi _0({\bar{x}}) \leqq a''-{\underline{a}} =: \widehat{\ell }(\theta ) \quad \text {with }\quad \theta \in (0, \mathsf R). \end{aligned}$$

Hence, all elements $y \in M^1({\bar{x}})$ satisfies $|{\bar{x}}{-}y|\leqq \theta$ and (3.20).

We now consider a sequence $y_\varepsilon \in M^\varepsilon ({\bar{x}})\subset M^1({\bar{x}})$, then a standard compactness argument and the upper semi-continuity of $\varphi _1$ show that any limit point ${\bar{y}}$ is an element of $M_{0\rightarrow 1}[{\bar{x}}]$, which is therefore not empty. The compactness of $M_{0\rightarrow 1}[{\bar{x}}]$ and (3.14) again follow by (3.21)

Assertion 5. Let us now fix ${\bar{x}}_0\in \Omega _0$ and $\delta >0$ such that $K:=\overline{B_\delta ({\bar{x}}_0)}\subset \Omega _0$. The previous assertion yields $\theta <\mathsf R$ and $a',a''\in \mathbb {R}$ such that $|x'{-}x|\leqq \theta$ and $a'\leqq \varphi _1(x')\leqq a''$ whenever $x\in K$ and $x'\in M_{0\rightarrow 1}[x]$. By possibly reducing $\delta$, we can also assume that $3\delta +\theta <\mathsf R$. For every $x\in K$, we now have by construction

3.22 $$\begin{aligned} \varphi _0(x)=\max _{x'\in \overline{B_{\delta +\theta }({\bar{x}}_0)}}\varphi _1(x')-\mathbb {L}(x'{-}x) \end{aligned}$$

which is bounded and semi-convex in *K* because it is a supremum over a family of uniformly semi-convex functions, where we use $| x'{-}x|\leqq |x'{-}{\bar{x}}_0| + |{\bar{x}}_0{-}x|\leqq 2\delta {+} \theta$ and that $-\mathbb {L}$ is semi-convex on $\overline{B_{2\delta {+} \theta }({\bar{x}}_0)}$ by (3.8b).

Assertion 6. This assertion follows in the standard way by using the extremality conditions in the contact set, see e.g.  [2, Thm. 6.2.4 and 6.2.7]. We give the main argument to show how the assumptions in (3.8) enter. By Alexandrov’s theorem and Assertion 5 the set $D''_0$ has full Lebesgue measure. To obtain the optimality conditions, we fix $x_0\in Q_0\cap D''_0$ and know from (3.22) that there exists ${\bar{x}}_1$ such that $\varphi _0(x_0)=\varphi _1({\bar{x}}_1)- \mathbb {L}({\bar{x}}_1{-}x_0)$. However, for all $x\in B_\delta (x_0)$ we have $\varphi _0(x)+\mathbb {L}({\bar{x}}_1{-}x)\geqq \varphi _1({\bar{x}}_1)$ with equality for $x=x_0$. Thus, we obtain the optimality conditions

$$\begin{aligned} \nabla \varphi _0(x_0)- \nabla \mathbb {L}({\bar{x}}_1{-}x_0)=0 \text { in } \mathbb {R}^d \quad \text {and} \quad \textrm{D}^2 \varphi _0(x_0) + \textrm{D}^2\mathbb {L}({\bar{x}}_1{-}x_0)\geqq 0 \text { in } \mathbb {R}^{d\times d}_\text {sym}. \end{aligned}$$

This result gives the conditions (a) to (c), if we observe that ${\bar{x}}_1$ is unique. But this property follows from the first optimality condition by using (3.8c) which allows us to write

$$\begin{aligned} {\bar{x}}_1 = \varvec{T}_{0\rightarrow 1}(x_0):= x_0+ \big ( \nabla \mathbb {L}\big )^{-1}(\nabla \varphi _0(x_0)), \end{aligned}$$

i.e. ${\bar{x}}_1$ is uniquely determined by $x_0$. Moreover, $\textrm{D}\varvec{T}_{0\rightarrow 1}(x_0)$ exists and satisfies $\textrm{D}^2 \varphi _0(x_0)= (\textrm{D}^2 \mathbb {L})(\varvec{T}_{0\rightarrow 1}(x_0){-}x_0) \big (\textrm{D}\varvec{T}_{0\rightarrow 1}(x_0){-}I\big )$, which implies the diagonalization result. $\quad \square$

The previous result can now be applied to the solution of the LET problem in Theorem 2.10 using $\mathbb {L}=\textrm{L}_1$; thus in this case $\mathsf R=\pi /2$. Using the notations for $\textrm{supp}(\mu _i)=S_i=S'_i+S''_i$ and $\mu _i=\mu '_i+ \mu ''_i$ from Theorem 2.5 we can compare these to the sets $O_i$, $Q_i$, $D'_i$, and $D''_i$ defined for an optimal pair $(\varphi _0,\varphi _1)$ as in Theorem 3.2. So far we constructed optimal pairs $(\varphi _0,\varphi _1)$ satisfying3.23However, following [34, Ch. 5], we will show that it is possible to restrict to “tight optimal pairs” satisfying  and $\varphi _1= \varphi _0^{\textrm{L}_1\rightarrow }$, which implies that $\varphi _0$ is l.s.c. and $\varphi _1$ is u.s.c. This possibility leads to the following refinement of the results in [27, Thm. 6.6(iii)].

### Theorem 3.3

(Regularity of optimal $\textsf{H}\!\!\textsf{K}$ potentials) Let $\mu _0,\mu _1$ be nontrivial measures in $\mathcal {M}({\mathbb {R}^{d}})$ with decompositions given by (2.12)–(2.13).

1. There exists an optimal pair of potentials $\varphi _0,\varphi _1:\mathbb {R}^d\rightarrow [-\infty ,+\infty ]$ with $\varphi _0$ being l.s.c. and $\varphi _1$ u.s.c., solving the dual problem of Theorem 2.12 and 3.24
  3.25 $$\begin{aligned} S_i\subset Q_i, \quad S_0'\subset S^{\pi /2}_1 \subset O_0,\quad S_1'\subset S^{\pi /2}_0 \subset O_1, \end{aligned}$$
  3.26 $$\begin{aligned} \varphi _0=-\infty \text { on } S_0'',\quad \text {and}\quad \varphi _1=+\infty \text { on } S_1'', \end{aligned}$$
  where the sets $O_i$ and $Q_i$ are as in (3.9).
2. If ${\varvec{\eta }}$ is an optimal solution of the LET problem (2.25), the functions $\sigma _0:=\mathrm e^{2\varphi _0}$ and $\sigma _1:=\mathrm e^{-2\varphi _1}$ provide lower semi-continuous representatives of the densities of the marginals $\eta _i = \pi ^i_\sharp {\varvec{\eta }}$ with respect to $\mu _i$, i.e., $\eta _i=\sigma _i\mu _i$, and ${\varvec{\eta }}$ is concentrated on the contact set $M_{\textrm{fin}}$ so that ${\text {supp}}({\varvec{\eta }})\subset M$ (see Theorem 3.2). The marginals $\eta _i$ are concentrated on the open sets $O_i$. Conversely, if $\widetilde{\varvec{\eta }}$ satisfies ${\text {supp}}(\widetilde{\varvec{\eta }})\subset M$ and $\widetilde{\eta }_i=\sigma _i\mu _i$, then $\widetilde{\varvec{\eta }}$ is an optimal solution of the LET problem (2.25).
3. If $\mu _0$ (resp. $\mu _0'$) does not charge $(d{-}1)$-rectifiable sets, e.g. in the case that $\mu _0\ll \mathcal {L}^{d}$ or if $\mu _0({\text {bdry}}_{{\pi /2}}( S_0))=0$ (resp. $\mu _0'({\text {bdry}}_{{\pi /2}}( S_0))=0$), then for every optimal pair $(\varphi _0,\varphi _1)$ with  and $\varphi _1$ u.s.c., the measure $\mu _0$ is concentrated on the open set ${\text {int}}(Q_0)$ (resp. $\mu _0'$ is concentrated on the open set $\Omega _0$).
4. If $\mu _0'$ is concentrated on $D_0'=\textrm{dom}(\nabla \varphi _0)$ (in particular if $\mu _0'\ll \mathcal {L}^{d}$) then the optimal transport plan ${\varvec{\eta }}$ solving the LET formulation is unique, it is concentrated on $D_0'\times S^{\pi /2}_0$, and it is induced by the graph of $\varvec{T}_{0\rightarrow 1}$, i.e. ${\varvec{\eta }}=(\textrm{Id},\varvec{T}_{0\rightarrow 1})_\sharp \eta _0$ with $\varvec{T}_{0\rightarrow 1}$ from Theorem 3.2(6).
5. If $$\mu _0',\mu _1'\ll \mathcal {L}^{d}$$ then $$\mu _0'$$ is concentrated on $$D_0''\cap \varvec{T}_{0\rightarrow 1}^{-1}(D_1'')$$, where $$D_i''=\textrm{dom}(\textrm{D}^2\varphi _i)$$, and $$\varvec{T}_{0\rightarrow 1}$$ is $$\mu _0'$$-essentially injective with $$\det \textrm{D}\varvec{T}_{0\rightarrow 1}>0$$$$\mu _0$$-a.e. in $$D_0''$$.

### Proof

Assertion (1). Let $(\phi _0,\phi _1)$ be an optimal Borel pair according to Theorem 2.14(2), see (2.43), satisfying

3.27 $$\begin{aligned} \phi _i\in \mathbb {R}\ \mu _i\,\text {-a.e. in}\,S_i',\quad \phi _0=-\infty \ \mu _0\,\text {-a.e. in}\,S_0'',\ \phi _1=+\infty \ \mu _1\,\text {-a.e. in}\,S_1''.\nonumber \\ \end{aligned}$$

With this pair, we set , and recalling (3.3) we easily obtain

3.28 $$\begin{aligned} \varphi _0\leqq \phi _0\quad \text {in }{\mathbb {R}^{d}},\quad \phi _1(x_1) \leqq \textrm{L}_1(x_1{-}x_0)+\varphi _0(x_0) \quad \text {if }x_0,x_1\in \mathbb {R}^d,\ |x_1{-}x_0|<\pi /2.\nonumber \\ \end{aligned}$$

Looking at the dual problem (2.34a) with the more general admissible set of Borel pairs as described in (2.36), we see that $(\varphi _0,\phi _1)$ is still optimal.

Repeating the argument, we can set $\varphi _1= \varphi _0^{\textrm{L}_1\rightarrow }$ to find a new optimal pair satisfying $\varphi _1 \geqq \phi _1$. However, exploiting (3.5) we see that the tightness relation (3.24) holds for the optimal pair $(\varphi _0,\varphi _1)$. This fact implies that $\varphi _0$ is l.s.c. and $\varphi _1$ is u.s.c.

By the construction of $\phi _i$ in Theorem 2.14(2) we have

$$\begin{aligned} \{\phi _i \in \mathbb {R}\} = S'_i, \quad \{ \phi _0=-\infty \} = S''_0, \quad \text {and }\ \{ \phi _1=+\infty \} = S''_1. \end{aligned}$$

Together with $\phi _0 \geqq \varphi _0$ and $\phi _1 \leqq \varphi _1$ we find

$$\begin{aligned}&S''_0 = \{ \phi _0=-\infty \} \subset \{ \varphi _0=-\infty \} \ \text { and } \ S_0 = \{ \phi _0<+\infty \} \subset \{ \varphi _0 <+\infty \}=Q_0, \\&S''_1 = \{ \phi _1=+\infty \} \subset \{ \varphi _1=+\infty \} \ \text { and } \ S_1 = \{ \phi _1>-\infty \} \subset \{ \varphi _1>-\infty \} = Q_1. \end{aligned}$$

Clearly, $S'_0 =S_0\cap S^{\pi /2}_1\subset S^{\pi /2}_1$. Moreover, for $x_0 \in S^{\pi /2}_1$ we find $y_1\in S_1$ with $|y_1{-}x_0| <{\pi /2}$, i.e. $\textrm{L}_1(y_1{-}x_0)<\infty$. With this we have  and conclude $x_0 \in O_0$. Thus, $S'_0\subset S^{\pi /2}_1 \subset O_0$ is shown and $S'_1\subset S^{\pi /2}_0 \subset O_1$ follows similarly. Hence, (3.25) and (3.26) are established.

Assertion (2). The claim follows immediately from Theorem 2.14.

Assertion (3). We just consider the case of $\mu _0$, since the argument for $\mu _0'$ is completely analogous and eventually uses the fact that $\Omega _0=O_0\cap {\text {int}}(Q_0)$ and $\mu _0'$ is also concentrated on $O_0$ by (3.25).

By Theorem 3.2 (cf. (3.12)) we know that $\partial Q_0={\text {bdry}}_{{\pi /2}}(Q_0)$. Since $\partial Q_0$ is $(d{-}1)$-rectifiable and $\mu _0$ does not charge $(d{-}1)$-rectifiable sets, we conclude $\mu _0(\partial Q_0)=0$.

If $\mu _0({\text {bdry}}_{{\pi /2}}(S_0))=0$, we also obtain $\mu _0(\partial Q_0)=0$ via the following arguments: By (3.25) we have $S_0\subset Q_0$, which implies that a point $x \in \partial S_0\cap {\text {bdry}}_{{\pi /2}}(Q_0)$ also lies ${\text {bdry}}_{{\pi /2}}(S_0)$. Using $\partial Q_0={\text {bdry}}_{{\pi /2}}(Q_0)$ we obtain $\partial S_0 \cap \partial Q_0 \subset {\text {bdry}}_{{\pi /2}}(S_0)$ and find

$$\begin{aligned} \mu _0(\partial Q_0) \overset{\text {(i)}}{=} \mu _0(\partial Q_0 \cap S_0) \overset{\text {(ii)}}{=} \mu _0(\partial Q_0 \cap \partial S_0) \leqq \mu _0\big ({\text {bdry}}_{{\pi /2}}(S_0)\big )= 0, \end{aligned}$$

where we used $S_0= \textrm{sppt}(\mu _0)$ in $\overset{\text {(i)}}{=}$ and $S_0 \subset Q_0$ in $\overset{\text {(ii)}}{=}$. Thus, we have shown that $\mu _0$ is concentrated on ${\text {int}}(Q_0)$.

Assertion 4. If $\mu _0'\ll \mathcal {L}^{d}$ then $\mu _0'$ is concentrated on $\Omega _0$ by Claim 3 and $\mu _0(\Omega _0\setminus D_0')=0$ by 3.2(6). By the previous claim , we know that the first marginal $\eta _0$ of ${\varvec{\eta }}$ is given by  (in particular $\eta _0(\mathbb {R}^d\setminus D_0')=0$) so that ${\varvec{\eta }}$ is concentrated on $M_{\textrm{fin}}\cap (D_0'\times \mathbb {R}^d)$ which is the graph of the map $\varvec{T}_{0\rightarrow 1}$ given by Theorem 3.2(6).

Assertion 5. Let us first recall that for $i=0,1$ the marginal $\eta _i$ of ${\varvec{\eta }}$ and the measure $\mu _i'$ are mutually absolutely continuous. Since $\mu _i'\ll \mathcal {L}^{d}$ we know by Theorem 3.2(6) and the third claim that $\mu _i'(\mathbb {R}^d{\setminus } D_i'')=\mu _i'(\Omega _i{\setminus } D_i'')=0$, so that $\eta _i(\mathbb {R}^d{\setminus } D_i'')=0$ and $\eta _0(\varvec{T}^{-1}_{0\rightarrow 1}(\mathbb {R}^d{\setminus } D_1''))= \eta _1(\mathbb {R}^d{\setminus } D_1'')=0$; we deduce that $\eta _0$ and $\mu _0'$ are concentrated on $D_0''\cap \varvec{T}^{-1}_{0\rightarrow 1}(D_1'').$

We can apply Theorem 3.2(6), inverting the order of the pair $(\varphi _0,\varphi _1)$ and obtaining that for every $x_1\in D_1'$ there is a unique element $x_0\in \mathbb {R}^d$ in the section $M_{1\rightarrow 0}(x_1)$, i.e. such that $(x_0,x_1)\in M_{\textrm{fin}}$. This result precisely shows that the restriction of $\varvec{T}_{0\rightarrow 1}$ to $D_0'\cap \varvec{T}^{-1}_{0\rightarrow 1}(D_1')\supset D_0''\cap \varvec{T}^{-1}_{0\rightarrow 1}(D_1'')$ is injective. Since $(\varvec{T}_{0\rightarrow 1})_\sharp \eta _0=\eta _1\ll \mathcal {L}^{d}$, we can eventually apply [2, Lemma 5.5.3] which shows that $\det \textrm{D}\varvec{T}_{0\rightarrow 1}>0$ $\mu _0$-a.e. in $D_0''$.                                                                   $\square$

It is important to realize that the tightness condition (3.24) is strictly stronger than the optimality conditions (3.23). However, even for tight optimal pairs there is some freedom outside the supports of the measures $\mu _0$ and $\mu _1$, as is seen in the following simple case.

### Example 3.4

(**Tight optimal pairs for two Diracs**) This example lies in-between Examples 3.1 and 4.5. For two points $z_0,z_1\in \mathbb {R}^d$ with $\varrho =|z_1{-}z_0|= \pi /3$, such that $\cos _{{\pi /2}}\!{(\varrho )}=1/2$. We consider two measures $\mu _i=\delta _{z_i}$. With $s_i=S'_i =\{z_i\}$ we easily find the two optimal potential $(\phi _0,\phi _1)$ according to Theorem 2.14, see (2.43):

$$\begin{aligned} \phi _0(x_0) = {\left\{ \begin{array}{ll}-\frac{\log 2}{2} &{}\text {for } x_0=z_0, \\ +\infty &{} \text {otherwise}, \end{array}\right. } \quad \text {and} \quad \phi _1(x_1) = {\left\{ \begin{array}{ll}\frac{\log 2}{2} &{}\text {for } x_1=z_1, \\ -\infty &{} \text {otherwise}, \end{array}\right. } \end{aligned}$$

In particular, we have $\phi _1(z_1)-\phi _0(z_0)=\log 2 = \textrm{L}_1(z_1{-}z_0)=\frac{1}{2}\ell (\varrho )$.

Proceeding as in Step 1 of the above proof with  and taking into account the calculations of Example 3.1, we obtain a first tight optimal pair

$$\begin{aligned}{} & {} (\varphi ^{(1)}_0,\varphi ^{(1)}_1) \quad \text {with } \varphi ^{(1)}_0(x_0) \\{} & {} \quad = {\left\{ \begin{array}{ll} \frac{\log 2}{2} - \textrm{L}_1(z_1{-}x_0) &{} \text {for } x_0 \in B_{\pi /2}(z_1), \\ -\infty &{} \text {otherwise}, \end{array}\right. } \quad \text {and } \ \varphi ^{(1)}_1= \phi _1. \end{aligned}$$

Interchanging the roles of $\phi _0$ and $\phi _1$ we arrive at a second tight optimal pair

$$\begin{aligned} (\varphi ^{(2)}_0,\varphi ^{(2)}_1) \quad \text {with } \varphi ^{(2)}_0= & {} \phi _0 \quad \text {and } \\ \varphi ^{(2)}_1(x_1)= & {} {\left\{ \begin{array}{ll} -\frac{\log 2}{2} + \textrm{L}_1(x_1{-}z_0) &{} \text {for } x_1 \in B_{\pi /2}(z_0), \\ \infty &{} \text {otherwise}. \end{array}\right. } \end{aligned}$$

A third case is obtained by choosing $z_1^*\ne z_1$ and considering an optimal pair $(\phi _0,\widetilde{\phi }_1)$ with $\phi _0$ from above and

$$\begin{aligned} \widetilde{\phi }_1(x_1)= {\left\{ \begin{array}{ll} \frac{\log 2}{2} &{} \text {for } x_1=z_1, \\ a_1 &{}\text {for } x_1=z_1^*, \\ -\infty &{}\text {otherwise}. \end{array}\right. } \quad \text {where } a_1\leqq - \frac{\log 2}{2} + \textrm{L}_1(z_1^*{-}z_0). \end{aligned}$$

We obtain $\varphi ^{(3)}_0: x_0\mapsto \max \{\frac{\log 2}{2} - \textrm{L}_1(z_1{-}x_0),\, a_1 - \textrm{L}_1(z_1^*{-}x_0) \}$ and the tight optimal pair $\big (\varphi ^{(3)}_0,( \varphi ^{(3)}_0)^{\textrm{L}_1\rightarrow }\big )$.

With the notation of Theorem 3.2 we have $O_0^{(3)}=\{\varphi _0^{(3)} >- \infty \} = B_{\pi /2}(z_1)\cup B_{\pi /2}(z_1^*) = \widetilde{Q}_1^{\pi /2}$, since $\widetilde{Q}_1=\{\widetilde{\phi }_1>-\infty \} = \{z_1,z_1^*\}$, i.e. (3.11) holds. Because of $Q_0^{(3)} = \{\varphi _1^{(3)} <+\infty \}=\mathbb {R}^2$, also (3.12) is true.

The following corollary shows that in the case of an absolutely continuous reduced pair $(\mu _0,\mu _1)$ the density of $\mu _1$ can be written in terms of the optimal pair $(\sigma _0,\sigma _1)$, the transport map $\varvec{T}$, and the density of $\mu _0$, and vice versa:

### Corollary 3.5

(Monge solutions) Let $\mu _0,\mu _1\in \mathcal {M}({\mathbb {R}^{d}})^2$ with $\mu _1''=0$, and let $(\varphi _0,\varphi _1)$ be a tight optimal pair of potentials according to Theorem 3.3. If $\mu _0'$ is concentrated on $D_0'=\textrm{dom}(\nabla \varphi _0)$ (in particular if $\mu _0'\ll \mathcal {L}^{d}$), then there exists a “unique” (up to $\mu _0$-negligible sets) optimal transport-growth pair $(\varvec{T},q)$ attaining the minimum for the Monge Problem 2.9, namely

3.29 $$\begin{aligned} (\varvec{T},q)_\star \mu _0=\mu _1 \quad \text {and } \quad {\mathcal {C}}(q,\varvec{T};\mu _0)=\textsf{H}\!\!\textsf{K}^2(\mu _0,\mu _1). \end{aligned}$$

If $\sigma _i,\varphi _i,D_i',D_i'',{\varvec{\eta }}$, $\varvec{T}_{0\rightarrow 1},\varvec{T}_{1\rightarrow 0}$ are given as in Theorem 3.2 and 3.3, the pair $(\varvec{T},q)$ can be obtained in the following way:

1. The restriction of $\varvec{T}$ to $D_0'$ coincides with the map $\varvec{T}_{0\rightarrow 1}$ (and the plan ${\varvec{\eta }}$) as in Theorem 3.2, whereas $\varvec{T}(x):=x$ for every $x\in \mathbb {R}^d{\setminus } D_0'$ (in particular in $S_0''$).
2. $q(x)\equiv 0$ for $x\in \mathbb {R}^d{\setminus } D_0'$ (in particular in $S_0''$) and
  3.30 $$\begin{aligned} q^2(x) = \frac{\sigma _0(x)}{\sigma _1(\varvec{T}_{0\rightarrow 1}(x))}= \sigma ^2_0(x)+\frac{1}{4}|\nabla \sigma _0(x)|^2 \text { for }x\in D_0'. \end{aligned}$$

Moreover, $$\varvec{T}$$ satisfies

3.31 $$\begin{aligned} |\varvec{T}(x){-}x|<\pi /2\quad \text {and}\quad \sigma _0(x)\sigma _1(\varvec{T}(x))=\cos (|x{-}\varvec{T}(x)|)^2 \quad \text {in } D_0'. \end{aligned}$$

If $\mu _0\ll \mathcal {L}^{d}$, then $\mu _1\ll \mathcal {L}^{d}$ if and only if $\det \textrm{D}\varvec{T}(x)>0$ for $\mu _0$-a.e. $x\in D_0''$. In this case, setting $\mu _i=c_i\mathcal {L}^{d}\ll \mathcal {L}^{d}$ we have

3.32 $$\begin{aligned} c_1=\Big (c_0\frac{q^2}{\det \textrm{D}\varvec{T}}\Big )\circ \varvec{T}^{-1}\quad \mathcal {L}^{d}\text {-a.e. in}\, \varvec{T}(D_0'')\subset \Omega _1. \end{aligned}$$

To obtain the second identity in (3.30), we exploit the first-order optimality (3.15) and $\sigma _0=\textrm{e}^{2\varphi _0}$ giving $\frac{1}{2\sigma _0} \nabla \sigma _0= \nabla \varphi _0(x) = {\textbf {tan}}(x{-} \varvec{T}(x))$ by (3.15). Thus, using the optimality condition (3.31) (coming from (2.42e)) we find

3.33 $$\begin{aligned} q^2(x)=\frac{\sigma _0^2(x)}{\cos ^2(|x{-}\varvec{T}(x)|)} = \sigma _0^2(x)(1 {+}\tan ^2(|x{-}\varvec{T}(x)|))= \sigma _0^2(x)+\frac{1}{4}|\nabla \sigma _0(x)|^2.\nonumber \\ \end{aligned}$$

We can also rephrase the above results in terms of the optimal Kantorovich potential $\xi _0$ in (2.34b). This potential, which satisfies the relations $\xi _0 = \frac{1}{2}(\sigma _0{-}1) = \textsf{G}_1(\varphi _0) = \frac{1}{2}(\textrm{e}^{2\varphi _0}{-}1)$, will be the best choice for characterizing the densities of the Hellinger–Kantorovich geodesic curves. Indeed, the transport map $\varvec{T}$ on $D_0''$ takes the form

3.34 $$\begin{aligned} \begin{aligned} \varvec{T}(x)&= x + \textbf{arctan}\Big (\frac{\nabla \xi _0(x)}{1{+}2\xi _0(x)}\Big )= x + \textbf{arctan}\Big ({\nabla \varphi _0(x)}\Big ), \\ q^2(x)&=(1{+} 2 \xi _0(x))^2+ |\nabla \xi _0(x)|^2. \end{aligned} \end{aligned}$$

If $\mu _0,\mu _1$ have full support $S_0=S_1=\mathbb {R}^d$, then Theorem 3.3 immediately yields $\Omega _i = O_i\cap \textrm{int}(Q_i)=\mathbb {R}^d$, so that $\varphi _0$ and $\varphi _1$ take values in $\mathbb {R}$, are locally Lipschitz, and locally semi-convex and semi-concave, respectively. Another important case where the properties of $\varphi _0,\varphi _1$ can be considerably refined is when $\mu _0,\mu _1$ are *strongly reduced* (cf. Definition 2.6) and have compact support.

### Theorem 3.6

(Improved regularity in case of strongly reduced pairs) Let us assume that the supports $S_0,S_1$ of $\mu _0,\mu _1$ are compact and satisfy $S_i\subset S^{\pi /2}_{1-i}$, so that $\mu _0,\mu _1$ is a strongly reduced pair (cf. Definition 2.6). Then it is possible to find a pair of optimal potentials $\varphi _0,\varphi _1$ as in Theorem 3.3 satisfying the following additional properties:

1. $\varphi _i$ are uniformly bounded (in particular $\Omega _i=\mathbb {R}^d$ and $M=M_{\textrm{fin}}$): there exist constants $\phi _{\textrm{min}}<\phi _{\textrm{max}}\in \mathbb {R}$ such that
  3.35 $$\begin{aligned} \phi _{\textrm{min}}\leqq \varphi _i\leqq \phi _{\textrm{max}}\quad \text {in }\mathbb {R}^d. \end{aligned}$$
2. If $\theta \in [0,\pi /2[$ satisfies $\cos ^2(\theta )=\textrm{e}^{2(\phi _{\textrm{min}}-\phi _{\textrm{max}})}$ then for every $x_0,x_1\in \mathbb {R}^d$
  3.36 $$\begin{aligned} (x_0,x_1)\in M\quad \Rightarrow \quad |x_1{-}x_0|\leqq \theta . \end{aligned}$$
3. $\varphi _i$ are Lipschitz, $\varphi _0$ is semi-convex, $\varphi _1$ is semi-concave.

### Proof

Assertion (1). Let $\varphi _0',\varphi _1'$ be an optimal pair as in Theorem 3.3. Since $\varphi _1'$ is u.s.c and $\varphi _1'<+\infty$ on $S^{\pi /2}_0$, we have $\phi _{\textrm{max}}:=\max _{S_1}\varphi _1'<+\infty$. We can then define $\zeta _1:= \min \{\varphi _1', \phi _{\textrm{max}}\}$ observing that $\zeta _1\leqq \phi _{\textrm{max}}$ in $\mathbb {R}^d$ and $(\varphi _0',\zeta _1)$ is still optimal since $\zeta _1=\varphi _1'$ on $S_1$.

Arguing as in the proof of Theorem 3.3, we define , observing that $\zeta _0\leqq \phi _{\textrm{max}}$ as well. On the other hand, $\zeta _0$ is l.s.c. and $\zeta _0>-\infty$ on $S^{\pi /2}_1 \supset S_0$, so that $\phi _{\textrm{min}}:=\min _{S_0}\zeta _0'>-\infty$. Setting $\zeta _0':= \max \{\zeta _0, \phi _{\textrm{min}}\}$ we obtain a new optimal pair $(\zeta _0',\zeta _1)$ with $\phi _{\textrm{min}}\leqq \zeta _0\leqq \phi _{\textrm{max}}$. Hence, with $\zeta _1':=(\zeta _0')^{\textrm{L}_1\rightarrow }$ we get the desired optimal pair $(\zeta _0',\zeta _1')$ satisfying $\phi _{\textrm{min}}\leqq \zeta _i'\leqq \phi _{\textrm{max}}$ as well.

Assertion (2). This assertion is now an easy consequence of the definition of contact set (3.13) and the fact that $\varphi _1(x_1)-\varphi _0(x_0)\leqq \phi _{\textrm{max}}-\phi _{\textrm{min}}$. Assertion (3). The last assertion follows as Theorem 3.25. $\quad \square$

## Dynamic Duality and Regularity Properties of the Hamilton–Jacobi Equation

In the previous section, the regularity properties of the optimal $\textsf{H}\!\!\textsf{K}$ pairs $(\varphi _0,\varphi _1)$ were studied, which can be understood via the static formulations of $\textsf{H}\!\!\textsf{K}$ as only the measures $\mu _0$ and $\mu _1$ are involved. Now, we consider the dual potentials $\xi _t(x)=\xi (t,x)$ along geodesics $(\mu _t)_{t\in [0,1]}$. At this stage, the present Section 4 is completely independent of the previous Section 3. Only in the upcoming Section 5, we will combine the two results to derive the finer regularity properties of the geodesics $\mu _t$.

In [27, Sect. 8.4], it is shown that the optimal dual potentials $\xi$ in the dynamic formulation in (2.3) (but now for $\alpha =1$ and $\beta =4$) are subsolutions to a suitable Hamilton–Jacobi equation, namely,

4.1 $$\begin{aligned} \begin{aligned} \frac{1}{2\tau } \textsf{H}\!\!\textsf{K}(\mu _0,\mu _\tau )^2= \sup \bigg \{\int _{\mathbb {R}^{d}}&\xi (\tau ,\cdot )\;\!\textrm{d}\mu _\tau -\int _{\mathbb {R}^{d}}\xi (0,\cdot )\;\!\textrm{d}\mu _0\,\Big |\, \xi \in \textrm{C}^\infty _\textrm{c}([0,\tau ]\times \mathbb {R}^d), \\&\frac{\partial }{\partial t}\xi + \frac{1}{2} |\nabla \xi |^2+2 \xi ^2\leqq 0 \quad \text {in }[0,\tau ]\times \mathbb {R}^d\bigg \}. \end{aligned} \end{aligned}$$

Theorem 8.11 in [27] shows that the maximal subsolutions of the generalized Hamilton–Jacobi equation (2.4) for $t\in (0,\tau )$ are given by the following *generalized Hopf–Lax formula*

4.2 $$\begin{aligned} \xi _t(x)=\xi (t,x) = \big ({\mathscr {P}}_{\hspace{-2.0pt}t}\hspace{1.0pt} \xi _0\big )(x)= \frac{1}{t} {\mathscr {P}}_{\hspace{-2.0pt}1}\hspace{1.0pt}\big (t\xi _0(\cdot )\big )(x)=\inf _{y\in {\mathbb {R}^{d}}} \frac{1}{2t} \Big (1-\frac{\cos ^2_{{\pi /2}}\!{\left( |x{-}y|\right) }}{1+ 2t\xi _0(y)}\Big ),\nonumber \\ \end{aligned}$$

where $\xi _0\in \textrm{C}^1({\mathbb {R}^{d}})$ is fixed and such that $\inf _{{\mathbb {R}^{d}}}\xi _0(\cdot )>-\frac{1}{2\tau }$, compare with (1.13).

In the spirit of the previous section, it is possible to derive some semi-concavity properties of $\xi _t$ from this formula. However, these are not enough as we need more precise second order differentiability. To obtain the latter, we use the fact that a geodesic curve is not oriented, meaning that $t \mapsto \mu _{1-t}$ is still a geodesic, or in other words that $t\mapsto \xi _{1-t}$ has to also solve a Hamilton–Jacobi equation. Thus, our strategy will be the following: For an optimal pair $(\xi _0,{\bar{\xi }}_1)$ in (2.34b), we construct a forward solution $\xi _t$ starting from $\xi _0$ and backward solutions starting from ${\bar{\xi }}_1$ via

4.3 $$\begin{aligned} \xi _t = {\mathscr {P}}_{\hspace{-2.0pt}t}\hspace{1.0pt} \xi _0 \text { for }t\in (0,1]\quad \text {and} \quad {\bar{\xi }}_t = {\mathscr {R}}_{\hspace{-2.0pt}t}\hspace{1.0pt}\bar{\xi }_1:=- {\mathscr {P}}_{\hspace{-2.0pt}t}\hspace{1.0pt}({-}{\bar{\xi }}_1) \text { for } t\in [0,1). \end{aligned}$$

In Section 5, optimality will be used to guarantee that $\xi _t$ and ${\bar{\xi }}_t$ are essentially the same so that semi-concavity of $\xi _t$ and semi-convexity of ${\bar{\xi }}_t$ provide the desired smoothness.

### Exploiting the Generalized Hopf–Lax Formula for Regularity

In this section, we study in detail the regularity properties of the function $\xi _t$ arising in (4.2). Assuming that $\inf _{x\in {\mathbb {R}^{d}}}\xi _0(x)\geqq - \frac{1}{2\tau }$ we see that ${\mathscr {P}}_t\xi _0$ is well-defined for $t\in (0,\tau )$ and can be equivalently characterized by

4.4 $$\begin{aligned} \big ({\mathscr {P}}_{\hspace{-2.0pt}t}\hspace{1.0pt} \xi _0\big )(x)&= \inf _{y \in B_{\pi /2}(x)} \frac{1}{2t} \Big (1- \frac{\cos ^2_{{\pi /2}}\!{\left( |x{-}y|\right) }}{1+ 2t\xi _0(y)}\Big ). \end{aligned}$$

We can extend (4.4) at $t=\tau$ if we define the quotients $a/0:=+\infty$, $a/(+\infty ):=0$ for every $a>0$. Moreover, since $t\mapsto {\mathscr {P}}_{\hspace{-2.0pt}t}\hspace{1.0pt}\xi _0(x)$ is decreasing, we easily get

4.5 $$\begin{aligned} \xi _t(x)=\big ({\mathscr {P}}_{\hspace{-2.0pt}t}\hspace{1.0pt} \xi _0\big )(x)=\lim _{s\uparrow t } \big ({\mathscr {P}}_{\hspace{-2.0pt}s}\hspace{1.0pt}\xi _0\big )(x)\quad \text {for every }x\in {\mathbb {R}^{d}},\ t\in (0,\tau ] \end{aligned}$$

so that many properties concerning the limiting case $t=\tau$ can be easily derived by continuity as $t\uparrow \tau$.

If $\xi _0$ is l.s.c. and $\big ({\mathscr {P}}_{\hspace{-2.0pt}t}\hspace{1.0pt} \xi _0\big )(x)<\frac{1}{2t}$, the infimum in (4.4) it attained at a compact set denoted by

4.6 $$\begin{aligned} \textsf{M}_t\xi _0(x):={\text {argmin}}_y \frac{1}{2t} \Big (1-\frac{\cos ^2_{{\pi /2}}\!{\left( |x{-}y|\right) }}{1+ 2t\xi _0(y)}\Big )\subset {B_{{\pi /2}}(x)}. \end{aligned}$$

Notice that $\big ({\mathscr {P}}_{\hspace{-2.0pt}t}\hspace{1.0pt} \xi _0\big )(x)=\frac{1}{2t}$ only if $\xi _0$ is identically $+\infty$ in $B_{{\pi /2}}(x)$ and in this case any element of $\overline{B_{{\pi /2}}(x)}$ is a minimizer. For later usage we also define $\textsf{M}_0\xi (x)=\{x\}$.

We also observe that if $\xi _0(x)=a$ is constant then ${\mathscr {P}}_{\hspace{-2.0pt}t}\hspace{1.0pt}\xi _0$ is constant in *x*, namely

4.7 $$\begin{aligned} {\mathscr {P}}_{\hspace{-2.0pt}t}\hspace{1.0pt}\xi _0(x)= {\mathscr {P}}_{\hspace{-2.0pt}t}\hspace{1.0pt}a (x)=P_a(t):=\frac{a}{1+2at}, \quad \text {with }P_{\infty }(t):=\frac{1}{2t}. \end{aligned}$$

A crucial property of (4.2) is the link with the classical Hopf–Lax formula on the cone $\mathfrak {C}$ for a function $\zeta :\mathfrak {C}\rightarrow \mathbb {R}$ satisfying $\zeta ([x,r])\geqq -\frac{1}{2\tau } r^2$. For $t\in (0,\tau )$ the Hopf–Lax formula on $\mathfrak {C}$ reads as

4.8 $$\begin{aligned} {\mathscr {Q}}_t\zeta ([x,r]):=\inf _{[x',r'] \in \mathfrak {C}}\zeta ([x',r'])+\frac{1}{2t}\textsf{d}^2_\mathfrak {C}\big ([x,r],[x',r']\big ). \end{aligned}$$

For $\xi _0$ satisfying $\xi _0\geqq -\frac{1}{2\tau }$ and $t\in (0,\tau )$ we set $\zeta ([x,r]):= \xi _0(x)r^2$ and find (cf. [27, Thm. 8.11])

4.9 $$\begin{aligned} \xi _t={\mathscr {P}}_{\hspace{-2.0pt}t}\hspace{1.0pt}\xi _0\quad \Longleftrightarrow \quad \xi _t(x)r^2={\mathscr {Q}}_t\zeta ([x,r])\quad \text {for all } x\in \mathbb {R}^d; \end{aligned}$$

Moreover, if $\xi _0$ is lower semi-continuous the infimum in (4.8) is attained and we have

4.10 $$\begin{aligned} \xi _t(x)r^2{} & {} =\zeta ([x',r']) {+} \frac{1}{2t} \textsf{d}^2_\mathfrak {C}\big ([x,r],[x',r']\big )\nonumber \\{} & {} \Longleftrightarrow {\left\{ \begin{array}{ll} x'\in \textsf{M}_t\xi _0(x) \text { and}\\ (1{+}2t\xi _0(x')) (r')^2=(1{-}2t\xi _t(x))r^2 \end{array}\right. } \end{aligned}$$

(where $[x',r']=\mathfrak {o}$ if $r'=0$, corresponding to the case $1{-}2t\xi _t(x)=0$). From (4.8) and (4.9) we also deduce the estimate

4.11 $$\begin{aligned} \big (1{-} 2 t \xi _t(x)\big )r^2+ \big ( 1{+} 2 t\xi _0(x')\big )(r')^2 \geqq 2rr'\cos _{{\pi /2}}\!{(|x{-}x'|)} \end{aligned}$$

for every $x,x'\in \mathbb {R}^d$ and $r,r'\geqq 0$. Optimizing with respect to $r,r'$ we find

4.12 $$\begin{aligned} \big (1{-} 2 t \xi _t(x)\big )\big ( 1{+} 2 t \xi _0(x')\big ) \geqq \cos ^2_{{\pi /2}}\!{\left( |x{-}x'|\right) } \quad \text {for every }x,x'\in \mathbb {R}^d \end{aligned}$$

and arrive at the following characterization: For all $x\in \mathbb {R}^d$ with $1{-}2t\xi _t(x)>0$ we have

4.13 $$\begin{aligned} x'\in \textsf{M}_t\xi _0(x)\quad \Longleftrightarrow \quad \big (1{-} 2 t \xi _t(x)\big )\big ( 1{+}2t \xi _0(x')\big ) = \cos ^2_{{\pi /2}}\!{\left( |x{-}x'|\right) }. \end{aligned}$$

To treat the factor of *r* and $r'$ in (4.11) efficiently, we define the function

4.14 $$\begin{aligned} Z_t(u',u):=\frac{1{-}2t u}{1{+}2t u'}\ \text { for }1{+}2tu',\ 1{-}2tu\geqq 0 \quad \text {and } Z_t(+\infty ,u)\equiv 0.\qquad \end{aligned}$$

Using (4.13), the optimal $r'$ in (4.10) can now be equivalently characterized by

4.15 $$\begin{aligned} (r')^2=Z_t(\xi _0(x'),\xi _t(x))=\frac{(1{-}2t\xi _t(x))^2}{\cos ^2_{{\pi /2}}\!{\left( |x{-}x'|\right) }}= (1{-}2t\xi _t(x))^2(1+\tan ^2(|x{-}x'|)).\nonumber \\ \end{aligned}$$

The following result collects the properties of ${\mathscr {P}}_{\hspace{-2.0pt}t}\hspace{1.0pt}$ that will be needed in the sequel:

#### Proposition 4.1

(Properties of the generalized Hopf–Lax operator ${\mathscr {P}}_{\hspace{-2.0pt}t}\hspace{1.0pt}$) Let $\xi _0:{\mathbb {R}^{d}}\rightarrow [a,b]$ with $-1/2\leqq a\leqq b\leqq +\infty$ be lower semi-continuous and set $\xi _t:={\mathscr {P}}_{\hspace{-2.0pt}t}\hspace{1.0pt} \xi _0$ for $t\in [0,1]$.

1. *Lower/upper bounds.* The functions $\xi _t$ are well defined and satisfy (cf. (4.7) for $P_a$)
  4.16 $$\begin{aligned} -\frac{1}{2(1{-}t)}\leqq P_a(t)\leqq \xi _t\leqq P_b(t)\leqq \frac{1}{2t}\quad \text {for every }t\in (0,1),\ x\in \mathbb {R}^d.\nonumber \\ \end{aligned}$$
  Moreover, it holds that
  4.17 $$\begin{aligned} \xi _0(x)=-1/2\quad \Leftrightarrow \quad \xi _t(x)=-\frac{1}{2(1{-}t)}. \end{aligned}$$
2. *Semi-concavity.* Setting $\Lambda _a(t):=\frac{1}{t(1+2at)}\leqq \frac{1}{t(1{-}t)}$ the functions $\xi _t$ are $\Lambda _a(t)$-Lipschitz and $\Lambda _a(t)$ semi-concave, i.e. $x\mapsto \xi _t(x)-\frac{\Lambda _a(t)}{2}|x|^2$ is concave.
3. *Semigroup property.* For every $0\leqq s<t\leqq 1$ we have
  4.18 $$\begin{aligned} \xi _t={\mathscr {P}}_{\hspace{-2.0pt}t-s}\hspace{1.0pt}\xi _s \end{aligned}$$
4. *Concatenation of optimal points.* For *s*, *t* with $0\leqq s<t< 1$ and $x\in \mathbb {R}^d$ we define the set-valued function $\textsf{M}_{t\rightarrow s}$ via $\textsf{M}_{t\rightarrow s}(x):= \textsf{M}_{t-s}\xi _s(x)$. For all $0\leqq t_0<t_1<t_2<1$ and all $x_0,x_1,x_2 \in \mathbb {R}^d$ we have:
  4.19 $$\begin{aligned}{} & {} \text {If } x_{1}\in \textsf{M}_{t_2\rightarrow t_1}(x_2) \text { and } x_{0}\in \textsf{M}_{t_1\rightarrow t_0}(x_{1}), \quad \text {then } x_{0}\in \textsf{M}_{t_2 \rightarrow t_0}(x_2) \text { and} \nonumber \\{} & {} Z_{t_2-t_0}(\xi _{t_0}(x_0),\xi _{t_2}(x_2))=Z_{t_1-t_0}(\xi _{t_0}(x_{0}), \xi _{t_1}(x_{1}))\, Z_{t_2-t_1}(\xi _{t_1}(x_{1}),\xi _{t_2}(x_2)).\nonumber \\ \end{aligned}$$
5. *Geodesics on* $\mathfrak {C}$. If $0\leqq t_0<t_1<t_2<1$, $x_0\in \textsf{M}_{t_2\rightarrow t_0}(x_2)$, $r_0=Z_{t_2-t_0}(\xi _{t_0}(x_0),\xi _{t_2}(x))r_2$, and $[x_1,r_1]=\textrm{geo}_{\theta }\big ([x_0,r_0],[x_2,r_2]\big )$ for $\theta =\frac{t_1-t_0}{t_2-t_0}$, then $x_1\in \textsf{M}_{t_2\rightarrow t_1}(x_2).$
6. *Characterization of optimality.* For all $x,y\in \mathbb {R}^d$ and $0\leqq s<t< 1$ with $\tau :=t{-}s$ we have
  4.20 $$\begin{aligned}{} & {} (1{-}2\tau \xi _t(x))(1{+}2\tau \xi _s(y))\geqq \cos ^2_{{\pi /2}}\!{\left( |x{-}y|\right) }, \end{aligned}$$
  4.21 $$\begin{aligned}{} & {} y\in \textsf{M}_{t\rightarrow s}(x),\ \xi _t(x)<\frac{1}{2\tau }\ \Leftrightarrow \ (1{-}2\tau \xi _t(x))(1{+}2\tau \xi _s(y))= \cos ^2_{{\pi /2}}\!{\left( |x{-}y|\right) }. \nonumber \\ \end{aligned}$$

#### Proof

Assertion 1. The first assertion follows by the monotonicity property of ${\mathscr {P}}_t$ and (4.7). Note that (4.17) is a simple consequence of the property

$$\begin{aligned} \frac{1}{2t} \Big (1-\frac{\cos ^2_{{\pi /2}}\!{\left( |x{-}y|\right) }}{1+ 2t\xi (y)}\Big )\geqq -\frac{1}{2(1{-}t)} \end{aligned}$$

with equality if and only if $x=y$ and $\xi (y)=-1/2$.

Assertion 2. It is sufficient to observe that for every $y\in \mathbb {R}^d$

4.22 $$\begin{aligned} x\mapsto \cos ^2_{{\pi /2}}\!{\left( |x{-}y|\right) } \text { is}\, 2\,\text {-Lipschitz,} \quad x\mapsto \cos ^2_{{\pi /2}}\!{\left( |x{-}y|\right) }-|x|^2 \text { is concave},\nonumber \\ \end{aligned}$$

so that

4.23 $$\begin{aligned} x\mapsto \frac{1}{2t}\Big (1-\frac{\cos ^2_{{\pi /2}}\!{\left( |x{-}y|\right) }}{1+2t\xi _0(y)}\Big )\quad \text {is}\, \Lambda _a(t)\,\text {-Lipschitz} \end{aligned}$$

and

4.24 $$\begin{aligned} x\mapsto \frac{1}{2t}\Big (1- \frac{\cos ^2_{{\pi /2}}\!{\left( |x{-}y|\right) }}{1+2t\xi _0(y)}\Big ) -\frac{\Lambda _a(t)}{2}|x|^2\quad \text {is concave}. \end{aligned}$$

Assertion 3. If $t<1$ the semigroup property for ${\mathscr {P}}_t$ can be derived by the link with the Hopf-Lax semigroup in $\mathfrak {C}$ given by (4.9) and the fact that $(\mathfrak {C},\textsf{d}_{\pi ,\mathfrak {C}})$ is a geodesic space. The case $t=1$ follows by approximation and (4.5).

Assertion 4. We set $\tau _0:=t_1-t_0$, $\tau _1:=t_2-t_1$, $r>0$,

$$\begin{aligned} r_1=Z_{\tau _1}(\xi _{t_1}(x_1),\xi _{t_2}(x))r,\quad r_0=Z_{\tau _0}(\xi _{t_0}(x_0),\xi _{t_1}(x_1))r_1 \end{aligned}$$

and use (4.10) and (4.18):

$$\begin{aligned} \xi _{t_2}(x)r^2&= \xi _{t_1}r_1^2+\frac{1}{2\tau _1}\textsf{d}_{\pi ,\mathfrak {C}}^2 ([x_1,r_1], [x,r]) \\&= \xi _{t_0}r_0^2+ \frac{1}{2\tau _0}\textsf{d}_{\pi ,\mathfrak {C}}^2 ([x_0,r_0], [x_1,r_1]) +\frac{1}{2\tau _1}\textsf{d}_{\pi ,\mathfrak {C}}^2 ([x_1,r_1], [x,r]). \end{aligned}$$

On the other hand,

4.25 $$\begin{aligned} \xi _{t_2}(x)r^2\leqq \xi _{t_0}r_0^2+ \frac{1}{2\tau } \textsf{d}_{\pi ,\mathfrak {C}}^2 ([x_0,r_0], [x,r]), \end{aligned}$$

so that we obtain

4.26 $$\begin{aligned} \frac{1}{2\tau _0}\textsf{d}_{\pi ,\mathfrak {C}}^2 ([x_0,r_0], [x_1,r_1]) +\frac{1}{2\tau _1}\textsf{d}_{\pi ,\mathfrak {C}}^2 ([x_1,r_1], [x,r])\leqq \frac{1}{2\tau } \textsf{d}_{\pi ,\mathfrak {C}}^2 ([x_0,r_0], [x,r]);\nonumber \\ \end{aligned}$$

since $\tau =\tau _0+\tau _1$ the opposite inequality always hold in (4.26), and we deduce the equality, which implies that the equality holds in (4.25) as well, showing (4.19) thanks to (4.10).

Assertion 5. We can argue as in the previous assertion, starting from the characterization of $x_0,r_0$

4.27 $$\begin{aligned} \xi _{t_2}(x)r^2= \xi _{t_0}r_0^2+ \frac{1}{2\tau } \textsf{d}_{\pi ,\mathfrak {C}}^2 ([x_0,r_0], [x,r]) \end{aligned}$$

and using the identity along the geodesic in $\mathfrak {C}$ connecting $[x_0,r_0]$ to [*x*, *r*], namely

4.28 $$\begin{aligned} \frac{1}{2\tau _0}\textsf{d}_{\pi ,\mathfrak {C}}^2 ([x_0,r_0], [x_1,r_1]) +\frac{1}{2\tau _1}\textsf{d}_{\pi ,\mathfrak {C}}^2 ([x_1,r_1], [x,r])= \frac{1}{2\tau } \textsf{d}_{\pi ,\mathfrak {C}}^2 ([x_0,r_0], [x,r]).\nonumber \\ \end{aligned}$$

Assertion 6. The final assertion follows from (4.12) and (4.13). $\square$

### Backward Generalized Hopf–Lax Flow and Contact Sets

Let us now consider the backward version of the generalized Hopf–Lax semigroup. By the simple structure of the generalized Hamilton–Jacobi equation (2.4), we immediately see that time reversal leads to the same effect as the sign reversal $\xi \leadsto -\xi$. Hence, the backward semigroup ${\mathscr {R}}_{\hspace{-2.0pt}t}\hspace{1.0pt}$ is defined for ${\bar{\xi }}$ with ${\bar{\xi }} \leqq 1/(2\tau )$ via

4.29 $$\begin{aligned} {\mathscr {R}}_{\hspace{-2.0pt}t}\hspace{1.0pt}\bar{\xi }:=-{\mathscr {P}}_{\hspace{-2.0pt}t}\hspace{1.0pt}(-{\bar{\xi }}) \quad \text {for } t\in (0,\tau ]. \end{aligned}$$

The corresponding properties of ${\mathscr {R}}_{\hspace{-2.0pt}t}\hspace{1.0pt}$ follow easily from Proposition 4.1, but observe that we use ${\bar{\xi }}_t = {\mathscr {R}}_{\hspace{-2.0pt}1-t}\hspace{1.0pt} {\bar{\xi }}_1$ to go backward in time.

#### Corollary 4.2

(Properties of ${\mathscr {R}}_{\hspace{-2.0pt}t}\hspace{1.0pt}$) Let ${\bar{\xi }}_1:{\mathbb {R}^{d}}\rightarrow [-{\bar{b}},-{\bar{a}}]$ with $-\infty \leqq -{\bar{b}}\leqq -{\bar{a}}\leqq 1/2$ be upper semi-continuous and set

4.30 $$\begin{aligned} {\bar{\xi }}_t:={\mathscr {R}}_{\hspace{-2.0pt}1-t}\hspace{1.0pt}{\bar{\xi }}_1\quad \text {for } t\in [0,1]. \end{aligned}$$

1. *Lower/upper bounds.* The functions ${\bar{\xi }}_t$ are well-defined and satisfy
  4.31 $$\begin{aligned} -\frac{1}{2t}\leqq P_{\,{\bar{b}}}(1{-}t)\leqq {\bar{\xi }}_t\leqq P_{{\bar{a}}}(1{-}t) \leqq \frac{1}{2(1{-}t)}\quad \text {for all }t\in (0,1),\ x\in \mathbb {R}^d.\nonumber \\ \end{aligned}$$
  Moreover, we have the equivalence
  4.32 $$\begin{aligned} {\bar{\xi }}_1(x)=1/2\quad \Leftrightarrow \quad {\bar{\xi }}_t(x)=\frac{1}{2t}. \end{aligned}$$
2. *Semi-convexity.* The functions ${\bar{\xi }}_t$ are $\Lambda _{{\bar{a}}}(1{-}t)$-Lipschitz and $\Lambda _{{\bar{a}}}(1{-}t)$ semi-convex, i.e. $x\mapsto {\bar{\xi }}_t(x)+\frac{\Lambda _{{\bar{a}}}(1{-}t)}{2}|x|^2$ is convex (cf. Proposition 4.12 for $\Lambda _a$).
3. *Time-reversed semigroup property.* For every $0\leqq s<t\leqq 1$ we have
  4.33 $$\begin{aligned} {\bar{\xi }}_s={\mathscr {R}}_{\hspace{-2.0pt}t-s}\hspace{1.0pt}\bar{\xi }_t. \end{aligned}$$
4. *Concatenation of optimal points.* Setting  for every $0< s<t\leqq 1$ and $x\in \mathbb {R}^d$, the set-valued function  satisfies the concatenation property for $0<t_0<t_1<t_2\leqq 1$ and $x_0,x_1,x_2\in \mathbb {R}^d$: 4.34
5. *Characterization of optimality.* For all $x,y\in \mathbb {R}^d$ and $0< s<t\leqq 1$ with $\tau :=t-s$
  4.35 $$\begin{aligned}&\displaystyle (1{-}2\tau {\bar{\xi }}_t(x))\,(1{+}2\tau {\bar{\xi }}_s(y)) \geqq \cos ^2_{{\pi /2}}\!{\left( |x{-}y|\right) }, \end{aligned}$$
  4.36 $$\begin{aligned}&\displaystyle x\in \textsf{M}_{s\rightarrow t}(y),\ {\bar{\xi }}_s(y)>-\frac{1}{2\tau }\ \Leftrightarrow \ (1{-}2\tau {\bar{\xi }}_t(x))\,(1{+}2\tau {\bar{\xi }}_s(y))= \cos ^2_{{\pi /2}}\!{\left( |x{-}y|\right) }.\nonumber \\ \end{aligned}$$

#### Proof

We just observe that the second statement in (4.34) follows by the corresponding statement in (4.19) which now reads as

4.37 $$\begin{aligned} Z_{t_2-t_0}(-{\bar{\xi }}_{t_2}(x_2),-{\bar{\xi }}_{t_0}(x_0))= Z_{t_1-t_0}(-\xi _{t_1}(x_{1}),-\xi _{t_0}(x_0) ) \cdot Z_{t_2-t_1}(-{\bar{\xi }}_{t_2}(x_2),-{\bar{\xi }}_{t_1}(x_{1})),\nonumber \\ \end{aligned}$$

and the property $Z_{\tau }(-u',-u) =Z_{-\tau }(u',u) =Z^{-1}_\tau (u,u')$. Equations (4.35) and (4.36) follow by (4.20) and (4.21) changing $\xi _s(y)$ with $-{\bar{\xi }}_t(x)$ and $\xi _t(x)$ with $-{\bar{\xi }}_s(y)$. $\quad \square$

We are now in the position to compare the forward solution $\xi _t$ and the backward solution ${\bar{\xi }}_t$. The main philosophy is that in general we only have ${\mathscr {R}}_{\hspace{-2.0pt}t}\hspace{1.0pt} {\mathscr {P}}_{\hspace{-2.0pt}t}\hspace{1.0pt}\xi _0 \leqq \xi _0$ (cf. (4.38) below), but equality holds $\mu _t$-a.e. if $(\xi _0,{\mathscr {P}}_{\hspace{-2.0pt}1}\hspace{1.0pt}\xi _0)$ is an optimal pair. In the following result, we still stay in the general case comparing arbitrary forward solutions $\xi _t={\mathscr {P}}_{\hspace{-2.0pt}t}\hspace{1.0pt}\xi _0$ and backward solutions ${\bar{\xi }}_t={\mathscr {R}}_{\hspace{-2.0pt}1-t}\hspace{1.0pt}\bar{\xi }_1$ only assuming $\xi _1 \geqq {\bar{\xi }}_1$. Along the contact set $\Xi _t$ where $\xi _t$ and ${\bar{\xi }}_t$ coincide, we can then derive differentiability and optimality properties of $\xi _t$ and ${\bar{\xi }}_t$.

#### Theorem 4.3

(Contact set $\Xi _t$) Let $\xi _0:{\mathbb {R}^{d}}\rightarrow [a,+\infty ]$ be l.s.c. with $a\geqq -1/2$ and ${\bar{\xi }}_1:{\mathbb {R}^{d}}\rightarrow [-\infty , -{\bar{a}}]$ u.s.c. with ${\bar{a}} \leqq 1/2$. Assume ${\mathscr {P}}_{\hspace{-2.0pt}1}\hspace{1.0pt}\xi _0 \geqq {\bar{\xi }}_1$ and set

4.38 $$\begin{aligned} \xi _t:={\mathscr {P}}_{\hspace{-2.0pt}t}\hspace{1.0pt} \xi _0 \ \text { and } \ {\bar{\xi }}_t:={\mathscr {R}}_{\hspace{-2.0pt}1-t}\hspace{1.0pt}{\bar{\xi }}_1\quad \text {for } t\in [0,1]. \end{aligned}$$

Then, the following assertions hold:

1. For every $t\in [0,1]$ we have $\xi _t\geqq {\bar{\xi }}_t$ and the *contact set*
  4.39 $$\begin{aligned} \Xi _t:=\big \{x\in \mathbb {R}^d:{\bar{\xi }}_t(t)=\xi _t(x)\big \} \quad \text {is closed}. \end{aligned}$$
2. For every $t\in (0,1)$ and $x\in \Xi _t$ there exists a unique $p=\varvec{g}_t(x)$ satisfying
  4.40 $$\begin{aligned} \xi _t(y)-\xi _t(x)-\frac{1}{2}\Lambda _{{\bar{a}}}(1{-}t)|x{-}y|^2\leqq \langle p,y-x\rangle \leqq {\bar{\xi }}_t(y)- {\bar{\xi }}_t (x)+\frac{1}{2}\Lambda _{a}(t)|x{-}y|^2\nonumber \\ \end{aligned}$$
  so that in particular $\xi _t$ and ${\bar{\xi }}_t$ are differentiable at *x* with gradient $\varvec{g}_t(x)$ (cf. Proposition 4.12 for $\Lambda _a$).
3. The map $x\mapsto \varvec{g}_t(x)$ is bounded and *C*(*t*)-Lipschitz with $C(t)\leqq 2(\Lambda _a(t)+\Lambda _{{\bar{a}}}(1{-}t))\leqq \frac{4}{t(1{-}t)}$ on $\Xi _t$. Moreover, the sets
  4.41 $$\begin{aligned} \begin{aligned} \Xi ^-_t:={}&\Big \{\, x\in \mathbb {R}^d \; \Big | \; \xi _0=-\frac{1}{2} \,\Big \} = \Big \{\, x\in \Xi _t \; \Big | \; \xi _t=\frac{-1}{2(1{-}t)} \,\Big \} \\ \Xi ^+_t:={}&\Big \{\, x\in \mathbb {R}^d \; \Big | \; {\bar{\xi }}_1=\frac{1}{2} \,\Big \} = \Big \{\, x\in \Xi _t \; \Big | \; {\bar{\xi }}_t=\frac{1}{2t} \,\Big \} \end{aligned} \end{aligned}$$
  are independent of *t*, are contained in $\Xi _t$ for every $t\in [0,1]$, and the critical set $\Xi ^0_t:=\{x\in \Xi _t:\varvec{g}_t(x)=0\}$ of $\varvec{g}_t$ contains $\Xi ^\pm _t$:
  4.42 $$\begin{aligned} \Xi ^0_t\supset \Xi ^-_t\cup \Xi ^+_t \quad \text {for every }t\in (0,1). \end{aligned}$$
4. Let $s\in (0,1)$, $t\in [0,1]$, and $\tau :=t-s\ne 0$. Then, for every $x_s\in \Xi _s$ with $1{+}2\tau \xi _s(x_s)>0$ the set $\textsf{M}_{s\rightarrow t}(x_s)$ consists of a unique element $x_t=:\varvec{T}_{s\rightarrow t}(x_s)$ satisfying 4.43
5. For every $x\in \Xi ^0_s\supset \Xi _s^{\pm }$ we have $\varvec{T}_{s\rightarrow t}(x)=x$ (and thus we set $\varvec{T}_{s\rightarrow t}(x):=x$ also for $t=0$ or $t=1$). Let $s\in (0,1)$ and define $\varvec{T}_{s\rightarrow s}(x)=x$, then for all $x\in \Xi _s$ the mappings $t\mapsto \varvec{T}_{s\rightarrow t}(x)$ are analytic in [0, 1]. For $s,t\in (0,1)$ the mappings $\varvec{T}_{s\rightarrow t}:\Xi _s \rightarrow \mathbb {R}^d$ are Lipschitz. If $t=0$ (resp. $t=1$) then $\varvec{T}_{s\rightarrow t}$ is locally Lipschitz in $\Xi _s\setminus \Xi _s^+$ (resp. in $\Xi _s{\setminus } \Xi _s^-$).
6. Setting
  4.44 $$\begin{aligned} q^2_{s\rightarrow t}(x):=\frac{1+2\tau \xi _s(x)}{ 1 {-}2\tau \xi _t (\varvec{T}_{s\rightarrow t}(x))}= (1 {+} 2\tau \xi _s(x))^2+\tau ^2 |\varvec{g}_s(x)|^2 \end{aligned}$$
  for every $x\in \Xi _s$, the map $t\mapsto q_{s\rightarrow t}(x)$ is analytic in [0, 1], $q_{t\rightarrow s}$ is bounded and Lipschitz with respect to *x*, and $q_{s\rightarrow t}(x)>0$ for $t\in (0,1)$ or $t=0$ and $x\not \in \Xi _s^+$ (resp. $t=1$ and $x\not \in \Xi _s^-$). Moreover, $q_{s\rightarrow t}(x)=1+2(t{-}s)\xi _s(x)\text { for }x\in \Xi _s^\pm$.
7. For all $t_0, t_1\in (0,1)$, $t_2\in [0,1]$, the maps $\varvec{T}_{t_i\rightarrow t_j}$ are Lipschitz on $\Xi _{t_i}$ for $i\in \{0,1\}$, and we have
  4.45 $$\begin{aligned} \varvec{T}_{t_1\rightarrow t_2}\circ \varvec{T}_{t_0\rightarrow t_1}=\varvec{T}_{t_0\rightarrow t_2},\quad q_{t_1\rightarrow t_2}(\varvec{T}_{t_0\rightarrow t_1}(x))\cdot q_{t_0\rightarrow t_1}(x)=q_{t_0\rightarrow t_2}(x).\nonumber \\ \end{aligned}$$

#### Proof

Assertion 1. The inequality

4.46 $$\begin{aligned} \xi _s\geqq {\mathscr {R}}_{\hspace{-2.0pt}t-s}\hspace{1.0pt}\Big ({\mathscr {P}}_{\hspace{-2.0pt}t-s}\hspace{1.0pt}\xi _s\Big ) = {\mathscr {R}}_{\hspace{-2.0pt}t-s}\hspace{1.0pt}\xi _t\quad \text {for } 0<s<t<1 \end{aligned}$$

can be derived by the link with the Hopf-Lax semigroup in $\mathfrak {C}$ given by [27, Theorem 8.11] and arguing as in [34, Thm. 7.36]. We prove it by a direct computation as follows: Set $\tau =t{-}s$, observe that $\inf {\mathscr {P}}_{\hspace{-2.0pt}\tau }\hspace{1.0pt}\xi _s=\inf \xi _t\leqq \frac{1}{2t}<\frac{1}{2\tau }$, and use $\xi _t = {\mathscr {P}}_{\hspace{-2.0pt}\tau }\hspace{1.0pt}\xi _s$ to obtain

4.47 $$\begin{aligned} \frac{1}{1{-}2\tau \xi _t(y)}= \inf _{z\in B_{{\pi /2}}(y)} \frac{1+2\tau \xi _s(z)}{\cos ^2_{{\pi /2}}\!{\left( |y{-}z|\right) }} \leqq \frac{1+2\tau \xi _s(x)}{\cos ^2_{{\pi /2}}\!{\left( |y{-}x|\right) }}\quad \text {if }|x{-}y|<\pi /2.\nonumber \\ \end{aligned}$$

With this estimate, we find

$$\begin{aligned} {\mathscr {R}}_{\hspace{-2.0pt}\tau }\hspace{1.0pt}\xi _t(x)&\overset{\text {def}}{=} \sup _{y\in B_{{\pi /2}}(x)}\frac{1}{2\tau }\Big (\frac{\cos ^2_{{\pi /2}}\!{\left( |x{-}y|\right) }}{1-2\tau \xi _s(y)}-1\Big ) \\ {}&\overset{{(4.47)}}{\leqq }\sup _{y\in B_{{\pi /2}}(x)} \frac{1}{2\tau }\Big (\frac{\cos ^2_{{\pi /2}}\!{\left( |x{-}y|\right) }}{\cos ^2_{{\pi /2}}\!{\left( |x{-}y|\right) }} \,(1{+}2\tau \xi _s(x))-1\Big ) \ = \ \xi _s(x). \end{aligned}$$

Using $\xi _t\geqq \xi _1\geqq {\bar{\xi }}_1$ we thus get (4.46). Passing to the limit as $t\uparrow 1$ in (4.46), we arrive at $\xi _s\geqq {\mathscr {R}}_{\hspace{-2.0pt}1-s}\hspace{1.0pt}{\bar{\xi }}_1={\bar{\xi }}_s$.

The closedness of $\Xi _t$ follows from the semi-continuities of $\xi _t$ and ${\bar{\xi }}_t$ and the estimate $\xi _t\geqq {\bar{\xi }}_t$. Indeed, assume $y_k\rightarrow y$ with $y_k\in \Xi _t$, then we have $y\in \Xi _t$ because of

$$\begin{aligned} \xi _t(y) \overset{\text {l.s.c.}}{\leqq }\liminf _{k\rightarrow \infty } \xi _t(y_k) = \liminf _{k\rightarrow \infty } {\bar{\xi }}_t(y_k) \leqq \limsup _{k\rightarrow \infty } {\bar{\xi }}_t(y_k) \overset{\text {u.s.c.}}{\leqq }{\bar{\xi }}_t(y) \leqq \xi _t(y). \end{aligned}$$

Assertion 2. Let us fix $x\in \Xi _t$, $\Lambda :=\Lambda _a(t)$ and ${\bar{\Lambda }}:=\Lambda _{{\bar{a}}}(1{-}t)$, and let *p* (resp. $p'$) be an element of the superdifferential of $x\mapsto \xi _t(x)-\frac{1}{2} \Lambda |x|^2$ (resp. of the subdifferential of $x\mapsto {\bar{\xi }}_t(x)+\frac{1}{2}{\bar{\Lambda }}|x|^2$). The superdifferential (subdifferential) is not empty, since the function is concave (convex) and finite everywhere. For every $x,y\in \mathbb {R}^d$ with $x\in \Xi _t$ we have

$$\begin{aligned} \langle p,y{-}x\rangle&\geqq \xi _t(y)-\xi _t(x)-\frac{1}{2} \Lambda |x{-}y|^2 \text { and } \langle p',y{-}x\rangle \\&\leqq {\bar{\xi }}_t(y)-{\bar{\xi }}_t(x)+\frac{1}{2} {\bar{\Lambda }}|x{-}y|^2. \end{aligned}$$

Subtracting the two inequalities and using $\xi _t(x)={\bar{\xi }}_t(x)$ and ${\bar{\xi }}_t(y)\leqq \xi _t(y)$ yields

$$\begin{aligned} \langle p'-p,y-x\rangle{} & {} \leqq {\bar{\xi }}_t(y)-\xi _t(y) +\frac{1}{2}(\Lambda {+}{\bar{\Lambda }}) |y{-}x|^2 \leqq \frac{1}{2}(\Lambda {+}{\bar{\Lambda }}) |y{-}x|^2\\{} & {} \quad \text {for every }y\in \mathbb {R}^d, \end{aligned}$$

so that $p=p'$ is uniquely determined and (4.40) holds.

Assertion 3. The fact that $\Xi ^\pm _t$ are independent of *t* and contained in $\Xi _t$ follows from (4.17) and (4.32). Moreover, (4.42) follows easily since $\xi _t$ takes its minimum at $\Xi ^-_t$ and its maximum at $\Xi ^+_t$.

Let us now fix $t\in (0,1)$, $x_0,x_1\in \Xi _t$, $p_i=\varvec{g}_t(x_i)+{\bar{\Lambda }} x_i$, and set ${\bar{\zeta }}(x):={\bar{\xi }}_t(x) + \frac{1}{2} {\bar{\Lambda }} |x|^2$, $\zeta (x):= \xi _t(x) +\frac{1}{2} {\bar{\Lambda }} |x|^2$. Notice that ${\bar{\zeta }}(x)$ is convex and $\zeta (x)$ is $C=\Lambda +{\bar{\Lambda }}$ semi-concave with ${\bar{\zeta }}(x)\leqq \zeta (x)$. We get

$$\begin{aligned} {\bar{\zeta }}(x_0)&\leqq {\bar{\zeta }}(x)-\langle p_0,x-x_0\rangle \leqq \zeta (x) -\langle p_0,x-x_0\rangle \\ {}&\leqq \zeta (x_1)+\langle p_1,x-x_1\rangle -\langle p_0,x-x_0\rangle + \frac{C}{2} |x{-}x_1|^2 \\ {}&= {\bar{\zeta }}(x_1) +\langle p_1-p_0,x-x_1\rangle -\langle p_0,x_1-x_0\rangle + \frac{C}{2} |x{-}x_1|^2. \end{aligned}$$

Minimizing with respect to *x* we find ${\bar{\zeta }}(x_0) \leqq {\bar{\zeta }}(x_1) -\langle p_0,x_1{-}x_0\rangle - \frac{1}{4C}|p_1{-}p_0|^2$. Inverting the role of $x_0$ and $x_1$ and summing up gives $\frac{1}{2C}|p_1{-}p_0|^2 \leqq \langle p_1{-}p_0,x_1{-}x_0\rangle$ and therefore

4.48 $$\begin{aligned} |p_1{-}p_0| \leqq 2C \,|x_1{-}x_0|. \end{aligned}$$

The boundedness of $\varvec{g}_t$ on $\Xi _t$ follows by the fact that $\xi _t$ is Lipschitz.

Assertion 4. Let us first consider the case $s>t$ with $\tau :=s{-}t$ and let $y\in \textsf{M}_{s\rightarrow t}(x)$. If $\xi _s(x)>-\frac{1}{2\tau }$ then *y* satisfies the identity (4.21). Since ${\bar{\xi }}_t(y)\leqq \xi _t(y)$, (4.35) and ${\bar{\xi }}_s(x)=\xi _s(x)$ yields ${\bar{\xi }}_t(y)=\xi _t(y)$ so that $y\in \Xi _t$ as well with  since ${\bar{\xi }}_t(y) \geqq -\frac{1}{2(1{-}t)}>-\frac{1}{2\tau }$.

Since the function $x'\mapsto (1{+}2\tau \xi _s(x'))\,(1{-}2\tau \xi _t(y)) -\cos ^2_{{\pi /2}}\!{\left( |x'{-}y|\right) }$ has a global minimizer at *x*, we arrive at the Euler–Lagrange equations

$$\begin{aligned} 2\tau (1{-}2\tau \xi _t(y))\, \varvec{g}_s(x) +2\cos _{{\pi /2}}\!{(|x{-}y|)}\,{\textbf {sin}}(x{-}y)=0 \end{aligned}$$

Since we can assume $|x{-}y|<\pi /2$ we obtain

4.49 $$\begin{aligned} x-y=-\textbf{arctan}\Big (\frac{\tau \varvec{g}_s(x)}{ 1 {+} 2\tau \xi _s(x)}\Big ), \end{aligned}$$

which characterizes *y* uniquely and establishes (4.43).

The case $t>s$ follows by the same arguments.

Assertion 5. This assertion is an immediate consequence of (4.43) and (4.42).

Assertion 6. The claims are simple consequences of the identity (4.15) and the definition of $q_{s\rightarrow t}$ of (4.44).

Assertion 7. The final assertion follows by (4.19) (and the corresponding (4.34)). $\quad \square$

#### Remark 4.4

(Strongly reduced pairs) It is worth noticing that if $\inf \xi _0>-\frac{1}{2}$ and $\sup {\bar{\xi }}_1<\frac{1}{2}$, then the sets $\Xi ^\pm _t$ in (4.41) are empty and many properties of $\xi _t$, $\varvec{T}_{s\rightarrow t}$ and $q_{s\rightarrow t}$ become considerably simpler. This situation is, e.g., the case of the solution induced by a strongly reduced pair with compact support, see Theorem 3.6.

We close this subsection by giving a small example for $\xi _t$ and ${\bar{\xi }}_t$ and their contact set $\Xi _t$ derived from an optimal pair $(\xi _0,{\bar{\xi }}_1)$ for the transport between two Dirac measures.

#### Example 4.5

(The contact set for two Dirac measures) For points $z_0,z_1\in \mathbb {R}^d$ and $r_0,r_1>0$ we consider the Dirac measures $\mu _j= r_j^2 \delta _{z_j}$. We have

$$\begin{aligned} \textsf{H}\!\!\textsf{K}^2(\mu _0,\mu _1)=r_0^2 + r_1^2 - 2r_0r_1 \cos _{{\pi /2}}\!{(\varrho )}\quad \text {with } \varrho = |z_1{-}z_0|, \end{aligned}$$

and all geodesic curves are known, see [26, Sec. 5.2]. For $\varrho < {\pi /2}$ we have a unique geodesic $\mu _t= r(t)^2 \delta _{z(t)}$ defined by transport, and for $\varrho >{\pi /2}$ the unique geodesic $\mu _t=(1{-}t)^2 r_0^2\delta _{z_0} + t^2r_1^2 \delta _{z_1}$ consists of growth (annihilation and decay) only. For $\varrho ={\pi /2}$ there is an infinite-dimensional convex set of geodesics, and we will see that this property is also reflected by a larger contact set.

Using the simple one-point supports of $\mu _j$ it is easy to calculate the optimal potentials and the transport plan ${\varvec{\eta }}$ in Theorem 2.14(ii). We obtain

$$\begin{aligned}{} & {} s_0:=\sigma _0(z_0)=\frac{r_1}{r_0}\,\cos _{{\pi /2}}\!{(\varrho )}, \quad s_1:=\sigma _1(z_1)=\frac{r_0}{r_1}\,\cos _{{\pi /2}}\!{(\varrho )}, \\{} & {} {\varvec{\eta }}= r_0r_1\cos _{{\pi /2}}\!{(\varrho )}\,\delta _{(z_0,z_1)}. \end{aligned}$$

Thus, we will distinguish the case $\cos _{{\pi /2}}\!{(\varrho )}>0$ and $\cos _{{\pi /2}}\!{(\varrho )}=0$.

$\underline{Case \varrho <{\pi /2}:}$ By (2.44) the optimal pair $(\xi _0,{\bar{\xi }}_1)$ reads as

$$\begin{aligned} \xi _0(x) = {\left\{ \begin{array}{ll} \frac{s_0{-}1}{2} &{}\text {for } x=z_0, \\ +\infty &{}\text {for } x\ne z_0; \end{array}\right. } \quad \text {and} \quad \xi _1(x) = {\left\{ \begin{array}{ll} \frac{1{-}s_1}{2} &{}\text {for } x=z_1, \\ -\infty &{}\text {for } x\ne z_1. \end{array}\right. } \end{aligned}$$

From these identities, we obtain the forward and backward solutions $\xi _t = {\mathscr {P}}_{\hspace{-2.0pt}t}\hspace{1.0pt}\xi _0$ and ${\bar{\xi }}_t = {\mathscr {R}}_{\hspace{-2.0pt}1{-}t}\hspace{1.0pt}\bar{\xi }_1$:

4.50 $$\begin{aligned} \xi _t(x) =\frac{1{-}t{+}ts_0- \cos ^2_{{\pi /2}}\!{\left( |x{-}z_0|\right) }}{2\,t\,(1{-}t{+}t s_0)} \quad \text {and} \quad {\bar{\xi }}_t(x) =\frac{\cos ^2_{{\pi /2}}\!{\left( |x{-}z_1|\right) } - t {-}(1{-}t) s_1}{2\,(1{-}t)\,(t{+}(1{-}t) s_1)}.\nonumber \\ \end{aligned}$$

The following optimality conditions can be checked by direct computation:

$$\begin{aligned} \text {(a) } \ {}&\xi _0\geqq {\bar{\xi }}_0 \text { and } \xi _1 \geqq {\bar{\xi }}_1 \text { \ on }\mathbb {R}^d \\ \text {(b) } \ {}&\xi _0 = {\bar{\xi }}_0 \ \ \mu _0\text {-a.e.}\quad \text {and} \quad \xi _1={\bar{\xi }}_1 \ \ \mu _1\text {-a.e.} \end{aligned}$$

As $\xi _0(x)=+\infty$ for $x\ne z_0$ and $\xi _1(x)=-\infty$ for $x\ne z_1$ statement (a) follows from (b). For (b) observe

$$\begin{aligned} {\bar{\xi }}_0(z_0){} & {} = \frac{\cos ^2\varrho -s_1}{2s_1} = \frac{ \cos ^2\varrho - (r_0/r_1) \cos \varrho }{2(r_0/r_1)\cos \varrho } = \frac{1}{2}\left( \frac{r_1}{r_0}\cos \varrho -1\right) =\frac{s_0{-}1}{2} \\{} & {} = \xi _0(z_0). \end{aligned}$$

Similarly, $\xi _1(z_1)={\bar{\xi }}_1(z_1)$ follows, which provides a first result on the contact sets $\Xi _t:= \big \{\, x\in \mathbb {R}^d \, \big | \, \xi _t(x)={\bar{\xi }}_t(x) \,\big \}$, namely $\Xi _0=\{z_0\}$ and $\Xi _1=\{z_1\}$.

**Fig. 3:**
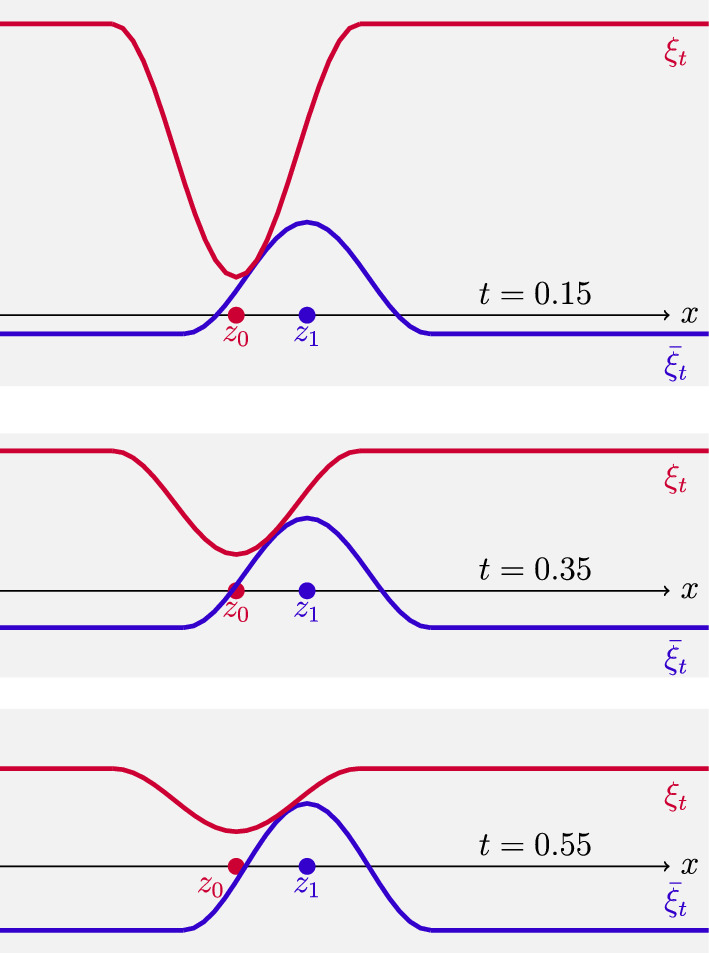
For the case $\varrho =|z_1{-}z_0|=0.9<{\pi /2}$ the functions $\xi _t(x)$ (red) and ${\bar{\xi }}_t(x)$ (blue) from (4.50) are displayed for the different times $t=0.15,\ 0.35$, and 0.55 (with parameter $r_1/r_0=2$). We always have $\xi _t(x) \geqq {\bar{\xi }}_t(x)$ with equality at the one-point contact set $\Xi _t=\{z(t)\}$, where $z(t) =\varvec{T}_{0\rightarrow t}(z_0)$ moves continuously from $z_0$ to $z_1$

The general theory in Theorem 4.3(i) guarantees $\xi _t\geqq {\bar{\xi }}_t$. A lengthy computation shows that $\Xi _t$ is a singleton also for $t\in (0,1)$, i.e. $\Xi _t=\{a(t)\}$ from $\mu _t=r(t)^2 \delta _{z(t)}$ and $\Xi ^\pm =\emptyset$. We refer to Fig. 3, where $x\mapsto (\xi _t(x),{\bar{\xi }}_t(t))$ is plotted.

$\underline{Case \varrho \geqq {\pi /2}:}$ Now we have $s_0=s_1=0$ and $\xi _t$ and ${\bar{\xi }}_t$ simplify accordingly:

4.51 $$\begin{aligned} \xi _t(x) = \frac{1-t - \cos ^2_{{\pi /2}}\!{\left( |x{-}z_0|\right) }}{ 2\,t\,(1{-}t)} \quad \text {and} \quad {\bar{\xi }}_t(x) = \frac{\cos ^2_{{\pi /2}}\!{\left( |x{-}z_1|\right) }-t }{ 2\,t\,(1{-}t)}. \end{aligned}$$

**Fig. 4:**
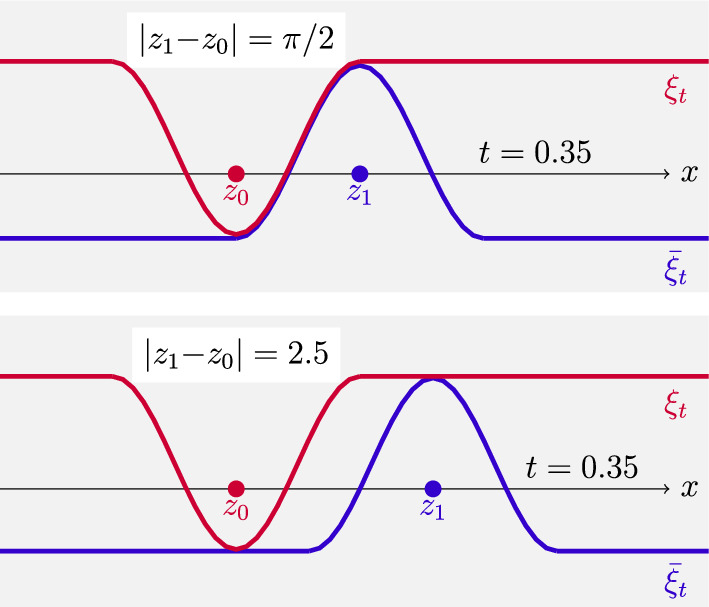
For $\varrho =|z_1{-}z_0|\geqq {\pi /2}$ the contact set $\Xi _t$ for the functions $\xi _t(x)$ (red) and ${\bar{\xi }}_t(x)$ (blue) from (4.51) is no longer a singleton. For $\varrho ={\pi /2}$ (upper figure) we obtain $\Xi _t=[z_0,z_1]$. For $\varrho >{\pi /2}$ (lower figure), we have $\Xi _t=\Xi ^+_t\cup \Xi ^-$ with $\Xi ^-_t=\{z_0\}$ and $\Xi ^+_t=\{z_1\}$

The contact sets are easily found depending on $\varrho ={\pi /2}$ or $\varrho >{\pi /2}$, namely

$$\begin{aligned} \varrho >{\pi /2}:\quad&\Xi _t= \Xi ^-\cup \Xi ^+_t \ \text { with } \Xi ^-=\{z_0\} \text { and } \Xi ^+_t=\{z_1\}, \\ \varrho ={\pi /2}: \quad&\Xi _t=[z_0,z_1] \text { and } \Xi ^-=\{z_0\} \text { and } \Xi ^+=\{z_1\}, \end{aligned}$$

where $[z_0,z_1]$ denotes the segment $\big \{\, (1{-}\theta )z_0{+}\theta z_1 \, \big | \, \theta \in [0,1] \,\big \}$, see Fig. 4.

The interesting fact that for $\varrho =|z_1{-}z_0|={\pi /2}$ the contact set $\Xi _t$ is constant and consists of a full segment reflects the observation in [26, Sec. 5.2] that $\mu _0$ and $\mu _1$ can be connected by geodesics satisfying $\textrm{sppt}(\mu _t)=[z_0,z_1]$ for all $t\in [0,1]$.

### Geodesic Flow and Characteristics

Finally, we study the differentiability of $\varvec{g}_s=\nabla \xi _s$ and $\varvec{T}_{t\rightarrow s}$ on $\Xi _s$. Let us denote by $\widetilde{\Xi }_t$ the subset of density points of the contact set $\Xi _t$, which is closed by (4.39):

4.52 $$\begin{aligned} x\in \widetilde{\Xi }_t\quad \Leftrightarrow \quad \lim _{\varrho \downarrow 0}\frac{{\mathcal {L}}^d(\Xi _t\cap B_\varrho (x))}{{\mathcal {L}}^d(B_\varrho (x))}=1. \end{aligned}$$

Notice that $\widetilde{\Xi }_t$ is just the set of Lebesgue points of the characteristic functions of $\Xi _t$, so that [1] ${\mathcal {L}}^d(\Xi _t\setminus \widetilde{\Xi }_t)=0$. By [9, Thm. 1], the family of sets $(\widetilde{\Xi }_t)_{t\in (0,1)}$ is invariant with respect to the action of the bi-Lipschitz maps $\varvec{T}_{s\rightarrow t}$, i.e., $\varvec{T}_{s\rightarrow t}(\widetilde{\Xi }_s)=\widetilde{\Xi }_t$ for every $s,t\in (0,1)$.

Given a locally Lipschitz function $F:\Xi _t\rightarrow \mathbb {R}^d$ and $x\in \widetilde{\Xi }_t$, we say that *F* is differentiable at *x* if there exists a matrix $\mathsf A=\textrm{D}F(x)\in \mathbb {R}^{d\times d}$ such that

4.53 $$\begin{aligned} |F(y)-F(x)-\mathsf A(y-x)|=o(|y-x|)\quad \text {as }y\rightarrow x,\ y\in \Xi _t. \end{aligned}$$

Since *x* belongs to the set $\widetilde{\Xi }_t$ of density points of $\Xi _t$, the matrix ${\mathsf A}$ is unique and every (locally) Lipschitz extension of *F* is differentiable at *x* with the same differential ${\mathsf A}$ (e.g. one can argue as in the proof of [1, Thm. 2.14]).

We call $\textrm{dom}_t (\textrm{D}F)$ the set of differentiability points $x\in \widetilde{\Xi }_t$ of *F*. If *F* is locally Lipschitz in $\Xi _t$, considering an arbitrary Lipschitz extension of *F* and applying Rademacher’s theorem, we know that ${\mathcal {L}}^d(\Xi _t\setminus \textrm{dom}_t(\textrm{D}F))=0$. We will use the simple chain-rule property that if $y=F(x)$ is a density point of $F(\Xi _t)$ and $H:F(\Xi _t)\rightarrow \mathbb {R}^k$ is differentiable at *y*, then

4.54 $$\begin{aligned} \textrm{D}(H\circ F)(x)=\textrm{D}H(F(x))\cdot \textrm{D}F(x). \end{aligned}$$

In the proof of the following lemma we will denote by $\partial \xi _s$ the Fréchet subdifferential of $\xi _s$, which coincides with $\nabla \xi _s$ whenever $\xi _s$ is differentiable, in particular in $x \in \Xi _s$.

#### Lemma 4.6

Let $s\in (0,1)$ and let $x\in \widetilde{\Xi }_s$ be a density point of $\Xi _s$ where $\varvec{g}_s=\nabla \xi _s$ is differentiable in the sense of (4.53) with $p=\varvec{g}_s(x)$ and ${\mathsf A}=\textrm{D}\nabla \xi _s(x)$. Then

4.55a $$\begin{aligned}{} & {} {\mathsf A}=\textrm{D}\nabla \xi _s(x)\quad \text {is symmetric,} \end{aligned}$$

4.55b $$\begin{aligned}{} & {} \sup _{z\in \partial \xi _s(y)} |z-p-{\mathsf A}(y{-}x)| = o\,(|y{-}x|) \quad \text {as }y\rightarrow x, \end{aligned}$$

4.55c $$\begin{aligned}{} & {} \xi _s(y)-\xi _s(x)-\langle p, y{-}x\rangle -\frac{1}{2} \langle {\mathsf A}(y{-}x), y{-}x\rangle = o\,(|y{-}x|^2) \quad \text {as }y\rightarrow x, \nonumber \\ \end{aligned}$$

Analogous results hold for ${\bar{\xi }}_s$. We will denote $\textrm{D}\nabla \xi _s$ by $\textrm{D}^2 \xi _s$.

Notice that the points *y* in the limits in (4.55b) and (4.55c) are not restricted to $\Xi _s$.

#### Proof

We adapt some ideas of [3, 5] to our setting, and we consider the case of ${\bar{\xi }}_s$ (to deal with a semi-convex function, instead of semi-concave). We will assume $x=0$ and will shortly write ${\bar{\xi }}$ and $\Xi$ for ${\bar{\xi }}_s$ and $\Xi _s$ omitting the explicit dependence on the parameter *s*. For $h>0$ we define the blowup set $\Xi ^{h}:=h^{-1}\Xi$. Up to an addition of a quadratic term, it is also not restrictive to assume that ${\bar{\xi }}$ is convex.

For $h>0$ we set $\omega _h(y):=\frac{1}{h^2}\big ({\bar{\xi }}(hy)-{\bar{\xi }}(x)-h\langle p,y\rangle \big )$ so that $\omega _h$ is a convex and nonnegative function. By (4.40) there exists a positive constant *C* such that

4.56 $$\begin{aligned} 0\leqq \omega _h(y)\leqq C|y|^2\quad \text {for every }y\in \Xi ^h. \end{aligned}$$

Since $x=0$ is a density point of $\Xi$, $\mathcal {L}^{d}(B_r(0){\setminus } \Xi ^h)\rightarrow 0$ as $h\downarrow 0$ so that every point of $z\in B_r(0)$ is a limit of a sequence in $z_h\in \Xi ^h\cap B_r(0)$. Therefore, for *h* sufficiently small we can find points $y_{h,i}\in \Xi ^h\cap B_{4d}(0)$, $i=1,\cdots , 2d$, such that $\overline{B_2(0)}\subset \textrm{conv}( \{\, y_{h,i} \, | \, i=1,\cdots , 2d \,\} )$. For this it is sufficient to approximate the (rescaled) elements of the canonical basis $\pm \mathsf e_i$, $i=1,\cdots , d$. If $y\in B_2(0)$ we then find coefficients $\alpha _{h,i}\geqq 0$, $\sum _i \alpha _{h,i}=1$ such that

$$\begin{aligned} \omega _h(y)\leqq \sum _i \alpha _{h,i}\omega _h(y_{h,i}) \leqq C \sum _i \alpha _{h,i}|y_{h,i}|^2\leqq 2dC \end{aligned}$$

so that $\omega _h$ is uniformly bounded in $B_2(0)$ and therefore is also uniformly Lipschitz in $\overline{B_1(0)}$. Every infinitesimal sequence $h_n\downarrow 0$ has a subsequence $m\mapsto h_{n(m)}$ such that $\omega _{h_{n(m)}}$ is uniformly convergent to a nonnegative, convex Lipschitz function $\omega :\overline{B_1(0)}\rightarrow \mathbb {R}$. We want to show that any limit point $\omega$ coincides with the quadratic function induced by the differential ${\mathsf A}$, namely $\omega (y)=\omega _{{\mathsf A}}(y)=\frac{1}{2}\langle {\mathsf A}y,y\rangle$

Let $\omega$ be the uniform limit of $\omega _h$ along a subsequence $h_n\downarrow 0$. If $y_n\in \Xi ^{h_n}\cap B_1(0)$ is converging to $y\in B_1(0)$ we know that any limit point of $p_n=\nabla \omega _{h_n}(y_n)$ belongs to $\partial \omega (y)$. On the other hand, $p_n=\frac{1}{h_n}(\nabla {\bar{\xi }}(h_ny_n)-p)={\mathsf A}y_n+o(1)$ thanks to the differentiability assumption, so that ${\mathsf A}y\in \partial \omega (y)$. Since we can approximate every point of $B_1(0)$ we conclude that ${\mathsf A}y\in \partial \omega (y)$ for every $y\in B_1(0)$. On the other hand, $\omega$ is Lipschitz, so that it is differentiable a.e. in $B_1(0)$ with $\nabla \omega (y)={\mathsf A}y$ and therefore the distributional differential of $\nabla \omega$ coincides with ${\mathsf A}$. We conclude that ${\mathsf A}$ is symmetric and $\omega (y)=\frac{1}{2}\langle {\mathsf A}y,y\rangle .$ The fact that $\omega _h$ uniformly converges to $\omega$ eventually yields (4.55b) and (4.55c). $\quad \square$

We now use the second-order differentiability of $\xi _s$ to derive differentiability of $\varvec{T}_{s\rightarrow t}$ by using the formula (4.43) with $\varvec{g}_s(x)=\nabla \xi _s(x)$. For $s \in (0,1)$ we define

4.57 $$\begin{aligned} \mathfrak {D}_s = \textrm{dom}_s(\textrm{D}\nabla \xi _s)) \cap \widetilde{\Xi }_s = \textrm{dom}_s(\textrm{D}^2\xi _s) \cap \widetilde{\Xi }_s. \end{aligned}$$

As we already observed, since $\varvec{g}_s$ is Lipschitz on $\Xi _s$, $\mathcal {L}^{d}(\Xi _s{\setminus } \mathfrak {D}_s)=0$ for every $s\in (0,1)$.

For $t\in (0,1)$ and $\tau =t{-}s$ we also have $1{+}2\tau \xi _s\geqq (1{-}t)/(1{-}s)>0$ so that

$$\begin{aligned} x \mapsto \frac{\tau }{1{+}2\tau \xi _s(x)}\,\nabla \xi _s(x) = \nabla \phi _{s,t}(x) \text { with } \phi _{s,t}(x)= \frac{1}{2} \log \big (1{+}2\tau \xi _s(x) \big ) \end{aligned}$$

is again Lipschitz on $\Xi _s$. Thus, Lemma 4.6 can be applied and $\phi _{s,t}$ is differentiable in the sense of (4.53) on $\mathfrak {D}_s$. Finally, we exploit the explicit representation of $\varvec{T}_{s\rightarrow t}$ via (4.43), namely for all $x\in \Xi _s$ we have

4.58 $$\begin{aligned} \varvec{T}_{s\rightarrow t} (x) = x + \textbf{arctan}\Big ( \frac{\tau \nabla \xi _s(x)}{1{+}2\tau \xi _s(x)}\Big ) = x + \textbf{arctan}\big ( \nabla \phi _{s,t}(x)\big ). \end{aligned}$$

Now the chain rule (4.54) guarantees the differentiability of $\varvec{T}_{s\rightarrow t}$ on the set $\mathfrak {D}_s$.

#### Lemma 4.7

(Differentiability of $\varvec{T}$) For all $s,t\in (0,1)$ the mapping $\varvec{T}_{s\rightarrow t}$ is differentiable on $\mathfrak {D}_s$, and we have

4.59a $$\begin{aligned}&\begin{aligned}&\textrm{D}\varvec{T}_{s\rightarrow t}(x)= \mathbb {T}\big (t{-}s,\xi _s(x),\nabla \xi _s(x),\textrm{D}^2\xi _s(x) \big ) \quad \text {with} \\&\mathbb {T}(\tau ,\xi ,\varvec{g},{\mathsf A}):=\mathbb {I}+\big (\textrm{D}^2 \textrm{L}_1(z)\big )^{-1}_{\big |{z=\textbf{arctan}(\tfrac{\tau \varvec{g}}{1{+}2\tau \xi })}} \Big ( \frac{\tau \, {\mathsf A}}{1{+}2\tau \xi } - \frac{2\tau ^2\, \varvec{g}{\otimes }\varvec{g}}{(1{+}2\tau \xi )^2} \Big ). \end{aligned} \end{aligned}$$

4.59b $$\begin{aligned}&\varvec{T}_{s\rightarrow t}(\mathfrak {D}_s)=\mathfrak {D}_t \hbox { and } \textrm{D}\varvec{T}_{t\rightarrow s}(\varvec{T}_{s\rightarrow t}(x))\textrm{D}\varvec{T}_{s\rightarrow t}(x)=\mathbb {I}\text { for }x\in \mathfrak {D}_s. \end{aligned}$$

For every $t_0,t_1\in (0,1)$, $t_2\in [0,1]$ we also have

4.59c $$\begin{aligned}&\textrm{D}\varvec{T}_{t_1\rightarrow t_2}(\varvec{T}_{t_0\rightarrow t_1}(x))\textrm{D}\varvec{T}_{t_0\rightarrow t_1}(x)= \textrm{D}\varvec{T}_{t_0\rightarrow t_2}(x)\quad \text {for }x\in \mathfrak {D}_{t_0}. \end{aligned}$$

#### Proof

Recall $\tau = t{-}s$, then the explicit formula (4.59a) follows from differentiating $\nabla \textrm{L}_1\big (x{-}\varvec{T}_{s\rightarrow t} (x) \big )= - \frac{\tau }{1+2\tau \xi _s} \nabla \xi _s$. Since $\varvec{T}_{s\rightarrow t}^{-1}=\varvec{T}_{t\rightarrow s}$ there exists a constant *L* such that

4.60 $$\begin{aligned} L^{-1}|x{-}x'|\leqq |\varvec{T}_{s\rightarrow t}(x)-\varvec{T}_{s\rightarrow t}(x')|\leqq L|x{-}x'| \quad \text {for every }x,x'\in \Xi _s. \qquad \end{aligned}$$

If $x\in \mathfrak {D}_s$ and $A=\textrm{D}\varvec{T}_{s\rightarrow t}(x)$, choosing $\varepsilon >0$ we can find $\varrho >0$ such that

4.61 $$\begin{aligned} |\varvec{T}_{s\rightarrow t}(x')-\varvec{T}_{s\rightarrow t}(x)-A(x'{-}x)|\leqq \varepsilon |x'{-}x|\quad \text {for every }x'\in \Xi _t\cap B_\varrho (x),\nonumber \\ \end{aligned}$$

so that choosing $\varepsilon <\frac{1}{2\,L}$ and $x'=x+v$ we get

$$\begin{aligned} |Av|\geqq |\varvec{T}_{s\rightarrow t}(x{+}v)-\varvec{T}_{s\rightarrow t}(x)|- \varepsilon |v| \geqq \frac{1}{2L}|v|\quad \text {for every }v\in B_\varrho (0)\cap (\Xi _t-x) \end{aligned}$$

Using the fact that 0 is a density point of $\Xi _t-x$ we conclude that *A* is invertible with $|A^{-1}|\leqq 2L$. For every $y'\in \Xi _t$ with $L|y'{-}y|<\varrho$ and $x'=\varvec{T}_{t\rightarrow s}(y')$, we get $|x'{-}x|<\varrho$ and (4.61) yields

$$\begin{aligned} |\varvec{T}_{t\rightarrow s}(y')-\varvec{T}_{t\rightarrow s}(y)-A^{-1}(y'{-}y)|&=\big |A^{-1} \big (A(x'{-}x)-\varvec{T}_{s\rightarrow t}(x')+\varvec{T}_{s\rightarrow t}(x)\big )\big | \\ {}&\leqq {2L} \varepsilon |x'{-}x|\leqq 2L^2\varepsilon |y'{-}y| \end{aligned}$$

showing that $y\in \mathfrak {D}_t$ and $A^{-1}=\textrm{D}\varvec{T}_{t\rightarrow s}(y)$. Hence, (4.59b) is established.

Equation (4.59c) then follows by the concatenation property (4.45). $\quad \square$

The explicit formula (4.59a) shows that $\textrm{D}\varvec{T}_{s\rightarrow t}$ is the product of the positive matrix $\textrm{D}^2\textrm{L}_1(z)^{-1}$ and a symmetric matrix, hence it is always real diagonalizable. The following result shows that the determinant and hence all eigenvalues stay positive for $s,t\in (0,1)$. In fact, we now derive differential equations with respect to $t\in (0,1)$ for the transport-growth pairs $(\varvec{T}_{s\rightarrow t}(x), q_{s\rightarrow t}(x)) \in \mathbb {R}^d\times (0,+\infty )$ as well as for $\textrm{D}\varvec{T}_{s\rightarrow t}(x) \in \mathbb {R}^{d\times d}$ and $\det \textrm{D}\varvec{T}_{s\rightarrow t}(x)$. Recall that $t\mapsto (\varvec{T}_{s\rightarrow t}(x), q_{s\rightarrow t}(x))$ is analytic for $t\in (0,1)$ by Theorem 4.35 and 6.

The following relations will be crucial to derive the curvature estimate needed for our main result on geodesic $\textsf{H}\!\!\textsf{K}$-convexity.

#### Theorem 4.8

(The characteristic system on the contact set $\Xi _s$) We fix $s\in (0,1)$, $x\in \Xi _s$, and $y\in \mathfrak {D}_s$ (cf. (4.57)) and define the maps

$$\begin{aligned} \varvec{T}(t):=\varvec{T}_{s\rightarrow t}(x), \quad q(t):=q_{s\rightarrow t}(x), \quad {\mathsf B}(t):= \textrm{D}\varvec{T}_{s\rightarrow t}(y), \ \ \text { and }\ \delta (t):= \det {\mathsf B}(t). \end{aligned}$$

Then, we have the initial conditions $\varvec{T}(s)=x$, $q(s)=1$, ${\mathsf B}(s)=\mathbb {I}$, and $\delta (s)=1$, and for $t\in (0,1)$ the following differential equations are satisfied:

4.62a $$\begin{aligned}&{\dot{\varvec{T}}}(t)=\nabla \xi _t(\varvec{T}(t)){} & {} \text {and } \ \ddot{\varvec{T}}(t)= -4\xi _t(\varvec{T}(t))\,\nabla \xi _t(\varvec{T}(t)), \end{aligned}$$

4.62b $$\begin{aligned}&\dot{q}(t)=2\xi _t(\varvec{T}(t))\,q(t){} & {} \text {and }\ \ddot{q}(t)= |\nabla \xi _t(\varvec{T}(t))|^2\, q(t), \end{aligned}$$

4.62c $$\begin{aligned}&{\dot{{\mathsf B}}}(t) =\textrm{D}^2\xi _t(\varvec{T}(t)) \, {\mathsf B}(t){} & {} \text {and }\ \ddot{{\mathsf B}}(t) = -4\Big (\nabla \xi _t{\otimes }\nabla \xi _t+ \xi _t\textrm{D}^2\xi _t\Big ) {\circ } \varvec{T}(t)\cdot {\mathsf B}(t), \end{aligned}$$

4.62d $$\begin{aligned}&{\dot{\delta }}(t)= \Delta \xi _t(\varvec{T}(t)) \, \delta (t),{} & {} \frac{\ddot{\delta }(t)}{\delta (t)}=\Big ((\Delta \xi _t)^2 {-} |\textrm{D}^2\xi _t|^2 {-} 4|\nabla \xi _t|^2 {-} 4\xi _t\Delta \xi _t\Big ) {\circ } \varvec{T}(t). \end{aligned}$$

there $$\Delta \xi _t(z)= \textrm{tr}\big (\textrm{D}^2 \xi _t(z)\big )$$ and $$|\textrm{D}^2\xi _t(z)|^2 = \sum _{i,j} \big (\partial _{x_i}\partial _{x_j}\xi _t(z)\big )^2$$.

#### Proof

We use (4.45) and the Taylor expansion

$$\begin{aligned} \textbf{arctan}\Big (\frac{ h \varvec{g}}{ 1{+}2h \xi } \Big )\ = h \varvec{g}- 2h^2 \xi \varvec{g}+ O(h^3)\quad \text {as }h\rightarrow 0. \end{aligned}$$

Setting $y=\varvec{T}(t)=\varvec{T}_{s\rightarrow t}(x)$ and using the fact that $y\in \Xi _t$, (4.58) yields

$$\begin{aligned} \varvec{T}_{t\rightarrow t+h}(y)=y+h \nabla \xi _t(y)-2h^2 \xi _t(y)\nabla \xi _t(y)+ O(|h|^3)\ \text { as } h \rightarrow 0. \end{aligned}$$

With the composition rule (4.45) we have $\varvec{T}_{s\rightarrow t+h} (x)=\varvec{T}_{t\rightarrow t+h}(y)$ and compute

$$\begin{aligned} {\dot{\varvec{T}}}(t)&= \lim _{h\rightarrow 0}\frac{\varvec{T}_{s\rightarrow {t+h}}(x)-\varvec{T}_{s\rightarrow t}(x)}{h} = \lim _{h\rightarrow 0}\frac{\varvec{T}_{t\rightarrow {t+h}}(y)-y}{h} = \nabla \xi _t(y). \end{aligned}$$

This identity yields the first equation in (4.62a). For the second relation in (4.62a) we use

$$\begin{aligned} \ddot{\varvec{T}}(t)&= \lim _{h\rightarrow 0}\frac{\varvec{T}_{s\rightarrow {t+h}}(x)-2\varvec{T}_{s\rightarrow t}(x)+\varvec{T}_{s\rightarrow t-h}(x)}{h^2}\\&= \lim _{h\rightarrow 0}\frac{\varvec{T}_{t\rightarrow {t+h}}(y)-2y+\varvec{T}_{t\rightarrow {t-h}}(y)}{h^2} =-4\xi _t(y)\nabla \xi _t(y). \end{aligned}$$

The relations (4.62b) for $q(t)=q_{s\rightarrow t}$ follow similarly, using the scalar product rule for $q_{s\rightarrow t}$ in (4.45) and by taking the square root of (4.44), namely,

$$\begin{aligned} q_{t\rightarrow t+h}(y)=1+2h \xi _t(y)+ \frac{h^2}{2} |\nabla \xi _t(y)|^2 + o(h^2)\quad \text {as }h\rightarrow 0. \end{aligned}$$

To show that ${\mathsf B}(t)$ satisfies (4.62c), we exploit the matrix product rule (4.59c) and expand $\textrm{D}\varvec{T}_{t\rightarrow t+h}(y)$ in (4.59a) to obtain

4.63 $$\begin{aligned} \textrm{D}\varvec{T}_{t\rightarrow t+h}(y) = \mathbb {I}+ h \textrm{D}^2 \xi _t - 2h^2 \Big ( \nabla \xi _t{\otimes }\nabla \xi _t + \xi _t \textrm{D}^2 \xi _t\Big ) + o(h^2) \quad \text {as }h\rightarrow 0. \nonumber \\ \end{aligned}$$

For this note that $y-\varvec{T}_{t\rightarrow t+h}(y)=O(|h|)$ so that $\textrm{D}^2\textrm{L}_1\big (y{-}\varvec{T}_{t\rightarrow t+h}(y)\big )= \mathbb {I}+ O(|h|^2)$ as $\textrm{L}_1$ is even. Thus, (4.62c) follows as in the previous two cases.

For the determinant $\delta (t)$ we again have a scalar product rule, and it suffices to expand $\det (\textrm{D}\varvec{T}_{t\rightarrow t+h}(x))$ at $h=0$. For this we can use the classical expansion $\det (\mathbb {I}{+}h\mathbb {A})= 1 + h\mathop {\textrm{tr}}\mathbb {A}+\frac{1}{2} h^2 \big ( (\mathop {\textrm{tr}}\mathbb {A})^2 - \mathop {\textrm{tr}}(\mathbb {A}^2)\big ) + O(h^3)$, and obtain

4.64 $$\begin{aligned} \det \textrm{D}\varvec{T}_{t\rightarrow t+h}= 1+h \Delta \xi _t+ \frac{1}{2} h^2 \Big ( (\Delta \xi _t)^2 - |\textrm{D}^2\xi _t|^2 -4|\nabla \xi _t|^2-4\xi \Delta \xi _t\Big ) +o(h^2).\nonumber \\ \end{aligned}$$

As before this shows (4.62d), and the theorem is proved. $\quad \square$

In this section, we have studied the forward solutions $t\mapsto \xi _t$ for $t\in (0,1)$ and its contact sets $\Xi _t$ with a corresponding backward solution ${\bar{\xi }}_t$. We obtained differentiability properties in these sets or in the slightly smaller sets $D_t$ and derived transport relations for important quantities such as $q_{s\rightarrow t}$ and $\delta _s(t)=\det \textrm{D}\varvec{T}_{s\rightarrow t}(x)$. In the following section, we still have to show that the contact sets $\Xi _t$ are sufficiently big, if we define $\xi _t={\mathscr {P}}_{\hspace{-2.0pt}t}\hspace{1.0pt}\xi _0$ and ${\bar{\xi }}_t = {\mathscr {R}}_{\hspace{-2.0pt}1-t}\hspace{1.0pt}\xi _1$ for an optimal pair $(\xi _0,\xi _1)$. This will be done in Theorem 5.1.

## Geodesic Curves

In this section, we improve the characterization of Hellinger–Kantorovich geodesic curves as discussed already in [27, Sec. 8.6]. More precisely, we consider constant-speed geodesics $\mu :[0,1]\rightarrow \mathcal {M}({\mathbb {R}^{d}})$ that satisfy

$$\begin{aligned} \forall \, s,t \in [0,1]: \quad \textsf{H}\!\!\textsf{K}(\mu (s),\mu (t)) = |s{-}t| \, \textsf{H}\!\!\textsf{K}(\mu _0,\mu _1). \end{aligned}$$

We first show the optimality of potentials $\xi _t$ and ${\bar{\xi }}_t$ obtained from the forward or backward Hamilton–Jacobi equation in Theorem 5.1. With this, we are able to show in Theorem 5.2 that for subparts $(s,t) \subset [0,1]$ with $\tau =t{-}s<1$ the corresponding LET problem has a unique solution in Monge form, which implies that $(\mathcal {M}(\mathbb {R}^d),\textsf{H}\!\!\textsf{K})$ has the strong non-branching property. Finally, in Theorem 5.4 and Corollary 5.5 we provide restrictions and splittings of geodesic curves needed for the main theorem in Section 7.

### Geodesics and Hamilton–Jacobi Equation

The next result clarifies the connection with the forward and backward Hopf–Lax flows $\xi _t$ and ${\bar{\xi }}_t$ studied in Theorem 4.3 and the importance of the contact set $\Xi _t$ defined in (4.39) (see also [27, Thm. 8.20] and [34, Chap. 7] for a similar result in the framework of Optimal Transport and displacement interpolation). We emphasize that despite the non-uniqueness of the geodesics $(\mu _t)_{t\in [0,1]}$ (see [26, Sec. 5.2]) in the following result, $\xi _t$ and ${\bar{\xi }}_t$ only depend on $\mu _0$ and $\mu _1$ and the optimal potentials $\varphi _0$ and $\varphi _1$.

The result brings together the results of Sects. 3 and 4 by starting with an optimal pair $(\varphi _0,\varphi _1)$ from Section 3 and considering the corresponding solutions $\xi _t$ and ${\bar{\xi }}_t$ of the forward and backward Hamilton–Jacobi equation starting with $\xi _0 = \check{\textsf{G}}_1(\varphi _0)$ and ${\bar{\xi }}_1 = \textsf{G}_1(\varphi _1)$, respectively. First, we observe that “intermediate” pairs $(\xi _s,\xi _t)$ or $({\bar{\xi }}_s,{\bar{\xi }}_t)$ are optimal for connecting the intermediate points $\mu _s$ and $\mu _t$ on an arbitrary geodesic connecting $\mu _0$ and $\mu _1$. Second, we observe that certain results obtained in Section 4 for $s,t\in (0,1)$ also hold in the limit points $s,t\in \{0,1\}$. Finally, we show that the contact set $\Xi _t$ is large enough in the sense that it contains $\textrm{supp}(\mu _t)$ (see Example 4.5 for some instructive case with $\varrho ={\pi /2}$).

#### Theorem 5.1

For $\mu _0,\mu _1\in \mathcal {M}({\mathbb {R}^{d}})$ consider a tight optimal pair $(\varphi _0,\varphi _1)$ of (lower,upper) semi-continuous potentials as in Theorem 3.3. With $\xi _0:=\check{\textsf{G}}_1(\varphi _0)= \frac{1}{2}(\textrm{e}^{2\varphi _0}-1)$ and ${\bar{\xi }}_1:= \textsf{G}_1(\varphi _1)= \frac{1}{2}(1-\textrm{e}^{-2\varphi _1})$ we define $\xi _t={\mathscr {P}}_{\hspace{-2.0pt}t}\hspace{1.0pt}\xi _0$ and ${\bar{\xi }}_t = {\mathscr {R}}_{\hspace{-2.0pt}1-t}\hspace{1.0pt}\bar{\xi }_1$ as in (4.30) and the contact sets $\Xi _t=\{\xi _t={\bar{\xi }}_t\}$ as in (4.39). Finally, consider an arbitrary geodesic $(\mu _t)_{t\in [0,1]}$ connecting $\mu _0$ to $\mu _1$. Then, the following holds:

1. For all $s,t \in [0,1]$ with $s<t$ both pairs $(\xi _s,\xi _t)$ and $({\bar{\xi }}_s,{\bar{\xi }}_t)$ are optimal for (2.40) and (2.41) for connecting $\mu _s$ to $\mu _t$, viz.
  5.1 $$\begin{aligned} \frac{1}{2(t{-}s)}\textsf{H}\!\!\textsf{K}^2(\mu _s,\mu _t)= \int \xi _t\;\!\textrm{d}\mu _t-\int \xi _s\;\!\textrm{d}\mu _s= \int {\bar{\xi }}_t\;\!\textrm{d}\mu _t-\int {\bar{\xi }}_s\;\!\textrm{d}\mu _s\qquad \end{aligned}$$
2. $$S_t={\text {supp}}(\mu _t)\subset \Xi _t$$ for every $$t\in [0,1]$$.

#### Proof

Assertion (1). It is sufficient to consider the forward flow ${\mathscr {P}}_{\hspace{-2.0pt}t}\hspace{1.0pt}$. Fixing

$$t\in (0,1)$$ we have

5.2 $$\begin{aligned} \frac{1}{2t}\textsf{H}\!\!\textsf{K}^2(\mu _0,\mu _t){} & {} \geqq \int \xi _t\;\!\textrm{d}\mu _t-\int \xi _0\;\!\textrm{d}\mu _0 \text { and } \frac{1}{2(1{-}t)}\textsf{H}\!\!\textsf{K}^2(\mu _t,\mu _1)\nonumber \\{} & {} \geqq \int \xi _1\;\!\textrm{d}\mu _1-\int \xi _t\;\!\textrm{d}\mu _t. \end{aligned}$$

On the other hand, the geodesic property and the optimality of $(\xi _0,\xi _1)$ yield

$$\begin{aligned} \int \xi _1\;\!\textrm{d}\mu _1-\int \xi _0\;\!\textrm{d}\mu _0=\frac{1}{2}\textsf{H}\!\!\textsf{K}^2(\mu _0,\mu _1)= \frac{1}{2t}\textsf{H}\!\!\textsf{K}^2(\mu _0,\mu _t)+ \frac{1}{2(1{-}t)}\textsf{H}\!\!\textsf{K}^2(\mu _t,\mu _1) \end{aligned}$$

showing that the inequalities in (5.2) are in fact equalities, in particular (5.1) with $s=0$. For $s>0$ we still get (5.1) since $\frac{1}{2(t-s)}\textsf{H}\!\!\textsf{K}^2(\mu _s,\mu _t) = \frac{1}{2t}\textsf{H}\!\!\textsf{K}^2(\mu _0,\mu _t)-\frac{1}{2\,s}\textsf{H}\!\!\textsf{K}^2(\mu _0,\mu _s)$ if $0<s<t\leqq 1$.

Assertion (2). Equation (5.1) for $s=0$ yields $\int (\xi _t-{\bar{\xi }}_t)\;\!\textrm{d}\mu _t=0$ for all $t\in (0,1)$, so that $\xi _t \leqq {\bar{\xi }}_t$ and the continuity of $\xi _t,{\bar{\xi }}_t$ yield $\xi _t={\bar{\xi }}_t$ on $S_t = {\text {supp}}\mu _t$. The cases $t=0$ and 1 follow by the relations between $\xi _i$ and $\varphi _i$ and the fact that , . $\quad \square$

Note that the inclusion $S_t={\text {supp}}(\mu _t)\subset \Xi _t$ is in general a strict inclusion. This can be seen for the case $|z_1{-} z_0| = {\pi /2}$ in Example 4.5, where $\Xi _t =[z_0,z_1]$, however, there exists a pure Hellinger geodesic with $\textrm{supp}(\mu _t)= \{z_0,z_1\}$ for $t\in (0,1)$.

We can now exploit all the regularity features of the maps $\varvec{T}_{s\rightarrow t}$ and $q_{s\rightarrow t}$ on the contact set $\Xi _t$ (cf. Theorem 4.3). A first important consequence is that, given an $\textsf{H}\!\!\textsf{K}$ geodesic $(\mu _t)_{t\in [0,1]}$ and $s\in (0,1)$, the $\textsf{H}\!\!\textsf{K}$ problem between $\mu _s$ and $\mu _t$ for any $t \in [0,1]$ has only one solution, which can be expressed in Monge form (see [2, Lem. 7.2.1] for the corresponding properties for the $\textrm{L}^2$-Wasserstein distance in $\mathbb {R}^d$).

#### Theorem 5.2

(Regularizing effect along geodesics) Under the assumptions of Theorem 5.1, if $s\in (0,1)$ and $t\in [0,1]$, then the transport-growth pair $(\varvec{T}_{s\rightarrow t}, q_{s\rightarrow t})$ of Theorem 4.3 is the unique solution of the Monge formulation (2.21) of the Entropy-Transport problem between $\mu _s$ and $\mu _t$. In particular, the optimal Entropy-Transport problem between $\mu _s$ and $\mu _0$ or between $\mu _s$ and $\mu _1$ has a unique solution, and this solution is in Monge form.

#### Proof

Let us consider the case $0<s<t\leqq 1$, $\tau =t-s<t$. By Theorem 5.1, the pair $({\bar{\xi }}_s,{\bar{\xi }}_t)$ is optimal for $(\mu _s,\mu _t)$ and ${\text {supp}}(\mu _s)\subset \Xi _s$. Using the transformations

5.3 $$\begin{aligned} \varphi _0:=\frac{1}{2\tau }\log (1{+}2\tau {\bar{\xi }}_s)\quad \text {and} \quad \varphi _\tau :=-\frac{1}{2\tau }\log (1{-}2\tau {\bar{\xi }}_t), \end{aligned}$$

we see that $(\varphi _{0},\varphi _{\tau })$ is a pair of potentials satisfying the assumptions of Theorem 3.3(2). Since $1-2\tau \xi _t\geqq 1-\tau /t>0$ we deduce that $\varphi _\tau$ is bounded from above, so that $\mu _t''=0$ thanks to (3.26) (where the measures $\mu _t'$ and $\mu _t''$ are defined as in (2.13)).

Moreover, we know that $\mu _s'$ is concentrated on $\{\varphi _0>-\infty \}$; since it is also concentrated on $\Xi _s$ we deduce that $\mu _s'$ is concentrated on $D_0' = \textrm{dom}(\nabla \varphi _0)$, so that we can apply Corollary 3.5, recalling the expression of $\varvec{T},q$ given by (3.34). $\quad \square$

The above theorem allows us to deduce the fact that $(\mathcal {M}(\mathbb {R}^d),\textsf{H}\!\!\textsf{K})$ has a strong non-branching property. It is shown in [26, Sec. 5.2] that the set of geodesics connecting two Dirac measures $\delta _{y_0}$ and $\delta _{y_1}$ is very large if $|y_1{-}y_0|=\pi /2$: it is convex but does not lie in a finite-dimensional space. The following result shows that all these geodesics are mutually disjoint except for the two endpoints $\mu _0$ and $\mu _1$:

#### Corollary 5.3

(Strong non-branching) If for some $s\in (0,1)$ we have $\textsf{H}\!\!\textsf{K}(\mu _0,\mu _s)=s\textsf{H}\!\!\textsf{K}(\mu _0,\mu _1)$ and $\textsf{H}\!\!\textsf{K}(\mu _s,\mu _1)=(1{-}s)\textsf{H}\!\!\textsf{K}(\mu _0,\mu _1)$, then there exists a unique geodesic curve $t\mapsto \mu (t)$ such that $\mu (0)=\mu _0$, $\mu (s)=\mu _s$, and $\mu (1)=\mu _1$.

The next result shows that from a given geodesic we may construct new geodesics by multiplying the measures $\mu _t$ by a suitably transported function. This will be useful in the proof of the main Theorem 7.2.

#### Theorem 5.4

(Restriction of geodesics) Let $(\mu _t)_{t\in [0,1]}$ be an $\textsf{H}\!\!\textsf{K}$ geodesic. For a given $s\in (0,1)$ let $\nu _s\in \mathcal {M}(\mathbb {R}^d)$ with ${\text {supp}}(\nu _s)\subset {\text {supp}}(\mu _s)$. Then the curve $[0,1]\ni t\mapsto \nu _t:=(\varvec{T}_{s\rightarrow t},q_{s\rightarrow t})_\star \nu _s$ is also an $\textsf{H}\!\!\textsf{K}$ geodesic. If in addition $\nu _s=\varrho _s\mu _s$ for some Borel function $\varrho _s:{\text {supp}}(\mu _s)\rightarrow [0,+\infty ]$, then $\nu _t' = \varrho _t\mu _t$ with $\varrho _t(y)= \varrho _s(\varvec{T}_{t\rightarrow s} (y))$ for every $t\in (0,1)$.

#### Proof

We keep the same notation of Theorem 5.1, let $0<t_1<s<t_2<1$, and set $\tau _1:=s-t_1$, $\tau _2:=t_2-s$, and $\tau =\tau _1{+}\tau _2$. We clearly have

$$\begin{aligned} \frac{1}{2\tau }\textsf{H}\!\!\textsf{K}^2(\nu _{t_2},\nu _{t_1})&\geqq \int \xi _{t_2}\;\!\textrm{d}\nu _{t_2}- \int \xi _{t_1}\;\!\textrm{d}\nu _{t_1}\\&= \Big (\int \xi _{t_2}\;\!\textrm{d}\nu _{t_2}-\int \xi _s\;\!\textrm{d}\nu _s\Big )+ \Big (\int \xi _s\;\!\textrm{d}\nu _s-\int \xi _{t_1}\;\!\textrm{d}\nu _{t_1}\Big ) \end{aligned}$$

The conclusion then follows, if we show that $\int \xi _{t_2}\;\!\textrm{d}\nu _{t_2}-\int \xi _s\;\!\textrm{d}\nu _s\geqq \frac{1}{2\tau _2}\textsf{H}\!\!\textsf{K}^2(\nu _{t_2},\nu _s)$ and $\int \xi _{s}\;\!\textrm{d}\nu _{s}-\int \xi _{t_1}\;\!\textrm{d}\nu _{t_1}\geqq \frac{1}{2\tau _2}\textsf{H}\!\!\textsf{K}^2(\nu _s,\nu _{t_1})$. We check the first inequality, the second follows similarly.

Define $q_2:=q_{s\rightarrow t_2}$ and $\varvec{T}_2:=\varvec{T}_{s\rightarrow t_2}$. Using the fact that (i) $\nu _{t_2}=(\varvec{T}_{2},q_{2})_\star \nu _s$ and (ii) identity (4.44) we obtain

$$\begin{aligned} \int (1 {-} 2\tau _2 \xi _{t_2})\;\!\textrm{d}\nu _{t_2} \overset{\text {(i)}}{=} \int \Big (1{-}2\tau _2\xi _{t_2}\big (\varvec{T}_{s\rightarrow t_2}(x)\big )\Big )q^2_{s\rightarrow t_2} (x) \;\!\textrm{d}\nu _{s}(x) \overset{\text {(ii)}}{=} \int (1{+}2\tau _2\xi _s) \;\!\textrm{d}\nu _s . \end{aligned}$$

Combining (4.43) and (4.44), we arrive at

5.4 $$\begin{aligned} \int \big (1-2\tau \xi _{t_2}\big ) \;\!\textrm{d}\nu _{t_2}= \int \big (1+2\tau _2\xi _s\big ) \;\!\textrm{d}\nu _s= \int q_2\cos (|x{-}\varvec{T}_2(x)|)\;\!\textrm{d}\nu _s. \end{aligned}$$

With this, we find

$$\begin{aligned} \textsf{H}\!\!\textsf{K}^2(\nu _{t_2},\nu _s)&\overset{{(2.17)}}{\leqq }\int \big ( q_2^2+1-2q_2\cos (|x{-}\varvec{T}_2(x)|\big ) \;\!\textrm{d}\nu _s \\&\overset{{(5.4)}}{=} \nu _{t_2}(\mathbb {R}^d) + \nu _{s} (\mathbb {R}^d) - \int (1{-}2\tau \xi _{t_2})\;\!\textrm{d}\nu _{t_2}- \int (1{+}2\tau _2\xi _s)\;\!\textrm{d}\nu _s \\&=2\tau \Big (\int \xi _{t_2}\;\!\textrm{d}\nu _{t_2}- \int \xi _s\;\!\textrm{d}\nu _s\Big ). \end{aligned}$$

Hence, we have shown $\frac{1}{2\tau }\textsf{H}\!\!\textsf{K}^2(\nu _{t_2},\nu _{t_1}) = \int \xi _{t_2}\;\!\textrm{d}\nu _{t_2}- \int \xi _s\;\!\textrm{d}\nu _s$, which implies that $(\nu _t)_{t\in (0,1)}$ is a geodesic as well.

We can then pass to the limits $t_1\downarrow 0$ and $t_2\uparrow 1$ as follows. Notice that the curve $t\mapsto \nu _{t}$, $t\in (0,1)$, is converging in $(\mathcal {M}(\mathbb {R}^d),\textsf{H}\!\!\textsf{K})$ to a limit $\nu _0$ and $\nu _1$ for $t\downarrow 0$ and $t\uparrow 1$, since $(\nu _t)$ is a geodesic. Moreover, for every $\zeta \in \textrm{C}_b(\mathbb {R}^d)$ we can pass to the limit $t\uparrow 1$ in

5.5 $$\begin{aligned} \int \zeta \;\!\textrm{d}\nu _t=\int \zeta (\varvec{T}_{s\rightarrow t}(x))q^2_{s\rightarrow t}(x)\;\!\textrm{d}\nu _s(x), \end{aligned}$$

since $\lim _{t\uparrow 1}\varvec{T}_{s\rightarrow t}(x)= \varvec{T}_{s\rightarrow 1}(x)$ and $\lim _{t\uparrow 1}q_{s\rightarrow t}(x)= q_{s\rightarrow 1}(x)$ and *q* is uniformly bounded. A similar argument holds for the case $t\downarrow 0$.

In order to check the identity concerning the density $\varrho _t'$ of $\nu _t$, we use (5.5) and find

$$\begin{aligned} \int \zeta \;\!\textrm{d}\nu _t&= \int \zeta (\varvec{T}_{s\rightarrow t}(x))q^2_{s\rightarrow t}(x)\;\!\textrm{d}\nu _s =\int \zeta (\varvec{T}_{s\rightarrow t}(x))q^2_{s\rightarrow t}(x)\varrho _s(x)\;\!\textrm{d}\mu _s \\&= \int \zeta (\varvec{T}_{s\rightarrow t}(x))q^2_{s\rightarrow t}(x)\varrho _t(\varvec{T}_{s\rightarrow t}(x))\;\!\textrm{d}\mu _s(x)= \int \zeta (y)\varrho _t(y)\;\!\textrm{d}\mu _t(y). \end{aligned}$$

The case $t \in [0,s]$ is analogous. $\quad \square$

The next result provides the fundamental formula for the representation of densities along geodesics. Generalizing the celebrated formulas for the Kantorovich–Wasserstein geodesics, the densities are again obtained by transport along geodesics, but now with non-constant speed and an additional growth factor $a_s(t,x) =q^2_{s\rightarrow t}(x)$ to account for the annihilation and creation of mass. Recall that $\mathfrak {D}_s= \textrm{dom} (\textrm{D}^2\xi _s) \subset \Xi _s$ has full Lebesgue measure in $\Xi _s$, i.e. $\mathcal {L}^{d}(\Xi _s{\setminus } \mathfrak {D}_s)=0$.

#### Corollary 5.5

(Representation of densities along geodesics) For $\mu _0,\mu _1\in \mathcal {M}(\mathbb {R}^d)$ consider a geodesic $(\mu _t)_{t\in [0,1]}$ connecting $\mu _0$ to $\mu _1$. Assume that at least one of the following properties holds:

- there exists $s\in (0,1)$ such that $\mu _s=c_s\mathcal {L}^{d}\ll \mathcal {L}^{d}$;
- $$\mu _0=c_0\mathcal {L}^{d}\ll \mathcal {L}^{d}$$ and $$\mu _{1}''\ll \mathcal {L}^{d}$$.

Then, we have

1. $\mu _t\ll {\mathcal {L}}^d$ for every $t\in (0,1)$, viz. $\mu _t = c(t,\cdot ) \mathcal {L}^{d}$.
2. For every $s\in (0,1)$ the density $c(t,\cdot )$ can be expressed via the formula
  5.6a $$\begin{aligned} c(t,y)\big |_{y= \varvec{T}_{s\rightarrow t}(x)} = c(s,x)\frac{\alpha _s(t,x)}{\delta _s(t,x)} \quad \text {for every }x\in \mathfrak {D}_s,\quad t\in (0,1), \end{aligned}$$
  with $\mathfrak {D}_s = \textrm{dom}_s(\textrm{D}\nabla \xi _s))= \textrm{dom}_s(\textrm{D}^2\xi _s)$ (cf. (4.57)) and
  5.6b $$\begin{aligned} \alpha _s(t,x):= \big (1+2(t{-}s)\xi _s(x)\big )^2+(t{-}s)^2|\nabla \xi _s(x)|^2, \quad \delta _s(t,x):=\det \textrm{D}\varvec{T}_{s\rightarrow t}(x).\nonumber \\ \end{aligned}$$
  Moreover, we have $\textrm{D}^2\xi _s(x)=0$ and $\delta _s(t,x)=1$ for $\mathcal {L}^{d}$-a.e. $x\in \Xi ^0_s\supset \Xi ^\pm$; in particular
  5.7 $$\begin{aligned} c(t,x)=\frac{t^2}{s^2}c(s,x)\quad \text {for }x\in \Xi ^+\quad \text {and} \quad c(t,x)=\frac{(1{-}t)^2}{(1{-}s)^2}c(s,x)\quad \text {for }x\in \Xi ^-.\nonumber \\ \end{aligned}$$
3. If $\mu _0\ll \mathcal {L}^{d}$ (resp. $\mu _1\ll \mathcal {L}^{d}$) (5.6) and (5.7) hold up to $t=0$ (resp. up to $t=1$).
4. If $\mu _1''=0$ the representations in (5.6) also hold for $s=0$ by restricting *x* in $D_0''=\textrm{dom}(\textrm{D}^2\varphi _0)$, and we have the formula
  5.8 $$\begin{aligned} \textrm{D}\varvec{T}_{0\rightarrow t}(x) = \mathbb {T}\big (t,\xi _0(x),\nabla \xi _0(x),\textrm{D}^2\xi _0(x)\big ) \quad \text {for every }x\in D_0'', \end{aligned}$$
  where $\mathbb {T}$ is defined in (4.59a).

#### Proof

Assertion (1). In the case (a) holds for $s\in (0,1)$, there exists a bi-Lipschitz map $\varvec{T}_{s,t}:\Xi _s\rightarrow \Xi _t$ and bounded growth factors $q_{s,t}:\Xi _s\rightarrow [a,b]$ with $0<a<b<\infty$ such that $\mu _t=(\varvec{T}_{s\rightarrow t},q_{s\rightarrow t})_\star \mu _s$. In particular, for every Borel set *A* we have

5.9 $$\begin{aligned} \mu _t(A)\leqq b^2\mu _s(\varvec{T}_{s\rightarrow t}^{-1}(A)) =b^2\mu _s(\varvec{T}_{t\rightarrow s}(A)). \end{aligned}$$

If $\mathcal {L}^{d}(A)=0$ then $\mathcal {L}^{d}(\varvec{T}_{t\rightarrow s}(A))=0$ because $\varvec{T}_{t\rightarrow s}$ is Lipschitz. Hence, using $\mu _s \ll \mathcal {L}^{d}$ we find $\mu _s(\varvec{T}_{t\rightarrow s}(A)=0$, such that (5.9) gives $\mu _t(A)=0$. With this we conclude $\mu _s\ll \mathcal {L}^{d}$.

In the case of assumption (b), we argue as before but with $\mu _0=c_0\mathcal {L}^{d}$ for $s=0$. Using the fact that $q_{t\rightarrow 0}$ is locally bounded from below and that $\varvec{T}_{t\rightarrow 0}$ is locally Lipschitz on $A_t:=\Xi _t\setminus \Xi ^+$, we deduce that  On the other hand we have $\mu ''_1\ll \mathcal {L}^{d}$ and the restriction of $\varvec{T}_{t\rightarrow 1}$ to $\Xi ^+$ coincides with the identity and $q_{t\rightarrow 1}$ is bounded from below thanks to (4.44). Thus, we obtain $\mu _t\ll \mathcal {L}^{d}$.

Assertion (2). The representation (5.6a) follows by Theorem 5.2 and Corollary 3.5.

Relation (5.7) can be deduced directly by Theorem 5.2. In order to prove that $\textrm{D}^2\xi _s=0$ $\mu _s$-a.e. in $\Xi ^\pm$ it is sufficient to consider density points of $\Xi ^\pm$, since $\mu _s\ll \mathcal {L}^{d}$, and to compute the differential of $\nabla \xi _s$ on $\Xi ^\pm$, where it is constant.

Assertions (3) and (4). Both assertions follow from Corollary 3.5. $\quad \square$

As a last application, we will also discuss the propagation of the singular part with respect to $\mathcal {L}^{d}$, which will be needed in the proof of the main result in Theorem 7.2.

#### Corollary 5.6

(Propagation of the singular part) Let $\mu _0,\mu _1\in \mathcal {M}(\mathbb {R}^d)$ and let $(\mu _t)_{t\in [0,1]}$ be a geodesic connecting $\mu _0$ to $\mu _1$ and let $\mu _s=\mu _s^\textrm{a}+\mu _s^\perp$ be the decomposition of $\mu _s$ with respect to the Lebesgue measure $\mathcal {L}^{d}$ at some point $s\in (0,1)$. For every $t\in [0,1]$ we set

5.10 $$\begin{aligned} \widetilde{\mu }_t:=(\varvec{T}_{s\rightarrow t},q_{s\rightarrow t})_\star \mu _s^a \quad \text {and} \quad \widehat{\mu }_t:=(\varvec{T}_{s\rightarrow t},q_{s\rightarrow t})_\star \mu _s^\perp . \end{aligned}$$

Then, the curves $(\widetilde{\mu }_t)_{t\in (0,1)}$ and $(\widehat{\mu }_t)_{t\in (0,1)}$ are $\textsf{H}\!\!\textsf{K}$ geodesics, we have $\widehat{\mu }_t \perp \mathcal {L}^{d}$ for $t\in [0,1]$ and $\mu _t= \widetilde{\mu }_t + \widehat{\mu }_t$ provides the Lebesgue decomposition for $t\in (0,1)$, viz. $\mu ^\textrm{a}_t=\widetilde{\mu }_t$ and $\mu _t^\perp = \widehat{\mu }_t$.

#### Proof

Let us decompose $\Xi _s$ in the disjoint union of two Borel sets *A*, *B* such that  and  with $\mathcal {L}^{d}(B)=0$. By Theorem 5.4 we clearly have $\mu _t=\widetilde{\mu }_t + \widehat{\mu }_t$. On the one hand, $\widetilde{\mu }_t \ll \mathcal {L}^{d}$ by Corollary 5.5 for all $t\in (0,1)$. On the other hand, for all $t\in [0,1]$ the measure $\widehat{\mu }_t$ is concentrated on the set $\varvec{T}_{s\rightarrow t}(B)$ which is $\mathcal {L}^{d}$-negligible, since $\varvec{T}_{s\rightarrow t}$ is Lipschitz. If follows that $\widehat{\mu }_t \perp \mathcal {L}^{d}$, so that $\widehat{\mu }_t = \mu _t^\textrm{a}$ and $\widehat{\mu }_t=\mu _t^\perp$ for all $t\in (0,1)$.

The fact that $(\mu _t^a)$ and $(\mu _t^\perp )$ are geodesics follows by Theorem 5.4 as well. $\quad \square$

### Convexity of the Lebesgue Density Along $\textsf{H}\!\!\textsf{K}$-Geodesics

In this subsection, we consider geodesics $(\mu _t)_{t\in [0,1]}$ such that $\mu _s\ll \mathcal {L}^{d}$ for some, and thus for all, $s\in (0,1)$. We fix *s* and introduce the functions $\alpha _s,\delta _s$ as in (5.6b) and the functions

5.11 $$\begin{aligned} \left. \begin{aligned} \gamma _s(t,x):={}&\alpha _s^{1/2}(t,x)=q_{s\rightarrow t}(x),\\ \rho _s(t,x):={}&\alpha _s^{1/2}(t,x)\delta _s^{1/d}(t,x)= q_{s\rightarrow t}(x)\big ( \! \det \textrm{D}\varvec{T}_{s\rightarrow t}(x)\big )^{1/d} \end{aligned} \right\} \quad \text { for } x\in D_s.\nonumber \\ \end{aligned}$$

We now exploit the explicit differential relations for $\gamma _s(t,x)=q_{s\rightarrow t}(x)$ and $\delta _s(t,x)= \det \textrm{D}\varvec{T}_{s\rightarrow t}(x)$ provided in Theorem 4.8 and derive lower estimates for $\ddot{\gamma }_s$ and $\ddot{\rho }_s$. It remains unclear whether the given choice for $\gamma _s$ and $\rho _s$ is the only possible, however it turns out that for these variables the following curvature estimates are relatively simple and hence the final convexity calculus goes through. For comparison, we mention that in the Kantorovich–Wasserstein case we have $\gamma _{\textsf{K}}{\textsf{W}}(t)\equiv 1$ and $\rho _{\textsf{K}}{\textsf{W}}(t)= \big (\delta _{\textsf{K}}{\textsf{W}}(t)\big )^{1/d}$ with $\delta _{\textsf{K}}{\textsf{W}}(t)=\det ((1{-}t)\mathbb {I}{+}t\textrm{D}\varvec{T}_{\textsf{K}}{\textsf{W}}(x))$, such that $\ddot{\rho }_{\textsf{K}}{\textsf{W}}(t) \leqq 0$ since $\textrm{D}\varvec{T}_{\textsf{K}}{\textsf{W}}(x)$ is diagonalizable with nonnegative real eigenvalues, see [2, Eqn. (9.3.12)].

#### Proposition 5.7

(Curvature estimates for $(\rho ,\gamma )$) Let $(\rho _s,\gamma _s):(0,1)\times \mathfrak {D}_s\rightarrow [0,\infty [^2$ be defined as above along a geodesic. Then, we have for all, $t\in (0,1),$ the relations

5.12 $$\begin{aligned} \frac{\ddot{\gamma }_s(t,x)}{\gamma _s(t,x)} \geqq 0 \quad \text {and} \quad \left\{ \begin{array}{cl} \displaystyle \frac{\ddot{\rho }_s(t)}{\rho _s(t)}\leqq \Big (1{-}\frac{4}{d}\Big )\frac{\ddot{\gamma }_s(t)}{\gamma _s(t)} &{}\quad \text {for }d\geqq 2, \\ \displaystyle \frac{\ddot{\rho }_s(t)}{\rho _s(t)} = \Big (1{-}\frac{4}{d}\Big )\frac{\ddot{\gamma }_s(t)}{\gamma _s(t)} &{}\quad \text {for }d=1. \end{array}\right. \end{aligned}$$

#### Proof

As $s \in (0,1)$ and $x\in \mathfrak {D}_s$ are fixed, we will simply write $\rho (t)$ instead of $\rho _s(t,x),$ and do similarly for the other variables. Using the specific definition of $\rho$ we obtain

$$\begin{aligned} \frac{\ddot{\rho }}{\rho }= \frac{\ddot{\gamma }}{\gamma }+ \frac{2}{d} \,\frac{{\dot{\gamma }}}{\gamma }\, \frac{{\dot{\delta }}}{\delta }+ \frac{1}{d}\, \frac{\ddot{\delta }}{\delta }+ \frac{1}{d}\left( \frac{1}{d}-1\right) \left( \frac{{\dot{\delta }}}{\delta }\right) ^2. \end{aligned}$$

We can now use the formulas provided in (4.62a)–(4.62d) giving ${\dot{\gamma }} = 2\xi _t \gamma$ and $\ddot{\gamma } = |\nabla \xi _t|^2 \gamma$, where $\xi _t$ and its derivatives are evaluated at $y=\varvec{T}_{s\rightarrow t} (x)$. Inserting this and (4.62d) for ${\dot{\delta }}$ and $\ddot{\delta }$ into the above relation for $\ddot{\delta }/\delta$ we observe significant cancellations and obtain

5.13 $$\begin{aligned} \frac{\ddot{\gamma }}{\gamma }= |\nabla \xi _t|^2 \quad \text {and}\quad \frac{\ddot{\rho }}{\rho } = \frac{1}{d^2}\big ( (\Delta \xi _t)^2 {-}d|\textrm{D}^2\xi _t|^2\big )+\big (1- \frac{4}{d}\big )|\nabla \xi _t|^2. \end{aligned}$$

For $d=1$ we have $\textrm{D}^2\xi = \Delta \xi$, while for $d\geqq 2$ all matrices $A\in \mathbb {R}^{d\times d}$ satisfy $d|A|^2 = d \sum _{i,j=1}^d A_{ij}^2 \geqq (\mathop {\textrm{tr}}A)^2 = \big (\sum _{1}^d A_{ii}\big )^2$. Thus, the curvature estimates (5.12) follow. $\quad \square$

The above curvature estimates will be crucial in Section 7 for deriving our main result on geodesic convexity. We remark that for $d\geqq 2$ they are even slightly better that the “sufficient curvature estimates” given in (7.3) because of $1-4/d\leqq 1-4/d^2$ (with equality only for $d=1$).

We finally derive a useful result concerning the convexity of the density $t \mapsto c(t,x)$ along geodesics. This provides a direct proof of the fact, which was used in [13] that the $\textrm{L}^\infty$-norm along geodesics is bounded by the $\textrm{L}^\infty$-norm of the two endpoints. Indeed, we show more, namely that the function $t \mapsto c(t,\varvec{T}_t(x))$ is either trivially constant or it is strictly convex.

#### Theorem 5.8

(Convexity of densities along geodesics)

*(1)* Under the assumption of Corollary 5.5, for every $s\in (0,1)$ and $x\in \mathfrak {D}_s\cup \Xi ^\pm$ the function $c_s(t)=c(t,\varvec{T}_{s\rightarrow t}(x))$ given by (5.6a) or (5.7), respectively, is convex and positive in (0, 1); moreover, with a possible $\mathcal {L}^{d}$-negligible exception, it is either constant or strictly convex.

*(2)* If moreover $\mu _0\ll \mathcal {L}^{d}$ (resp. $\mu _1\ll \mathcal {L}^{d}$) then for $\mu _s$-a.e. *x* their limit as $t\downarrow 0$ (resp. as $t\uparrow 1$) coincides with $c_0\circ \varvec{T}_{s\rightarrow 0}$ (resp. $c_1\circ \varvec{T}_{s\rightarrow 1}$).

#### Proof

Assertion (1). Since $x\in {\mathbb {R}^{d}}$ and $s\in (0,1)$ play no role, we drop them for notational simplicity. We simply calculate the second derivative of the function $t\mapsto c(t)=\gamma (t)^{d+2}c_s/\rho (t)^{ d }$. If $c_s=c(s,x)=0$ then $c(t,\varvec{T}_{s\rightarrow t}(x))=0$ and the result is obviously true. Hence, we may assume $c_s>0$ and obtain after an explicit calculation

5.14 $$\begin{aligned} \ddot{c}= c\,\Big ((d{+}2) \frac{\ddot{\gamma }}{\gamma }- d \frac{\ddot{\rho }}{\rho }+ (d{+}1)(d{+}2) \big (\frac{{\dot{\gamma }}}{\gamma }\big )^2 - 2d(d{+}2) \frac{{\dot{\gamma }}}{\gamma }\,\frac{{\dot{\rho }}}{\rho }+ d(d{+}1) \big (\frac{{\dot{\rho }}}{\rho }\big )^2 \Big ).\nonumber \\ \end{aligned}$$

The quadratic form involving the first derivatives is positive definite, and for the terms involving the second derivatives we can use the curvature estimates in (5.12) to obtain

$$\begin{aligned} \ddot{c} \geqq c \,\Big ( \big ( (d{+}2) \frac{\ddot{\gamma }}{\gamma }- d\big (1 - \frac{4}{d}\big ) \frac{\ddot{\gamma }}{\gamma }+ 0 \Big )= 6 \, c \, \frac{\ddot{\gamma }}{\gamma }. \end{aligned}$$

Notice that $t\mapsto \gamma (t)$ is the square root of the non-negative (and strictly positive in (0, 1)) quadratic polynomial $\alpha (\cdot ,x)$ given by (5.6b), so that $\gamma ''\geqq 0$ and we conclude that $\ddot{c}(t)\geqq 0$ as well due to $c(t)>0$.

Moreover, if $x\not \in \Xi ^0_s$ then $|\nabla \xi _s(x)|>0$, have $\ddot{\gamma }(t)>0$, and we deduce that $\ddot{c}(t)>0$ obtaining the strict convexity of *c*.

If $x\in \Xi ^0_s$ where $\nabla \xi _s(x)=0$, we can use the representation (5.7) for *c* up to a $\mathcal {L}^{d}$-negligible set.

Assertion (2). If $\mu _0=c_0\mathcal {L}^{d}\ll \mathcal {L}^{d}$, then $\delta _s(0,x)>0$ for $\mu _s$-a.a. $x\in \mathfrak {D}_s$ thanks to the last statement of Corollary 3.5 (which is a direct consequence of Theorem 3.35) and both $\delta _s(0,x)$ and $\alpha _s(0,x)$ coincides with their limit as $t\downarrow 0$. A further application of Corollary 5.5(3) yields the result. The case $t=1$ is completely analogous. $\quad \square$

The above result easily provides the following statement on convexity of $\textrm{L}^\infty$ norms along $\textsf{H}\!\!\textsf{K}$-geodesics. This can be generalized to a corresponding result for the Kantorovich–Wasserstein geodesics (which might been known, but the authors were not able to identify a reference, see the Remark 5.10 below):

#### Corollary 5.9

(Convexity of the $L^\infty$ norm along geodesics) Let $\mu _0,\mu _1\in \mathcal {M}(\mathbb {R}^d)$ be absolutely continuous with respect to $\mathcal {L}^{d}$ with densities $c_i\in L^\infty (\mathbb {R}^d)$ and let $(\mu _t)_{t\in [0,1]}$ be a $\textsf{H}\!\!\textsf{K}$ geodesic connecting $\mu _0$ to $\mu _1$. Then $\mu _t=c_t\mathcal {L}^{d}$ and $\Vert c_t\Vert _{L^\infty }\leqq (1{-}t)\Vert c_0\Vert _{L^\infty }+ t\Vert c_1\Vert _{L^\infty }$.

#### Proof

The result for $(\mathcal {M}(\mathbb {R}^d),\textsf{H}\!\!\textsf{K})$ follows directly from Theorem 5.8. $\quad \square$

#### Remark 5.10

Let $(\mu ^\textrm{W}_t)_{t\in [0,1]}$ be the Kantorovich–Wasserstein geodesic connection between two probability measures $\mu _0,\mu _1 \in {\mathcal {P}}_2(\mathbb {R}^d)$ with $\mu _i=c_i \mathcal {L}^{d}$ and $c_0,c_1 \in L^\infty (\mathbb {R}^d)$. Similar to the previous result, $\mu ^\textrm{W}_t=c^\textrm{W}_t\mathcal {L}^{d}$ is absolutely continuous w.r.t. $\mathcal {L}^{d}$ and $\Vert c^\textrm{W}_t\Vert _{L^\infty }\leqq (1{-}t)\Vert c^\textrm{W}_0\Vert _{L^\infty }+ t\Vert c^\textrm{W}_1\Vert _{L^\infty }$.

In fact, for $({\mathcal {P}}_2(\mathbb {R}^d), \textsf{W}_2)$ we replace (5.6) by the simpler formula for the Kantorovich–Wasserstein transport

$$\begin{aligned} c^\textrm{W}(t,\varvec{T}_{s\rightarrow t}(x) ) = \frac{c^\textrm{W}_s(x)}{\delta _s(t,x)} \quad \text {with } \delta _s(x) = \det \varvec{T}^\textrm{W}_{ s\rightarrow t}(x); \end{aligned}$$

see [2, Prop. 9.3.9]. Using $\mu _0=c_0 \mathcal {L}^{d}$ we can choose $s=0$ and have $\varvec{T}^\textrm{W}_{0\rightarrow t}(x) = x + t(\nabla \varphi (x){-}x)$ for a convex Kantorovich potential. Since for every symmetric positive semidefinite matrix *D* the function $t \mapsto 1/\det \big ( (1{-}t)I + tD\big )$ is convex, the desired result follows with the same arguments as for Theorem 5.8.

## Preliminary Discussion of the Convexity Conditions

In this section, we discuss the equivalence of two formulations of the convexity conditions and give a few examples. The proof of sufficiency and necessity of these conditions is then given in the following Section 7.

For most parts of this section, we assume that $E:{[0,\infty [}\rightarrow \mathbb {R}\cup \{\infty \}$ is lower semi-continuous and convex, satisfies $E(0)=0$, and is twice continuously differentiable on the interior of its domain $D(E):= \{\, c\geqq 0 \, | \, E(c)<\infty \,\}$. The following result gives a characterization of the conditions (1.25) on $N_E:(\rho ,\gamma )\mapsto (\rho /\gamma )^d E(\gamma ^{d+2}/\rho ^d)$ in terms of the derivatives of *E*, namely $\varepsilon _j(c)=c^j E^{(j)}(c)$ for $j=0$, 1, and 2, which appear in

6.1 $$\begin{aligned} \mathbb {B}(c):= \left( \begin{array}{cc} \varepsilon _2(c)- \frac{d-1}{d}\big (\varepsilon _1(c){-}\varepsilon _0(c)\big ) &{} \varepsilon _2(c) - \frac{1}{2}\big (\varepsilon _1(c){-}\varepsilon _0(c) \big ) \\ \varepsilon _2(c)- \frac{1}{2}\big (\varepsilon _1(c){-}\varepsilon _0(c) \big ) &{} \varepsilon _2(c) + \frac{1}{2} \varepsilon _1(c) \end{array} \right) . \end{aligned}$$

This characterization will then be used to derive a nontrivial monotonicity result in Proposition 6.2, which is a crucial building block of the main geodesic convexity result.

Note that the variables $\rho$ and $\gamma$ are related to the variable *c* via $c= c_0\gamma ^{d+2}/\rho ^d$.

### Proposition 6.1

(Equivalent conditions on *E*) Let $N_E$ and $\mathbb {B}$ be defined in terms of *E* as in (1.25a) and (6.1), respectively. Then the following conditions are equivalent:

(A) $$N_E$$ satisfies (1.25);
(B) in the interior of the domain *D*(*E*) we have $\mathbb {B}(c)\geqq 0$ and $(d{-}1)\big (\varepsilon _1(c){-}\varepsilon _0(c)\big )\geqq 0$.

### Proof

We first observe that the desired monotonicity of $\rho \mapsto N_E(\rho ,\gamma )$ for $d\geqq 2$ is indeed equivalent to the condition $\varepsilon _1(c)\geqq \varepsilon _0(c)$. This follows easily from the relation

$$\begin{aligned} \partial _\rho N_E(\rho ,\gamma ){} & {} = \frac{d\rho ^{d-1}}{\gamma ^d} \;\!E\big (\frac{\gamma ^{d+2}}{\rho ^d} \big ) +\frac{\rho ^d}{\gamma ^d}\;\!E'\big (\frac{\gamma ^{d+2}}{\rho ^d} \big ) \big ({-}d\frac{\gamma ^{d+2}}{\rho ^{d+1}}\big )\\{} & {} = \frac{d\rho ^{d-1}}{\gamma ^d}\;\!\big ( \varepsilon _0(c)- \varepsilon _1(c)\big ). \end{aligned}$$

It remains to establish the equivalence between the convexity of $N_E$ and the positive semi-definiteness of $\mathbb {B}$. For this we note that $N_E$ is given as a linear function of *E*, hence the Hessian $\textrm{D}^2N_E$ will be a given as a linear combination of *E*, $E'$, and $E''$. Indeed, an explicit calculation yields

$$\begin{aligned} \textrm{D}^2 N_E(\rho ,\gamma ) = \frac{\rho ^d}{\gamma ^d}\left( \begin{array}{cc}d/\rho &{}-d/\gamma \\ 0 &{}-2/\gamma \end{array} \right) ^\top \mathbb {B}\big (\frac{\gamma ^{d+2}}{\rho ^d}\big ) \left( \begin{array}{cc}d/\rho &{}-d/\gamma \\ 0 &{}-2/\gamma \end{array} \right) . \end{aligned}$$

With this, we see that $\textrm{D}^2 N_E$ is positive semidefinite if and only if $\mathbb {B}$ is. Hence, the assertion is proved. $\quad \square$

From the semi-definiteness of the matrix $\mathbb {B}(c)$, we obtain as necessary conditions the non-negativity of the two diagonal elements which provide the McCann condition $\mathbb {B}_{11}=\varepsilon _2- \frac{d{-}1}{d}(\varepsilon _1- \varepsilon _0) \geqq 0$ and the convexity conditions with respect to the Hellinger–Kakutani distance $\mathbb {B}_{22}=\varepsilon _2+\frac{1}{2}\varepsilon _1\geqq 0$. Moreover, testing $\mathbb {B}$ with $(1,-1)^\top$ reveals the additional condition

6.2 $$\begin{aligned} \left( {\begin{array}{c}1\\ -1\end{array}}\right) \cdot \mathbb {B}(c)\left( {\begin{array}{c}1\\ -1\end{array}}\right) \geqq 0 \quad \Longleftrightarrow \quad (d{+}2)\varepsilon _1(c)-2\varepsilon _0(c)\geqq 0. \end{aligned}$$

### Proposition 6.2

(New necessary monotonicity) Let *E* be such that the conditions in Proposition 6.1 hold and let $N_E$ be defined via (1.25a). Then, the following three equivalent conditions hold:

(A) The function ${]0,\infty [}\ni c\mapsto c^{-2/(d+2)} E(c)$ is non-decreasing.
(B) For all $\rho ,\gamma >0$ we have the inequality $\big ( 1- \frac{4}{d^2}\big ) \rho \partial _\rho N_E(\rho ,\gamma ) + \gamma \partial _\gamma N_E(\rho ,\gamma )\geqq 0$.
(C) For all $\rho ,\gamma >0$ the mapping ${]0,\infty [}\ni s \mapsto N_E(s^{1-4/d^2} \rho ,s \gamma )$ is non-decreasing.

### Proof

Expressing $\partial _\rho N_E$ and $\partial _\gamma N_E$ via $\varepsilon _0$ and $\varepsilon _1$ and using $\delta =(\rho /\gamma )^d$ we obtain

$$\begin{aligned} \rho \partial _\rho N_E(\rho ,\gamma )= -d\delta (\varepsilon _1{-}\varepsilon _0) \quad \text {and} \quad \gamma \partial _\gamma N_E(\rho ,\gamma )= \delta \big ((d{+}2)\varepsilon _1-d \varepsilon _0\big ). \end{aligned}$$

Thus, we conclude $(1{-} \frac{4}{d^2}) \rho \partial _\rho N_E(\rho ,\gamma ) + \gamma \partial _\gamma N_E(\rho ,\gamma )\,=\, \frac{2\delta }{d}\,\big ((d{+}2)\varepsilon _1 - 2\varepsilon _0\big )$, which is positive because of (6.2). Thus, (B) is established and the monotonicity of $s \mapsto N_E(s^{1-4/d^2} \rho ,s \gamma )$ in (C) follows simply by differentiation.

Statement (A) follows by applying (C) for $\rho =\gamma =1$ and choosing $s = c^{2(d{+}2)/d}$. $\quad \square$

The crucial monotonicity stated at the end of the above proposition means

6.3 $$\begin{aligned} 0\leqq c_1 < c_2\quad \Longrightarrow \quad E(c_1) \,\leqq \big (\frac{c_1}{c_2}\big )^{2/(d+2)} E(c_2). \end{aligned}$$

It implies that if *E* attains a negative value it cannot be differentiable at $c=0$: If $E(c_1)<0$ then $E(c) \leqq (c/c_1)^{2/(d+2)} E(c_1)<0$, which leads to $E'(c)\searrow -\infty$ for $c \searrow 0$.

In the next examples we investigate which functions *E* satisfy the above conditions. The following two results will be used in Corollary 7.3 to obtain geodesic convexity for functionals of the form $E(c)=\int _\Omega a c^r\;\!\textrm{d}x$. The third example shows that in case of the Boltzmann entropy with $E(c)=c\log c$ the conditions do not hold and hence geodesic convexity fails.

### Example 6.3

(**Density function** $E(c)=c^m$) We have $\varepsilon _0(c) = c^m$, $\varepsilon _1(c) = m c^m$, and $\varepsilon _2(c)=m(m{-1}) c^m$, which gives the matrix

$$\begin{aligned} \mathbb {B}(c) = c^m \left( \begin{array}{cc} (m{-}1)\big (m-\frac{d-1}{d}\big ) &{} (m{-}1)\big (m-\frac{1}{2} \big )\\ (m{-}1)\big (m-\frac{1}{2} \big ) &{} m \big (m-\frac{1}{2} \big ) \end{array} \right) . \end{aligned}$$

The Hellinger condition $\mathbb {B}_{22}(c)\geqq 0$ holds for $m \not \in {]0,\frac{1}{2}[}$, while the McCann condition $\mathbb {B}_{11}(c)\geqq 0$ holds for $m \not \in {]\frac{d-1}{d},1[}$. Moreover, for $d\geqq 2$ the monotonicity condition $\varepsilon _1\geqq \varepsilon _0$ implies $m\geqq 1$.

Thus, the remaining cases are either $m\geqq 1$ or $d=1$ and $m\leqq 0$, and it remains to check $\det \mathbb {B}(c) \geqq 0$. An explicit calculation gives

$$\begin{aligned} \det \mathbb {B}(c)= (m{-}1)\left( m-\frac{1}{2} \right) \, \frac{(d{+}2)m -d}{2d}. \end{aligned}$$

Clearly, for $m\geqq 1$ we have $\det \mathbb {B}(c)\geqq 0$ for all space dimensions $d\in \mathbb {N}$. Moreover, $\det \mathbb {B}(c)< 0$ for $m\leqq 0$.

In summary, we obtain geodesic convexity if and only if $m\geqq 1$.

### Example 6.4

(**Density function** $E(c)=- c^q$) As in the previous example we have

$$\begin{aligned} \mathbb {B}(c) = c^q \left( \begin{array}{cc} (1{-}q)\big (q-\frac{d-1}{d}\big ) &{} (1{-}q)\big (q-\frac{1}{2} \big )\\ (1{-}q)\big (q-\frac{1}{2}\big ) &{} q \big (\frac{1}{2}-q \big ) \end{array} \right) . \end{aligned}$$

The Hellinger condition $\mathbb {B}_{22}(c)\geqq 0$ holds for $q \in [0,\frac{1}{2}]$, while the McCann condition $\mathbb {B}_{11}(c)\geqq 0$ holds for $q \in [\frac{d-1}{d},1]$, which also implies the monotonicity $\varepsilon _1\geqq \varepsilon _0$. With

$$\begin{aligned} \det \mathbb {B}(c)= (1{-}q) \,\big (\frac{1}{2}-q \big )\, \frac{(d{+}2)q -d}{2d} \, c^{2q}. \end{aligned}$$

we obtain the additional condition $q\geqq d/(d{+}2)$ and summarize that $E(c)=-c^q$ leads to a geodesically convex functional if and only if $q \in \big [\max \{\frac{d-1}{d},\frac{d}{d+2}\}, \frac{1}{2}\big ]$, which has solutions only for $d=1$ and $d=2$.

### Example 6.5

(**Boltzmann entropy**) As a negative example where the geodesic convexity fails, we consider the Boltzmann function $E(c)= c\log c$. We compute $\mathbb {B}_{22}(c)= \varepsilon _2(c)+\frac{1}{2}\varepsilon _1(c)= \frac{3}{2} c +\frac{1}{2}c\log c$, which shows that the necessary Hellinger condition fails. Moreover, considering the measures $\mu _0 = 0$ and $\mu _1=c\mathcal {L}^{d}$ for a non-negative density $c\in \textrm{L}^1(\Omega )$ we find that along the geodesic curve, given by $\mu (s)= s^2\mu _1$, we have

$$\begin{aligned} {\mathscr {E}}(\mu (s)) = \int _\Omega E(s^2 c)\;\!\textrm{d}x = s^2 {\mathscr {E}}(\mu _1) + 2s^2\log (s)\int _\Omega c\;\!\textrm{d}x, \end{aligned}$$

which is clearly not convex if $\int _\Omega c \;\!\textrm{d}x = \mu _1(\Omega )>0$.

Finally, we discuss a few examples where the density function *E* is not smooth. Note that the conditions in (1.25) form a closed cone. Moreover, as for convex functions, the supremum $E: c \mapsto \sup \{\, \widetilde{E}_\alpha (c) \, | \, \alpha \in A \,\}$ satisfies (1.25) if all $\widetilde{E}_\alpha$ do so.

### Example 6.6

(**Nonsmooth** *E*) In applications one is also interested in cases where *E* is nonsmooth. For example the case $$E_\kappa (c)=\kappa c$$ for $$c\in [0,c^*]$$ and $$E(c)=\infty $$ for $$c>c_*$$ is considered in [13]. Clearly, $$E_0$$ satisfies our assumptions (1.25) since $$N_E $$ only takes the values 0 and $$\infty $$ and the value 0 is taken on the convex set $$\gamma ^{d+2}\leqq c^*\rho ^d$$. Thus, $$E_\kappa $$ generates a functional $${\mathscr {E}}_\kappa = {\mathscr {E}}_0 + \kappa {\mathscr {M}} $$ that is geodesically $$2\kappa $$-convex.

A second example is given by $E(c)=\max \{ 0, c^2-c\}$. We first observe that $\widetilde{E}_1(c)=c$ and $\widetilde{E}_2(c)=c^2$ satisfy (1.25). Hence, $c\mapsto \max \{\widetilde{E}_1(c),\widetilde{E}_2(c)\}= E(c)+c$ satisfies (1.25) as well. Thus, we know that *E* generates a functional ${\mathscr {E}}$ that is at least geodesically $({-}2)$-convex. However, we may inspect the function $c\mapsto c^2-c$ in the region $c\geqq 1$ directly and find that *E* itself satisfies (1.25).

In practical applications, in particular for evolutionary variational inequalities as treated in [23], it is desirable to find the optimal $\lambda$ for the geodesic $\lambda$-convexity. So far, we have treated the case of geodesic 0-convexity and now return to the general case, which leads to the conditions

$$\begin{aligned} \mathbb {B}(c) \geqq \left( \begin{array}{cc}0&{}0\\ 0 &{}\lambda c/2 \end{array} \right) \quad \text { and } \quad (d{-}1)\big (\varepsilon _1(c) -\varepsilon _0(c)\big ) \geqq 0. \end{aligned}$$

The monotonicity condition is clearly independent of $\lambda$. The first equation still relies on the necessary McCann condition $\mathbb {B}_{11}(c)\geqq 0$. If this holds with strict inequality we see that the optimal $\lambda$ is characterized by

6.4 $$\begin{aligned} \lambda _\text {opt} = \inf \Big \{\, \frac{2 \det \mathbb {B}(c)}{c \mathbb {B}_{11}(c)} \; \Big | \; c>0 \,\Big \} . \end{aligned}$$

### Example 6.7

($d=1$ and $E(c)= c^2-c^{2/5}$) From the previous examples, we know that $E^2(c)=c^2$ and $E^{2/5}(c)=-c^{2/5}$ are both geodesically 0-convex, and we want to show that the sum is geodesically $\lambda$-convex for $\lambda >0$. As $\mathbb {B}$ is linear in *E* we have $\mathbb {B}(c)= c^2 \mathbb {B}^{(2)} + c^{2/5} \mathbb {B}^{(2/5)}$ with constant matrices $\mathbb {B}^{(2)}$ and $\mathbb {B}^{(2/5)}$ that are both strictly positive definite. Thus,

$$\begin{aligned} \ell (c):=\frac{2 \det \mathbb {B}(c)}{c\, \mathbb {B}_{11}(c)}>0 \quad \text { for all } c>0. \end{aligned}$$

Moreover, we find $\ell (c) \sim 2 c^{-3/5} \det \mathbb {B}^{(2/5)}/ \mathbb {B}^{(2/5)}_{11}$ for $c \approx 0$ and $\ell (c) \sim 2 c \det \mathbb {B}^{(2)}/ \mathbb {B}^{(2)}_{11}$ for $c \gg 1$. Thus, by compactness $\lambda _\textrm{opt} = \inf \big \{\, \ell (c) \, \big | \, c>0 \,\big \}$ is strictly positive.

Numerically, we find $\lambda _\textrm{opt} \approx 0.638$ which is attained at $c_*\approx 0.0319$.

### Remark 6.8

(**Geodesic convexity via the Otto calculus**) Following the key ideas in [12, 31] a formal calculus for reaction-diffusion systems was developed in [25]. It uses the dynamical formulation in Subsection 2.1.1 and the associated Onsager operator $\mathbb {K}(c)\xi = -\alpha \mathop {\textrm{div}}(c\nabla \xi ) + \beta c \xi$ to characterize the geodesic $\lambda$-convexity of the functional ${\mathscr {E}}$ by calculating the quadratic form $M(c,\cdot )$ (contravariant Hessian of ${\mathscr {E}}$):

$$\begin{aligned} M(c,\xi )=\langle \xi ,\textrm{D}{\textbf{V}}(c) \mathbb {K}(c)\xi \rangle - \frac{1}{2} \textrm{D}_c\langle \xi , \mathbb {K}(c)\xi \rangle [{\textbf{V}}(c)] \quad \text {with } {\textbf{V}}(c)=\mathbb {K}(c) \textrm{D}{\mathscr {E}}(c). \end{aligned}$$

Then, one needs to show the estimate $M(c,\xi ) \geqq \lambda \langle \xi ,\mathbb {K}(c)\xi \rangle$.

Following the methods in [25, Sect. 4], for $c\in \textrm{C}^0_\textrm{c}(\Omega )$ and smooth $\xi$ we obtain

$$\begin{aligned} M(c,\xi )&=\int _\Omega \bigg [ \alpha ^2 \Big ( \big ( A(c){-}H(c)\big ) (\Delta \xi )^2 + H(c)\big | \textrm{D}^2 \xi \big |^2 \Big ) \\&\hspace{5em}+ \alpha \beta \Big (B_1(c) |\nabla \xi |^2 + B_2(c)\xi \Delta \xi \Big ) +\beta ^2 B_3(c) \xi ^2 \bigg ] \;\!\textrm{d}x,\\ \text {where }&A(c)= \varepsilon _2(c), \quad H(u)= \varepsilon _1(c) - \varepsilon _0(c), \quad B_1(c)=\frac{3}{2} \varepsilon _1(c)- \varepsilon _0(c),\\&B_2(c)=-2\varepsilon _2(c) + \varepsilon _1(c)- \varepsilon _0(c),\quad B_3(c)= \varepsilon _2(c)+ \frac{1}{2}\varepsilon _1(c). \end{aligned}$$

Analyzing the condition $M(c,\xi )\geqq \lambda \langle \xi , \mathbb {K}(c)\xi \rangle$, we find the conditions

6.5 $$\begin{aligned}{} & {} \forall c\geqq 0:\quad (d{-}1)H(c)\geqq 0, \nonumber \\{} & {} B_1(c)\geqq \frac{\lambda }{\beta } c, \ \ \left( \begin{array}{cc}\!\! A(c)-\frac{d-1}{d} H(c)&{} \frac{1}{2} B_2(c)\\ \frac{1}{2} B_2(c)&{} B_3(c)-\frac{\lambda }{\beta } c \!\!\end{array} \right) \geqq 0, \end{aligned}$$

which for $\lambda =0$ give the same conditions as $\mathbb {B}(c)\geqq 0$, see Proposition 6.1. Note that the middle estimate in (6.5) follows from the first and the third estimates because of

$$\begin{aligned} \textstyle B_1(c) = \frac{3}{2}\varepsilon _1 - \varepsilon _0 = \frac{d-1}{d} (\varepsilon _1{-}\varepsilon _0) + \frac{1}{2d} \big ((d{+}2)\varepsilon _2{-}2\varepsilon _0\big ). \end{aligned}$$

## Proof of Geodesic Convexity of ${\mathscr {E}}$

In this section, we finally prove the necessity and sufficiency of the conditions for geodesic convexity of functionals ${\mathscr {E}}$ on $\mathcal {M}(\Omega )$ in (1.25), where we now allow for a general closed and convex domain $\Omega \subset \mathbb {R}^d$. In order to keep the arguments clear, we first restrict ourselves to absolutely continuous measures $\mu _0$ and $\mu _1$. Thus, by Corollary 5.5 the connecting geodesic curves are also absolutely continuous, and we can rewrite ${\mathscr {E}}$ along the latter in the form

$$\begin{aligned} {\mathscr {E}}(\mu _t){} & {} = \int _\Omega E(c(t,y))\;\!\textrm{d}y = \int _\Omega e(t,x) \;\!\textrm{d}x, \quad \text {where }\\ e(t,x){} & {} = \delta (t,x)\;\!E\Big ( c_*(x) \frac{\alpha (t,x)}{\delta (t,x)}\Big ). \end{aligned}$$

The general case will then be treated by using an approximation argument.

Under the assumption that *E* is twice differentiable in the interior of its domain, we show that for $\mu _0$-a.a. $x\in \Omega$ the function $t\mapsto e(t,x)$ is convex. Since $\alpha (\cdot ,x)$ and $\delta (\cdot )$ are analytic functions on [0, 1], we can show convexity in this case by establishing $\ddot{e}(t,x)\geqq 0$. For this, we can fix $x\in \Omega$, drop the dependence on *x* for notational convenience, and set

7.1 $$\begin{aligned}{} & {} e(t)= \delta (t)E\big (c_*\frac{\alpha (t)}{\delta (t)}\big )= N_E \big ( \rho (t),\gamma (t) \big ) \quad \text {with }\rho :=(c_*\alpha )^{1/2}\delta ^{1/d}, \nonumber \\{} & {} \gamma :=(c_*\alpha )^{1/2}, \end{aligned}$$

and $N_E$ from (1.25a). Now, the classical chain rule implies the relation

7.2 $$\begin{aligned} \ddot{e}= \Big \langle \left( {\begin{array}{c}{\dot{\rho }}\\ {\dot{\gamma }}\end{array}}\right) , \textrm{D}^2N_E(\rho ,\gamma ) \left( {\begin{array}{c}{\dot{\rho }}\\ {\dot{\gamma }}\end{array}}\right) \Big \rangle +\partial _\rho N_E(\rho ,\gamma )\ddot{\rho }+ \partial _\gamma N_E(\rho ,\gamma )\ddot{\gamma }. \end{aligned}$$

The aim is to show $\ddot{e}(t)\geqq 0$ for all $t\in [0,1]$. By the convexity of $N_E$ it suffices to treat the last two terms.

For this we exploit the curvature estimates (5.12) on $\ddot{\gamma }$ and $\ddot{\rho }$ as well as the monotonicities in (1.25c) and Proposition 6.2.

### Usage of the Curvature Estimates

We first show that it is sufficient to use the curvature estimates

7.3 $$\begin{aligned} \frac{\ddot{\gamma }}{\gamma }\geqq 0 \quad \text {and} \quad \left\{ \begin{array}{cl} \displaystyle \frac{\ddot{\rho }}{\rho }\leqq \big (1{-}\frac{4}{d^2}\big ) \!\;\frac{\ddot{\gamma }}{\gamma }&{} \text { for } d\geqq 2, \\ \displaystyle \frac{\ddot{\rho }}{\rho }= \big (1{-}\frac{4}{d^2}\big ) \!\;\frac{\ddot{\gamma }}{\gamma }&{}\text { for } d=1. \end{array}\right. \end{aligned}$$

In particular, the equality condition for $d=1$ is different from the inequality conditions for $d\geqq 2$. This will be used to compensate for the missing monotonicity of $N_E$ in (1.25c) in the case $d= 1$.

Below we will see that the curvature estimates (7.3) are *necessary* to complete our proof. Note that they are implied by the curvature estimates derived in Proposition 5.7. In fact, both coincide for $d=1$, while for $d\geqq 2$ the former are strictly weaker as the latter because of $1-4/d < 1-4/d^2$.

#### Proposition 7.1

($\ddot{e}\geqq 0$ via curvature estimates) Assume that $N_E$ satisfies (1.25) and that $t\mapsto (\rho (t),\gamma (t))$ satisfies (7.3), then $\ddot{e}\geqq 0$ in (7.2).

#### Proof

As the first term (involving $\textrm{D}^2 N_E$) on the right-hand side of (7.2) is non-negative, we only have to show that the last two terms have a non-negative sum. For this we rearrange terms as follows:

$$\begin{aligned}&\partial _\rho N_E(\rho ,\gamma ) \ddot{\rho } {+} \partial _\gamma N_E(\rho ,\gamma ) \ddot{\gamma }\\&\qquad = \big (\!{-}\rho \partial _\rho N_E\big ) \Big (\! \big (1{-}\frac{4}{d^2}\big ) \frac{\ddot{\gamma }}{\gamma }-\frac{\ddot{\rho }}{\rho }\Big ) + \Big (\!\gamma \partial _\gamma N_E {+} \big (1{-}\frac{4}{d^2}\big ) \rho \partial _\rho N_E \Big )\frac{\ddot{\gamma }}{\gamma }\,. \end{aligned}$$

The right-hand side is the sum of two products, both of which are non-negative. Indeed, the first product equals 0 in the case $d=1$ independently of the sign of $\partial _\rho N_E$, because the second factor is 0. In the case $d\geqq 2$ both factors are non-negative (using $\partial _\rho N_E\leqq 0$ and the second curvature estimate in (7.3)), so the first product is non-negative again.

In the second product both terms are non-negative by Proposition 6.2(B) and the first curvature estimate in (7.3). Thus, $\ddot{e}\geqq 0$ in (7.2) is proved. $\quad \square$

### The Main Results on Geodesic $\lambda$-Convexity

We are now ready to establish our main result on the geodesic convexity of functionals ${\mathscr {E}}$ given in terms of a density *E*. We now make our general assumptions of *E* precise.

7.4a $$\begin{aligned} \begin{aligned}&E:{[0,\infty [}\rightarrow \mathbb {R}\cup \{\infty \} \text { is lower semi-continuous, convex,}\\&E(0)=0, \text { and there exists } c_\circ >0 \text { such that } E(c_\circ ) < \infty . \end{aligned} \end{aligned}$$

We also want to include the case that *E* is not necessarily superlinear, so we introduce the recession constant

$$\begin{aligned} E'_\infty := \lim _{c\rightarrow \infty } \frac{1}{c} E(c) \in \mathbb {R}\cup \{\infty \}. \end{aligned}$$

The case $E'_\infty =\infty$ is the superlinear case where the functional ${\mathscr {E}}(\mu )$ is always $+\infty$, if $\mu$ has a singular part, i.e. $\mu ^\perp \ne 0$ in the decomposition $\mu = c\mathcal {L}^{d} + \mu ^\perp$ with $\mu ^\perp \perp \mathcal {L}^{d}$.

We introduce a closed (convex) domain $\Omega \subset \mathbb {R}^d$, and we consider the set of measures $\mu$ with support contained in $\Omega$, which we identify with $\mathcal {M}(\Omega )$. In the case that the right derivative $\displaystyle E'_0:=\lim _{c\downarrow 0}\frac{1}{c} E(c)$ of *E* at 0 is not finite, we further have to impose that $\Omega$ has finite Lebesgue measure. Therefore, we will assume that

7.4b $$\begin{aligned} \begin{aligned}&\Omega \text { is a closed convex set with nonempty interior and}\\&{\Omega \, \text { is also bounded, i.e.~}\mathcal {L}^{d}(\Omega )<\infty , if E_0'=-\infty .} \end{aligned} \end{aligned}$$

Thus, the functionals ${\mathscr {E}}$ are defined as follows:

7.5 $$\begin{aligned} \hspace{-2em} {\mathscr {E}}(\mu )=\int _{\Omega } E(c(x)) \;\!\textrm{d}x + E'_\infty \mu ^\perp (\Omega )\quad \text {for}\quad \mu =c\mathcal {L}^{d}{+}\mu ^\perp \text { with } \mu ^\perp \perp \mathcal {L}^{d}. \end{aligned}$$

It is well known that (7.4) guarantees that ${\mathscr {E}}$ is a weakly lower semi-continuous functional on $\mathcal {M}(\Omega )$. In particular, condition (7.4b) is necessary to guarantee that the negative part $x\mapsto \min \{ E(c(x)),0\}$ is integrable, because for $c\in \textrm{L}^1(\Omega )$ the functions $x\mapsto -\sqrt{c(x)}$ may not lie in $\textrm{L}^1(\Omega )$. We refer to Example 7.4 for a case where (7.4b) can be avoided by using a confining potential.

We are now in the position to formulate our main result on the geodesic $\lambda$-convexity of integral functionals ${\mathscr {E}}$ on the Hellinger–Kantorovich space $(\mathcal {M}(\Omega ),\textsf{H}\!\!\textsf{K})$. The proof consists of three steps. First, we assume that *E* is twice continuously differentiable in its domain. Restricting to geodesic curves connecting absolutely continuous measures, we can use the above differentiable theory giving $\ddot{e}\geqq 0$. In Step 2, we generalize to possibly non-differentiable density functions *E*, but keep absolutely continuous measures. For smoothing a given *E*, we use that whenever *E* solves the conditions (1.25) and (7.4) then $c\mapsto E(rc)$ does so for each $r\in [0,1]$. With a multiplicative convolution we construct a smooth $E_\delta$ to which Step 1 applies. Finally, Step 3 handles the case where $\mu _0^\perp$ or $\mu _1^\perp$ are non-zero by a standard approximation argument of general measures using absolutely continuous measures.

#### Theorem 7.2

(Geodesic convexity of ${\mathscr {E}}$) Assume that $E:{[0,\infty [}\rightarrow \mathbb {R}\cup \{\infty \}$ and $\Omega \subset \mathbb {R}^d$ satisfy (7.4a) and (7.4b), respectively. If for a $\lambda _*\in \mathbb {R}$ the function

$$\begin{aligned} N_{\lambda _*,E}(\rho ,\gamma ):= \big (\frac{\rho }{\gamma }\big )^d \,E\Big ( \frac{\gamma ^{d+2}}{\rho ^d} \Big ) - \frac{\lambda _*}{2} \gamma ^2, \quad \text { for }\rho ,\gamma >0, \end{aligned}$$

satisfies the conditions (1.25b) and (1.25c), then the functional ${\mathscr {E}}$ defined in (7.5) is geodesically $\lambda _*$-convex on $( \mathcal {M}(\Omega ), \textsf{H}\!\!\textsf{K})$.

#### Proof

Without loss of generality, we set $\lambda _*=0$ throughout the proof and shortly write $N_E=N_{\lambda _*,E}$.

*Step 1: The smooth and absolutely-continuous case.* We first assume that *E* is twice continuously differentiable in the interior ${]0,c_E[}$ of its domain and that the measures $\mu _0$ and $\mu _1$ are absolutely continuous with respect to $\mathcal {L}^{d}$, i.e. $\mu _j= c_j\mathcal {L}^{d}$ for $c_j \in \textrm{L}^1(\Omega )$.

We fix $s\in (0,1)$ adopting the notation of Corollary 5.5. Then, the geodesic curve $t \mapsto \mu _t=c(t,\cdot )\mathcal {L}^{d}$ satisfies

$$\begin{aligned} {\mathscr {E}}(\mu _t)=\int _\Omega E(c(t,y))\;\!\textrm{d}y= \int _\Omega E\big (c_s(x)\frac{\alpha _s(t,x)}{\delta _s(t,x)} \big ) \delta _s(t,x)\;\!\textrm{d}x = \int _\Omega e(t,x) \;\!\textrm{d}x \end{aligned}$$

with $e(t,x)=N_E(\rho (t,x),\gamma (t,x))$ as above. We want to show that for a.a. $x\in \Omega$ the function $t\mapsto e(t,x)$ is convex.

As shown in Theorem 5.8 the functions $t\mapsto \widetilde{c}(t,x)=c_0(x)\frac{\alpha (t,x)}{\delta (t,x)}= \gamma (t,x)^{d+2}/\rho (t,x)^d$ are either constant or strictly convex. If the function $\widetilde{c}(\cdot ,x)$ is constant then either $c_0(x)=0$ or $(\rho (\cdot ,x),\gamma (\cdot ,x))$ is constant. In both cases, $e(\cdot ,x)$ is constant as well, and hence convex.

In the strictly convex case, the values of $\widetilde{c}(t,x)$ for $t\in {]0,1[}$ lie in the interior of the domain of *E*, where *E* is twice differentiable. Hence, combining Propositions 7.1 and 5.7 shows that $t\mapsto e(t,x)$ is convex for a.a. $x\in \Omega$. Since integration over $\Omega$ maintains convexity we conclude that $t \mapsto {\mathscr {E}}(\mu _t)$ is convex, too.

*Step 2: The nonsmooth but absolutely-continuous case.* We still assume $\mu _j=c_j\mathcal {L}^{d}$, but now consider an *E* that is not necessarily twice differentiable, but still satisfies (7.4). We choose a function $\chi \in \textrm{C}^\infty _\textrm{c}(\mathbb {R})$ satisfying $\chi (r)\geqq 0$, $\int _{-2}^{-1} \chi (r)\;\!\textrm{d}r =1$, and $\chi (r)=0$ for $r\not \in [-2,-1]$. Now for $\delta \in {]0,1/2[}$ we define the smoothings

$$\begin{aligned} E_\delta (c) = \int _0^1 \chi _\delta (r) E(rc) \;\!\textrm{d}r, \quad \text {where } \chi _\delta (r)=\frac{1}{\delta }\chi \big (\frac{1}{\delta }(r{-}1)\big ). \end{aligned}$$

Hence, $\chi _\delta$ has support in $[1{-}2\delta , 1{-}\delta ]$. If the closure of the domain of *E* is $[0,c_E]$, then $E_\delta$ is well-defined and $\textrm{C}^\infty$ on ${]0,c_E/(1{-}\delta )[}$. Moreover, for all $c\in [0,c_E]$ we have $E_\delta (c)\rightarrow E(c)$ for $\delta \searrow 0$. We easily check, that $E_\delta$ still satisfies the assumption (1.25) and (7.4). Moreover, $E_\delta (c)$ can be estimated by *E*(*c*) via

7.6 $$\begin{aligned} \exists \, K>0\ \forall \, \delta \in {]0,1/4[} \ \forall \, c\geqq 0: \qquad |E_\delta (c) | \leqq K\big (c + |E(c)| \big ). \end{aligned}$$

To see this, we first consider the largest interval ${[0,c_1[}$ on which *E* is non-increasing. Then $0=E(0)\geqq E_\delta (c) \geqq E(c)$ which implies (7.6) with $K=1$. If $c_1=\infty$ then we are done. If $c_1<\infty$, then *E* starts to increase and there exists $c_2\in {[c_1,\infty [}$ with $E(c)\geqq 0$ for $c\geqq c_2$. Using the construction of $E_\delta$, we obtain for all $c\geqq 2c_2\geqq c_2/(1{-}2\delta )$ the lower bound $E_\delta (c)\geqq 0$. Using (6.3) we easily get $E_\delta (c) \leqq E(c)$.

It remains to cover the case $c\in [c_1,3c_2]$. If $c_1=0$ then $E(c)\geqq 0$ for all *c*, which means $c_2=0$ as well, then (7.6) follows immediately from the above arguments. If $c_1>0$, a uniform continuity argument gives the estimate $|E_\delta (c)-E(c)| \leqq M$ for $c\in [c_1,3c_2]$. Then, choosing $K=M/c_1$ provides (7.6).

With this preparation, Lebesgue’s dominated convergence theorem implies

$$\begin{aligned} \mu{} & {} =c\mathcal {L}^{d} \text { with }c\in \textrm{L}^1(\Omega ) \ \text {and} \ {\mathscr {E}}(\mu )<\infty \\{} & {} \Longrightarrow \quad \int _\Omega E_\delta (c(x))\;\!\textrm{d}x \rightarrow {\mathscr {E}}(\mu ) \text { as }\delta \downarrow 0. \end{aligned}$$

Taking any constant-speed geodesic $[0,1] \ni t\mapsto \mu _t = c(t,\cdot )\mathcal {L}^{d}$, we know by Step 1 that the curves

$$\begin{aligned} \overline{e}_\delta : t \mapsto \int _\Omega E_\delta (c(t,x)) \;\!\textrm{d}x \end{aligned}$$

are convex. As $\overline{e}_\delta (t)\rightarrow {\mathscr {E}}(\mu _t)$ we conclude that $t \mapsto {\mathscr {E}}(\mu _t)$ is convex on [0, 1].

*Step 3: Pure growth.* The curve $t \mapsto t^2 \mu _1$ is the unique geodesic connecting $\mu _0=0$ and $\mu _1$. Using the Lebesgue decomposition $\mu _1= c_1\mathcal {L}^{d} + \mu ^\perp$ we see that

$$\begin{aligned} t\mapsto {\mathscr {E}}(\mu _t) = {\mathscr {E}}(t^2c_1\mathcal {L}^{d}) + t^2 E_\infty ' \mu ^\perp , \end{aligned}$$

is convex on [0,1] by Step 2 for the first term and by $E_\infty '\geqq 0$. The nonnegativity of $E_\infty '= \lim _{c\rightarrow \infty } E(c)/c$ follows from (7.4a) and Proposition 6.2(A), namely for $c\geqq c_\circ$ we have

$$\begin{aligned} \frac{1}{c} E(c) = \frac{1}{c^{d/(d+2)}} \,c^{-2/(d+2)} E(c) \overset{\text {(i)}}{\geqq }\frac{1}{c^{d/(d+2)}} \,c_\circ ^{-2/(d+2)} E(c_\circ ) \rightarrow 0 \ \text { for } c \rightarrow \infty . \end{aligned}$$

*Step 4: The general case allowing for singular measures.* Singular measures can only occur for *E* with sublinear growth. Hence, we assume $E'_\infty \in \mathbb {R}$ from now on. In particular ${\mathscr {E}}$ is finite everywhere, and using $E(c)\leqq E_\infty ' c$ we have ${\mathscr {E}}(\mu ) \leqq E_\infty '\mu (\Omega )$.

As in Corollary 5.6, we consider an arbitrary geodesic $(\mu _t)_{t\in [0,1]}$ connecting $\mu _0$ and $\mu _1$. For a fixed $s \in (0,1)$, we decompose $\mu _s$ as $\mu _s^\textrm{a}+\mu _s^\perp$. Then, $\mu _t= \widetilde{\mu }_t + \widehat{\mu }_t$ splits into two geodesics with disjoint supports and $\widetilde{\mu }_s=\mu _s^\textrm{a}$ and $\widehat{\mu }_s= \mu _s^\perp$, see Corollary 5.6. Moreover, we have $\widehat{\mu }_t \perp \mathcal {L}^{d}$ and $\widetilde{\mu }_t \ll \mathcal {L}^{d}$ for all $t\in (0,1)$. This implies the relation

$$\begin{aligned} {\mathscr {E}}(\mu _t)={\mathscr {E}}(\widetilde{\mu }_t)+{\mathscr {E}}(\widehat{\mu }_t)= {\mathscr {E}}(\widetilde{\mu }_t)+E_\infty '\widehat{\mu }_t (\Omega ). \end{aligned}$$

Since $(\widehat{\mu }_t)_{t\in [0,1]}$ is a geodesic and the total mass functional ${\mathscr {M}}(\mu )= \mu (\Omega )$ is convex (see (1.21)) and $E_\infty '\geqq 0$, the last term $t\mapsto E_\infty '\widehat{\mu }_t(\Omega )$ is convex. Hence, it is sufficient to check the convexity of $t\mapsto {\mathscr {E}}(\widetilde{\mu }_t)$.

Since $\widetilde{\mu }_t\ll \mathcal {L}^{d}$ for all $t\in (0,1)$, the function $t \mapsto {\mathscr {E}}(\widetilde{\mu }_t)$ is convex in the open interval (0, 1) by Step 2. Hence, to show convexity on [0, 1] it is sufficient to check that

$$\begin{aligned} \limsup _{t\downarrow 0}{\mathscr {E}}(\widetilde{\mu }_t) \leqq {\mathscr {E}}(\widetilde{\mu }_0) \qquad \text {and} \qquad \limsup _{t\uparrow 1}{\mathscr {E}}(\widetilde{\mu }_t)\leqq {\mathscr {E}}(\widetilde{\mu }_1), \end{aligned}$$

because $\textsf{H}\!\!\textsf{K}$ convergence implies weak convergence and ${\mathscr {E}}$ is weakly l.s.c.

Let us focus on the limit $t\downarrow 0$ as the limit $t\uparrow 1$ is completely analogous. The problem is that $\widetilde{\mu }_t \ll \mathcal {L}^{d}$ for $t\in (0,1)$ only, but $\widetilde{\mu }_0$ may have a singular part. Hence, we forget the decomposition $\mu _t=\widetilde{\mu }_t+\widehat{\mu }_t$ and use a different one. Before that, we restrict to the case $\mu _0(\Xi ^+)=0$ because on $\Xi ^+$ we have pure growth and this case is covered by Step 3.

Now, we exploit the Lebesgue decomposition of $\mu _0=\mu _0^\textrm{a}+\mu _0^\perp$ at $t=0$ and consider two disjoint Borel sets $A,B \subset \Omega \setminus \Xi ^+$ such that  and . We define the corresponding disjoints sets $A_t:=\varvec{T}_{t\rightarrow 0}^{-1}(A)$ and $B_t:=\varvec{T}_{t\rightarrow 0}^{-1} (B)$ as well as the measures  and . By Theorem 5.4, we obtain two geodesics $\nu _t^A$, $\nu _t^B$ concentrated on disjoint sets giving ${\mathscr {E}}(\mu _t)= {\mathscr {E}}(\nu _t^A)+{\mathscr {E}}(\nu _t^B)$. Since $\nu _t^A\ll \mathcal {L}^{d}$ for every $t\in [0,1)$ we deduce that $t\mapsto {\mathscr {E}}(\nu _t^A)$ is convex up to 0 by Step 2. Concerning ${\mathscr {E}}(\nu _t^B)$, we use ${\mathscr {E}}(\mu )\leqq E'_\infty \mu (\Omega )$ and find

$$\begin{aligned} \limsup _{t\downarrow 0}{\mathscr {E}}(\nu _t^B) \leqq E_\infty '\limsup _{t\downarrow 0}\nu _t^B(\Omega ) = E_\infty '\nu _0^B(\Omega ) ={\mathscr {E}}(\nu _0^B), \end{aligned}$$

where we exploited $\nu _0^B\perp \mathcal {L}^{d}$ in the last identity.

This finishes the proof of the main theorem $\quad \square$

The next result is a direct consequence of the main result by using the results of Examples 6.3 and 6.4, respectively. In particular, this establishes the result announced in [13, Thm. 2.14].

#### Corollary 7.3

(Power-law functionals) Assume that $\Omega \subset \mathbb {R}^d$ and $E:{[0,\infty [}\rightarrow \mathbb {R}$ satisfy (7.4) and let ${\mathscr {E}}$ be defined via (7.5).

1. If $E(c)=c^m$ with $m\geqq 1$, then ${\mathscr {E}}$ is geodesically convex on $( \mathcal {M}(\Omega ), \textsf{H}\!\!\textsf{K})$.
2. If ${\mathcal {L}}^{d}(\Omega )<\infty$, $d\in \{1,2\}$, and $E(c)=-c^q$ with $d/(d{+}2) \leqq q \leqq 1/2$, then ${\mathscr {E}}$ is geodesically convex on $( \mathcal {M}(\Omega ), \textsf{H}\!\!\textsf{K})$.

#### Example 7.4

We have seen above that the density $E(c)=-\sqrt{c}$ produces a geodesically convex functional in dimensions $d=1$ and 2, if $\mathcal {L}^{d}(\Omega )<\infty$. The restriction of finite volume for $\Omega$ can be dropped by using a confining potential *V* as follows: let

$$\begin{aligned} {\mathscr {E}}_{1/2,V}(\mu ) = \int _{\mathbb {R}^d} \big ({-}\sqrt{c(x)}\big ) \;\!\textrm{d}x + \int _{\mathbb {R}^d} V \;\!\textrm{d}\mu \ \text { for } \mu =c\mathcal {L}^{d} + \mu ^\perp , \end{aligned}$$

where $V\in \textrm{C}(\mathbb {R}^d)$ satisfies for $m >d$ and $A\in \mathbb {R}$ the lower bound $V(x)\geqq a_0|x|^m -A$ on $\mathbb {R}^d$. Then it is easy to see that ${\mathscr {E}}_{1/2,V}$ is well-defined and weakly lower semi-continuous.

Moreover, in [26, Prop. 20] it was shown for a continuous $V:\mathbb {R}^d \rightarrow \mathbb {R}$ with $\inf V> - \infty$ that the linear mapping $\mu \mapsto \int _{\mathbb {R}^d} V \;\!\textrm{d}\mu$ is geodesically $\lambda _V$-convex on $(\mathcal {M}(\Omega ),\textsf{H}\!\!\textsf{K})$ if and only if the mapping $\widetilde{V}:[x,r]\mapsto r^2 V(x)$ is geodesically $\lambda _V$-convex on the metric cone space $(\mathfrak {C},\textsf{d}_\mathfrak {C})$. For smooth *V*, this amounts to the estimate

$$\begin{aligned} \left( \begin{array}{cc} \nabla ^2 V(x)+2V(x)\mathbb {I}_d&{}\nabla V(x)\\ \nabla V(x)^\top &{} 2V(x) \end{array} \right) \geqq \lambda _V\mathbb {I}_{d+1}. \end{aligned}$$

Thus, for *V* satisfying both of the above assumptions, the functional ${\mathscr {E}}_{1/2,V}$ is geodesically $\lambda _V$-convex on $(\mathcal {M}(\mathbb {R}^2), \textsf{H}\!\!\textsf{K})$ for $d\in \{1,2\}$. For $d=1$ we may choose $V(x)= \alpha + \beta |x|^2$ with $\beta >0$ and obtain $\lambda _V=2\alpha$.

### Necessity of the Conditions on *E*

Theorem 7.2 states that the conditions (1.25) and (7.4) on the density $E:[0,\infty ) \rightarrow (-\infty ,+\infty ]$ are sufficient for the geodesic convexity of the integral functional ${\mathscr {E}}$. We finally show that the conditions are also sufficient. To simplify the analysis we restrict ourselves to the smooth case where $E:\textrm{dom}(E)\rightarrow \mathbb {R}$ lies in $\textrm{C}^2$. Thus, we can obtain conditions by differentiation along suitably chosen geodesic curves. For this, the characteristic equations (4.62) derived in Theorem 4.8 will be the main tool.

#### Theorem 7.5

(Necessity of conditions on *E*) Consider a closed, convex domain $\Omega \subset \mathbb {R}^d$ with nonempty interior and a density function $E:[0,\infty ) \rightarrow (-\infty ,+\infty ]$ such that (7.4) holds and that *E* is $\textrm{C}^2$ on the interior of its domain. If the induced functional ${\mathscr {E}}:{\mathcal {M}}(\Omega ) \rightarrow (-\infty ,+\infty ]$ defined in (7.5) is geodesically convex on $({\mathcal {M}}(\Omega ),\textsf{H}\!\!\textsf{K})$, then *E* satisfies the conditions (1.25).

#### Proof

We first observe that it is sufficient to show that for $(\rho _*,\gamma _*)$ with $c_*=\gamma _*^{2+d}/\rho _*^d\in {\text {int}}(\textrm{dom}(E))$ we have the inequalities

7.7 $$\begin{aligned} \textrm{D}^2 N_E(\rho _*,\gamma _*)\geqq 0 \quad \text {and} \quad (d{-}1) \partial _\rho N_E(\rho _*,\gamma _*)\leqq 0. \end{aligned}$$

By the scaling properties of $N_E(\rho ,\gamma )=(\rho /\gamma )^d E(\gamma ^{2+d}/\rho ^d)$ it is sufficient to look at the case $(\rho _*,\gamma _*)=(c_*^{1/2},c_*^{1/2})$.

The main idea is to construct suitable geodesic curves $\mu _t$ such that the convexity of $t\mapsto {\mathscr {E}}(\mu _t)$ gives the desired inequality. For this we choose a point $x_*\in {\text {int}}(\Omega )$ and $r_*>0$ such that $B_{3r_*}(x_*)\subset \Omega$. Without loss of generality we assume $x_*=0$ and write $B_r$ in place of $B_r(x_*)$ for $r\in (0,3r_*]$.

We further choose an $s \in (0,1)$ and a smooth function $\xi _s \in \textrm{C}^3(B_{3r_*})$. Then, there exists an $\varepsilon >0$ such that there is a unique smooth solution $\xi :(s{-}\varepsilon , s{+}\varepsilon )\times B_{2r_*}\rightarrow \mathbb {R}$ of the Hamilton–Jacobi equation (2.4). With this $\xi _t= \xi (t,\cdot )$ and $r\in (0,r_*)$ we can construct a geodesic curveof absolutely continuous measures $\mu _t =c^{(r)}(t,\cdot ) \mathcal {L}^{d}$, see (5.6a) in Corollary 5.5. If necessary $\varepsilon$ needs to be reduced to avoid mass flowing outside $B_{2r_*}$. For this geodesic we have

$$\begin{aligned} {\mathscr {E}}(\mu _t^{(r)})=\int _\Omega E(c^{(r)}(t,y))\;\!\textrm{d}y = \int _{B_r} e(t,x) \;\!\textrm{d}x \quad \text {with } e(t,x)= N_E(\rho (t,x),\gamma (t,x)), \end{aligned}$$

where $\gamma (t,x)=c_*^{1/2}q_{s\rightarrow t}(x)$ and $\rho (t,x)= c_*^{1/2}q_{s\rightarrow t}(x) \big (\delta _{s\rightarrow t}(x)\big ){}^{1/d}$ with *q* and $\delta$ from (4.62). Note that *q*, $\delta$, and *e* do not depend on *r*, cf. Theorem 5.4.

By the smoothness of $\xi$, and hence of $\rho$ and $\gamma$, we may pass to the limit $r\downarrow 0$ in the convex functions $t \mapsto \frac{1}{\mathcal {L}^{d}(B_r)} {\mathscr {E}}(\mu _t^{(r)})= \frac{1}{\mathcal {L}^{d}(B_r)} \int _{B_r} e(t,x) \;\!\textrm{d}x$. Thus, the limit

$$\begin{aligned} t \mapsto e(t,0) = N_E(\rho (t,0),\gamma (t,0)) \quad \text {is convex on } (s{-}\varepsilon , s{+}\varepsilon ). \end{aligned}$$

In particular, the second derivative is non-negative which means that

$$\begin{aligned} 0{} & {} \leqq \ddot{e}(s,0) = \textrm{D}^2 N_E(c_*^{1/2},c_*^{1/2})\Big [{\displaystyle \left( {\begin{array}{c}{\dot{\rho }}\\ {\dot{\gamma }}\end{array}}\right) }, {\displaystyle \left( {\begin{array}{c}{\dot{\rho }}\\ {\dot{\gamma }}\end{array}}\right) } \Big ]\\{} & {} \qquad + \partial _\rho N_E(c_*^{1/2},c_*^{1/2} ) \ddot{\rho }+ \partial _\gamma N_E(c_*^{1/2},c_*^{1/2} ) \ddot{\gamma }, \end{aligned}$$

where now ${\dot{\rho }}=\partial _t\rho (s,0)$, ${\dot{\gamma }}$, $\ddot{\rho }$, and $\ddot{\gamma }$ are given by (4.62) and (5.13) in terms of $\xi$ only:

$$\begin{aligned}{} & {} \frac{{\dot{\rho }}}{c_*^{1/2}} =2\xi _s{+}\Delta \xi _2, \quad \frac{{\dot{\gamma }}}{c_*^{1/2}}= 2\xi _s, \\{} & {} \frac{\ddot{\rho }}{c_*^{1/2}} =\frac{(\Delta \xi _s)^2 {-} d|\textrm{D}^2\xi _s|^2}{d^2} + \frac{d{-}4}{d} |\nabla \xi _s|^2,\\{} & {} \frac{\ddot{\gamma }}{c_*^{1/2}}=|\nabla \xi _s|^2. \end{aligned}$$

there $\xi _s$ and its derivatives are evaluated at $x=x_*=0$.

To obtain the convexity of $N_E$ we can now choose the functions $\xi _s$ such that $\ddot{\rho }=\ddot{\gamma }=0$, which is the case for $\xi _s(x) = \alpha + \beta |x|^2$, which implies $\nabla \xi _s(0)=0$, $\Delta \xi _s(0)= 2d\beta$, and $|\textrm{D}^2\xi _s|^2=d\beta ^2$. Moreover, ${\dot{\rho }}$ and ${\dot{\gamma }}$ can be chosen arbitrarily by adjusting $\alpha ,\,\beta \in \mathbb {R}$. Thus, $\textrm{D}^2N\geqq 0$ is established.

To prove the second estimate in (7.7) we may assume $d\geqq 2$, as there is nothing to show for $d=1$. Choosing the function $\xi _s=\alpha (x_1^2-x_2^2)$ we obtain ${\dot{\rho }}={\dot{\gamma }}=\ddot{\gamma }=0$ and $\ddot{\rho }= -8c_*^{1/2} \alpha ^2/d$. This implies $\partial _\rho N_E\leqq 0$ and the theorem is established. $\quad \square$

### A More Direct Sufficiency Proof for $2\leqq d\leqq 4$

The above proof of Theorem 7.2 strongly relies on differentiating $e(t,x)=N_E(\rho (t,x),\gamma (t,x))$ with respect to *t*. In the case $2\leqq d\leqq 4$, this can be avoided since we have the curvature estimates

7.8 $$\begin{aligned} \text {(a) } t\mapsto \gamma (t,x) \text { is convex \quad and \quad (b) } t\mapsto \rho (t,x) \text { is concave}, \end{aligned}$$

where we used $d\leqq 4$ in (5.12) for (b). With (a) and (b), we can further exploit

(i) the convexity of $N_E$,
(ii) the monotonicity of $\rho \mapsto N_E(\rho ,\gamma )$ (non-increasing, cf. (1.25c) for $d\geqq 2$), and
(iii) the monotonicity of $s \mapsto N_E(s^{1-4/d^2}\rho ,s\gamma )$ (non-decreasing, cf. Proposition 6.2).

Choosing $t_0,t_1,\theta \in [0,1]$ with $t_0<t_1$, we set $t_\theta :=(1{-}\theta )t_0+\theta t_1$ and have to show

7.9 $$\begin{aligned} N_E(\rho (t_\theta ),\gamma (t_\theta )) \leqq (1{-}\theta ) N_E(\rho (t_0),\gamma (t_0)) + \theta N_E(\rho (t_1),\gamma (t_1)). \end{aligned}$$

We start with the right-hand side and use convexity (i) first:

$$\begin{aligned} (1{-}\theta ) N_E(\rho (t_0),\gamma (t_0)) + \theta N_E(\rho (t_1),\gamma (t_1)) \overset{\text {(i)}}{\geqq }N_E\big ((1{-}\theta )\rho (t_0){+}\theta \rho (t_1), (1{-}\theta )\gamma (t_0){+}\theta \gamma (t_1) \big ). \end{aligned}$$

With the convexity (a) of $\gamma$ we have $s:=\gamma (t_\theta )/\big [(1{-}\theta )\gamma (t_0){+}\theta \gamma (t_1)\big ] \in [0,1]$ and continue

$$\begin{aligned}{} & {} N_E\big ((1{-}\theta )\rho (t_0){+}\theta \rho (t_1), (1{-}\theta )\gamma (t_0){+}\theta \gamma (t_1) \big )\\{} & {} \quad \overset{\text {(iii)}}{\geqq }N_E\big ( s^{1-4/d^2} \big [(1{-}\theta )\rho (t_0){+}\theta \rho (t_1)\big ], s\big [(1{-}\theta )\gamma (t_0){+}\theta \gamma (t_1)\big ] \big )\\{} & {} \qquad = N_E\big ( s^{1-4/d^2} \big [(1{-}\theta )\rho (t_0){+}\theta \rho (t_1)\big ],\gamma (t_\theta )\big ). \end{aligned}$$

Using the monotonicity (ii) (for $d\geqq 2$) we can increase the first argument using $s^{1-4/d^2} \leqq 1$ (because of $s\in [0,1]$ and $d\leqq 4$) and then exploit the concavity in (b) of $\rho$ (i.e. $\rho (t_\theta )\geqq (1{-}\theta )\rho (t_0){+}\theta \rho (t_1)$) giving

$$\begin{aligned} N_E\big ( s^{1-4/d^2} \big [(1{-}\theta )\rho (t_0){+}\theta \rho (t_1)\big ] , \gamma (t_\theta ) \big ) \overset{\text {(ii), (a+b)}}{\geqq }N_E(\rho (t_\theta ),\gamma (t_\theta )). \end{aligned}$$

Thus, we have proved the desired convexity (7.9) for the case $d\in \{2,3,4\}$.

## Data Availability

Data sharing not applicable to this article as no datasets were generated or analysed during the current study.
